# Structure and evolution of barley powdery mildew effector candidates

**DOI:** 10.1186/1471-2164-13-694

**Published:** 2012-12-11

**Authors:** Carsten Pedersen, Emiel Ver Loren van Themaat, Liam J McGuffin, James C Abbott, Timothy A Burgis, Geraint Barton, Laurence V Bindschedler, Xunli Lu, Takaki Maekawa, Ralf Weßling, Rainer Cramer, Hans Thordal-Christensen, Ralph Panstruga, Pietro D Spanu

**Affiliations:** 1 Department of Agriculture & Ecology, Plant and Soil Science, University of Copenhagen, Copenhagen, Denmark; 2 Department of Plant Microbe Interactions, Max-Planck Institute for Plant Breeding Research, Cologne, Germany; 3 School of Biological Sciences, University of Reading, RG6 6AS, UK; 4 Department of Life Sciences, Imperial College London, Sir Alexander Fleming Building, London, SW 7 2AZ, UK; 5 Department of Chemistry, University of Reading, RG6 6AD, UK; 6 Present address: School of Biological Sciences, Royal Holloway University of London, Egham, UK; 7 Unit of Plant Molecular Cell Biology, Institute for Biology I, RWTH Aachen University, Worringer Weg 1, Aachen, D-52056, Germany

**Keywords:** Host-pathogen interactions, Effector protein structure, Fungal proteomics, Proteogenomics

## Abstract

**Background:**

Protein effectors of pathogenicity are instrumental in modulating host immunity and disease resistance. The powdery mildew pathogen of grasses *Blumeria graminis* causes one of the most important diseases of cereal crops. *B. graminis* is an obligate biotrophic pathogen and as such has an absolute requirement to suppress or avoid host immunity if it is to survive and cause disease.

**Results:**

Here we characterise a superfamily predicted to be the full complement of Candidates for Secreted Effector Proteins (CSEPs) in the fungal barley powdery mildew parasite *B. graminis* f.sp. *hordei*. The 491 genes encoding these proteins constitute over 7% of this pathogen’s annotated genes and most were grouped into 72 families of up to 59 members. They were predominantly expressed in the intracellular feeding structures called haustoria, and proteins specifically associated with the haustoria were identified by large-scale mass spectrometry-based proteomics. There are two major types of effector families: one comprises shorter proteins (100–150 amino acids), with a high relative expression level in the haustoria and evidence of extensive diversifying selection between paralogs; the second type consists of longer proteins (300–400 amino acids), with lower levels of differential expression and evidence of purifying selection between paralogs. An analysis of the predicted protein structures underscores their overall similarity to known fungal effectors, but also highlights unexpected structural affinities to ribonucleases throughout the entire effector super-family. Candidate effector genes belonging to the same family are loosely clustered in the genome and are associated with repetitive DNA derived from retro-transposons.

**Conclusions:**

We employed the full complement of genomic, transcriptomic and proteomic analyses as well as structural prediction methods to identify and characterize the members of the CSEPs superfamily in *B. graminis* f.sp. *hordei*. Based on relative intron position and the distribution of CSEPs with a ribonuclease-like domain in the phylogenetic tree we hypothesize that the associated genes originated from an ancestral gene, encoding a secreted ribonuclease, duplicated successively by repetitive DNA-driven processes and diversified during the evolution of the grass and cereal powdery mildew lineage.

## Background

The powdery mildew fungus *Blumeria graminis* is an obligate biotrophic pathogen of cereals. It has significant impact on cereal crops that are central for food security such as wheat (*Triticum aestivum*) and barley (*Hordeum vulgare*) and is an experimental model for powdery mildew fungi in general as well as for other obligate biotrophic plant pathogens [1]. Here we research the barley pathogen *B. graminis* f. sp. *hordei*. Its infection process starts when a spore lands on a leaf, germinates, forms an appressorium and attempts plant cell penetration. The penetrating hypha produces a specialized feeding organ, the haustorium, in the host epidermal cell. The haustorium remains surrounded by a plant-derived extra-haustorial membrane. Between the haustorium and the extra-haustorial membrane there is an extra-haustorial matrix, which is the interface between the two organisms. Both the plant and the fungus are dedicated to secretory warfare and the extra-haustorial matrix is believed to represent a major battleground [2]. Effector proteins are defined as molecules that alter host cell structure or function, and thereby facilitate infection and/or trigger defence responses [3]. Effectors are therefore assumed to be secreted by the pathogen. In plant pathogenic fungi, they are broadly divided into apoplastic and cytoplasmic effectors depending on their final destination in the host. Apoplastic effectors often exhibit inhibitory activity against extracellular host hydrolytic enzymes (e.g. proteases) and are typically small and highly cysteine-rich secreted proteins [4]. Most cytoplasmic effector proteins have been identified through their avirulence functions, i.e. based on their genotype-specific recognition by matching plant resistance (R) proteins. Little is known about their direct host targets; some have a functional nuclear localization signal (NLS) suggesting a nuclear target [5]. Godfrey and co-workers recently identified 107 effector candidates based on a cDNA library prepared from barley epidermis containing haustoria [6]. All these effector candidates share an N-terminal amino acid motif named YxC, consisting of a conserved aromatic amino acid (Y, F or W) followed by any amino acid and then a cysteine. Seventy-one of these *B. graminis* effector candidates were verified experimentally in the haustorial proteome present specifically in the epidermis of infected plants, of which 51 contain the YxC motif [7,8]. The observation that only three candidate effector proteins were found in the proteome of isolated haustoria, in the preparation of which secreted proteins are mostly likely to be washed away [9], provides indirect evidence that these candidate effectors are indeed secreted by the fungus. As one outcome of the recent sequencing of the *B. graminis* genome, we reported the annotation of 248 Candidates for Secreted Effector Proteins (CSEPs), de-fined as proteins with a predicted signal peptide, but no transmembrane domain and no homology to proteins outside the Erysiphales (powdery mildews) [10]. Here we provide a global survey of the CSEPs in the *B. graminis* genome, transcriptome and proteome. We studied their predicted structures and putative functions, and explored evidence for selection acting on their diversification. Based on the results of these analyses we discuss how these key proteins may have evolved in the interplay with the host systems.

## Results

### Genome annotation and family clustering of CSEP paralogs

**Figure 1:**
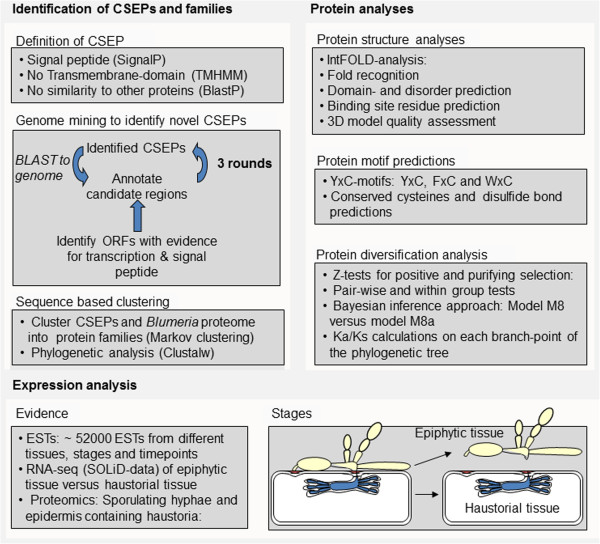
**Summary of bioinformatics and expression analysis of *CSEPs. ***Identification of CSEPs and family assignment. Workflow used to find and annotate *CSEPs* in the genome of *B. graminis* f.sp. *hordei*. After three iterative rounds of BLAST and annotation, genes were clustered into families as described in Methods. Protein analyses. The proteins predicted to be translated from the *CSEP* ORFs were analyzed to infer their 3D structure and presence of conserved motifs. These were then used to investigate evidence of different selection pressures during the course of evolution of the CSEP families. Expression analysis. Evidence to support the existence of *CSEP* genes was obtained from ESTs in the public databases, from RNAseq surveys and from the analysis of the proteomes of *B. graminis* and *B. graminis*-infected barley tissues by mass-spectrometry.

**Figure 2:**
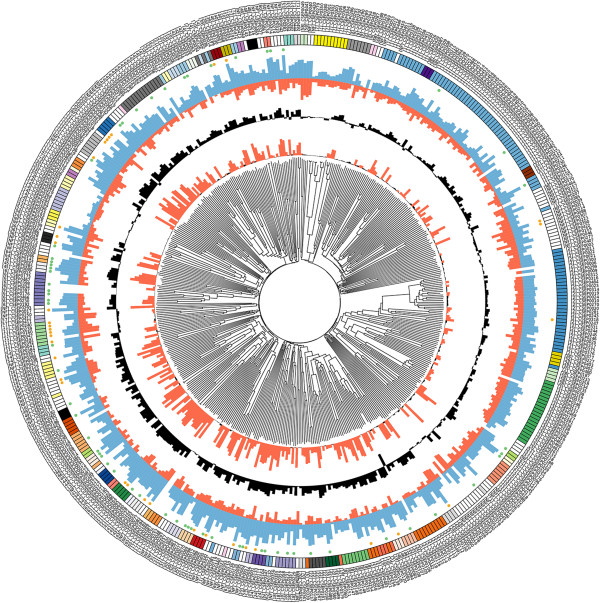
**CIRCOS plot of the CSEP superfamily with expression and proteome data.** From the perimeter to the centre: The outer ring identifies the CSEPs. The rectangles in the circle immediately below the identifiers are colour-coded: CSEPs of the same families have the same colour. The small circles below the family identifiers indicate the proteins identified by mass spectrometry in infected epidermis only (green) or in both infected epidermis and epiphytic hyphae (yellow). The first and second data histogram circles shows the expression values of the haustorial samples (blue) and of the epiphytic samples (red) of each *CSEP* gene on a log_2_ scale. The third data histogram (black) represents the ratio of the expression values in the two stages plotted on a log_2_ scale. The fourth data circle indicate the statistical significance of the ratios (red, significant/black non-significant). At the centre is a dendrogram based on the neighbour-joining dendrogram of all CSEP paralogs.

Initially, we aimed at determining a comprehensive set of all *B. graminis* CSEPs. To achieve this, we followed two complementary strategies: We first mined the *B. graminis* genome by iterative BLAST searches using previously identified CSEPs as query sequences [10]. We then performed open reading frame (ORF) prediction in combination with SignalP analysis based on whole transcriptome shotgun sequencing (RNAseq) data (Figure 1). After three rounds of iteration we identified 491 manually annotated CSEPs including the 248 predicted previously [10] (Additional file 1). Based on Markov Clustering (MCL) analysis, 407 of the predicted 491 CSEPs were grouped into 72 families (BLASTP threshold e<10^-10^; Table 1, Additional files 2 and 3). Approximately 50% of the families have two to ten members (including a total of 242 CSEPs), and seven families are comprised of eleven or more members (representing 165 CSEPs). CSEPs make up a considerable proportion of the large protein families in *B. graminis*, as this fungus only has a total of 25 protein families of ten or more members estimated by MCL-based clustering of the entire theoretically determined *B. graminis* proteome [10]. Most families harboring CSEPs are nearly exclusively comprised of effector candidates, but the largest CSEP-containing family encompasses only ~50% CSEPs. The remaining members of the latter family lack a significant SignalP score for a canonical N-terminal signal peptide, suggesting either false-negative predictions or functional diversification within this protein family. In addition to the MCL-based family grouping, we conducted neighbor-joining phylogenetic analysis of the 491 CSEPs and established a CIRCOS plot [11] (Figure 2), which illustrates their relatedness as a dendrogram. Owing to the high sequence diversity amongst the CSEPs this approach does not accurately resolve their phylogenetic relationships, but rather visualizes clusters of similar sequences within the CSEP superfamily. Bootstrap analysis indicates largely reliable family classification, while the relatedness of the families amongst each other is less well determined (Additional files 4, 5 and 6). The clades resolved by two independent methods (MCL clustering and phylogenetic classification) are largely congruent, indicating robustness of the overall family groupings. Even though, by the definition used in the context of this study, the CSEPs do not have evident homologs outside powdery mildew fungi, we inspected their amino acid sequences for signatures of known protein domains. InterProScan analysis combined with gene ontology (GO) categorization revealed that 54 CSEPs are similar to proteins with RNA binding and/or ribonuclease activity (see below), while nine have predicted coiled coil domains (Additional file 1). Such similarities to RNA binding/ribonuclease activity were found in 27 of the 32 members of MCL family 2 and in 11 of the 20 members of MCL family 3. The remaining 16 of these CSEPs are scattered across eleven other families (Additional files 5 and 7). We then searched for CSEP homologs within the Erysiphales by examining the proteomes derived from sequenced genomes of *Golovinomyces orontii* and *Erysiphe pisi* representing two other genera in this order [10]. This revealed that 16 of the *B. graminis* CSEPs are similar (TBLASTN, e<10^-05^) to proteins encoded by *Golovinomyces orontii* (two), *Erysiphe pisi* (four) or both (ten). Interspecies amino acid sequence identities of these ranged from 31% to 73% with an average of 48%. These 16 *B. graminis* CSEPs are mainly unrelated singletons, widely distributed across the phylogenetic tree (Additional file 6). This result is consistent with the previous analysis of 248 CSEPs [10], and it further underscores rapid evolution and diversification of *CSEPs* in powdery mildew genomes.

**Table 1:** Summary of the 35 largest CSEP families

**Family**	**Number of members**	**Motif**^**1)**^	**C-term. cysteine**^**2)**^	**Conserved cysteines**^**3)**^	**Average peptide length**^**4)**^	**Haustoria/ Epiphytic exp-ratio**^**5)**^	**Preference for haustoria expression**^**6)**^	**Positive selection: Pairwise z-test**^**7)**^	**Positive selection: Average Ka/Ks-values**^**8)**^
1	59		no	3	326	15	5	18 / 1711	0.68
2	32	FxC	no	4	396	6	0	3 / 496	0.73
3	20	F/YxC	no	10	384	6	0	0 / 190	0.70
4	19	FxC	yes (4–14)	2	153	58	16	8 / 171	1.20
5	15	F/Y/(H)xC	no	2	107	94	13	9 / 105	1.41
6	10	YxC	yes (1–9)	4	311	21	50	1 / 45	1.04
7	10		no	none	131	3	0	1 / 45	0.62
8	8	YxC	yes (4)	2	118	25	0	**11 / 28**	1.80
9	8		no	none	178	16	0	0 / 21	0.52
10	7	FxC	no	2	160	37	57	1 / 21	1.31
11	7		no	2	164	0	0	0 / 21	0.92
12	7	F/YxC	yes (10)	2	125	207	0	5 / 21	1.19
13	7	F/Y/(H)xC	yes (4)	2	123	30	14	**9 / 21**	1.69
14	7	YxC	no	2	127	0	0	0 / 21	0.50
15	7	YxC	no	4	310	3	0	1 / 21	0.82
16	6	FxC	no	2	144	55	0	2 / 15	1.59
17	6	F/YxC	no	5	395	11	0	0 / 15	0.56
18	6		no	2	153	74	50	0 / 15	0.97
19	5	F/YxC	yes (4)	2	115	87	0	1 / 10	0.93
20	5	FxC	yes (4)	2	128	168	0	0 / 10	0.92
21	5	WxC	yes (6)	2	118	156	20	**9 / 10**	2.76
22	5	YxC	yes (4)	2	105	31	20	2 / 10	2.00
23	5	YxC	yes (4)	2	107	197	20	**8 / 10**	2.61
24	4	F/YxC	yes (1–2)	2	125	403	50	1 / 6	0.80
25	4	YxC	yes (3–9)	2	132	168	25	**3 / 6**	1.56
26	4	YxC	yes (6)	8	392	6	0	0 / 6	0.68
27	4	F/YxC	no	3	162	10	0	0 / 6	0.90
28	4	FxC	no	5	373	5	0	0 / 6	0.83
29	4	FxC	yes (5–7)	2	115	67	0	**3 / 6**	2.09
30	4	YxC	yes (4)	3	121	101	75	**4 / 6**	2.45
31	4	YxC	yes (4–7)	2	119	141	25	0 / 6	1.37
32	4	F/YxC	yes (4)	2	112	168	0	**2 / 6**	1.35
33	4	YxC	no	2	150	0	0	0 / 6	1.03
34	4		no	none	122	369	25	1 / 6	1.68
35	4		no	4	126	not calculated	25	0 / 6	1.07

The 35 CSEP families with four or more members. The table summarizes data from gene- and protein expression as well as protein length, conserved cysteines, YxC-motif and detection of positive selection within each family. Data of families showing positive selection in the overall z-test are indicated in bold fonts. A table with more extensive details of the CSEP family analysis is provided in the Additional Files (Additional file 1). 1) Indicates the type motif in the N-terminus of the mature protein 2) The presence of a cysteine close to the C-terminus and the distance to the C-terminus is indicated in brackets 3) Number of conserved cysteines in the mature protein. 4) Length of proteins (number of amino acid residues): The average lengths of the proteins were calculated for each family. 5) Gene expression ratio in haustorial samples versus epiphytic samples, calculated as averages for each family. 6) Percentages of CSEPs in each family found only in haustoria-samples by proteome analysis. 7) Numbers of pairs with significant positive selection (z-tests at 5% level) compared to the total number of pairs within each family. 8) Family averages of mean Ka/Ks-values calculated on the mature proteins.

### Most *CSEPs* are predominantly expressed in haustoria

Many (51%) of the *CSEPs* are represented in Expressed Sequence Tag (EST) collections available in databases (Additional files 1 and 8). The EST sets with the highest proportion of *CSEP* transcripts are derived from cDNAs from haustoria-containing epidermis [6] and 162 CSEPs (33% of the 491) were found in these EST collections (Additional file 1, column W and Z). In order to further characterize the *CSEP* expression patterns and validate their annotation, we analyzed RNAseq data obtained from two separate *B. graminis* tissues isolated at 5 d after inoculation: (1) haustoria-containing plant epidermis and (2) epiphytic structures [10]. Ninety-seven percent (477/491) of the *CSEP* show clear evidence of expression in one or both of these structures (Figure 2). Expression ratios in haustoria versus epiphytic structures revealed that most *CSEP* RNAs were significantly more abundant in the haustorial samples (Figure 2 and Additional file 9), including 216 out of 349 *CSEP* RNAs that were at least 10-times more abundant in haustoria (e.g. the *CSEP* RNAs in families 1 and family 2, Figure 2). By contrast, in families 7, 11, 57 and 68 the majority of genes showed similar transcript levels in both fungal tissues. Interestingly, the *CSEP* RNAs not assigned to specific families (singletons) were also expressed at similar levels in the two samples. A large-scale mass spectrometry-based proteomics approach allowed us to map peptides derived from expressed proteins on the *B. graminis* genome, validating ORF models through the experimental evidence of protein accumulation [8-10]. Moreover, revisiting the previously analyzed proteomes of haustoria-containing epidermis and epiphytic structures [8], using updated genomic information, revealed the existence of 97 CSEPs at the protein level (Figure 2 and Additional file 1). Of these, 62 CSEPs (64%) were only detected in the haustoria-containing epidermis.

### Selection for diversity has operated in the evolution of *B. graminis CSEPs*

**Figure 3:**
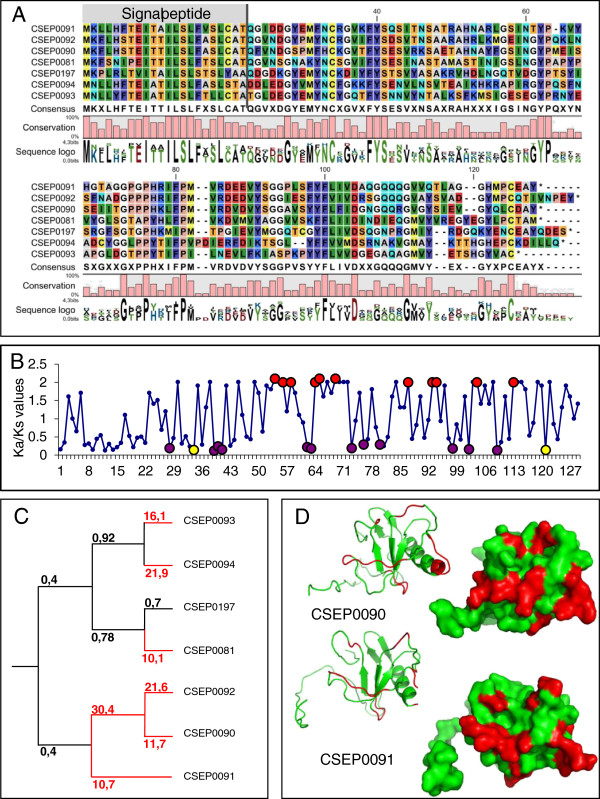
**Protein structure and positive selection in CSEP family 12.A**: Amino acid alignment of the seven members obtained with CLC main workbench (see Methods). **B**: Evidence for selection on the paralog members of family 12 was estimated using the Selecton server ( [49,50]; http://selecton.tau.ac.il/). Codon sites under positive diversifying (red) or purifying (purple and yellow) selection and conserved cysteines (yellow) are indicated by coloured circles. **C**: Cladogram with Ka/Ks-values indicated for the individual branches calculated using the on-line server at http://services.cbu.uib.no/tools/kaks. Branches in red indicate a significant positive selection. **D**: 3D protein models of two family 12 members are shown and the amino acids under positive diversifying selection are highlighted in red.

**Figure 4:**
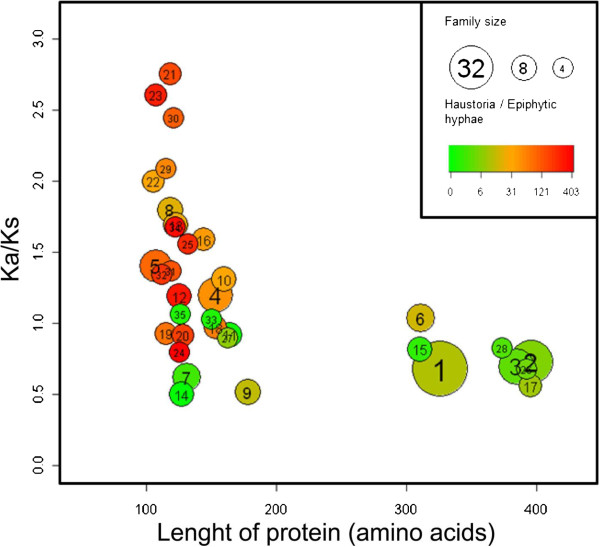
**The relationship between the length of CSEPs, the degree of positive selection and the ratio of expression in haustoria compared to expression in epiphytic hyphae.** Ratio of the non-synonymous to synonymous substitutions (Ka/Ks) within CSEP families is plotted against the length of the proteins. The values of the parameters for the axes were calculated as family averages; the family numbers are indicated in the circles. The ratios of *CSEP* expression in haustoria and epiphytic hyphae are as indicated in the colour bar. The diameter of the circles indicates the relative size of the families.

We analysed nucleotide sequence diversity in coding and non-coding sequences of *CSEP* paralogs. Unexpectedly, we observed that many *CSEPs* diverged more markedly in the coding regions. For example, we noticed that the sequences in families 8 and 30 are strikingly more different in the two exons than in the intron and the up- and down-stream non-coding regions (Additional file 10). We then used three different approaches to test whether positive diversifying or purifying selection has operated during the evolution of related *CSEPs* (Table 1, Figure 3B and 3C, Additional files 3 and 11, 12 and 13; see also Methods for experimental details). This analysis assessed which amino acids in a family varied by random drift from those that have been subjected to purifying or diversifying selection. We expected purifying selection in the N-terminal signal peptide domains based on the need to maintain secretion and considered this as a positive control in our analyses. In general, there was good agreement between the three types of approaches. However, in some cases, we only found substantial evidence of positive diversifying selection for a small number of codons, and this was often not sufficient to make the z-test based on the entire ORFs significant. Overall, we found strongest evidence for diversifying selection in families 21, 23, 25, 29, 30, 50, but also statistically significant diversification in families 5, 8, 12, 13, 16, 22, 32, 34, 42, 44, 46 and 49 (Table 1 and Additional file 3). In these 18 families, representing 93 members, diversifying selection was a general trend. However, even in the three largest families, where purifying selection was dominant, there are individual residues that seem to have been under diversifying selection. Especially in family 1, there is a stretch of 20 amino acids showing signs of positive selection, while family 2 has 16 codons in several domains with diversifying selection scattered along the protein (Additional files 12 and 14). We then analyzed the relationships between CSEP length, the degree of inferred positive diversifying selection and transcript accumulation in haustoria based on the respective average for each family. We found that the CSEP families form two clearly separated groups: one group contains shorter proteins with a preference for haustorial expression and with most families showing strong evidence of positive diversifying selection. The second group includes longer proteins with less evidence of differential expression and less overall positive diversifying selection (Figure 4). The analyses also demonstrate that there has been purifying selection in all families. This is expected especially in the region encoding the predicted signal peptides and in some conserved motifs or specific amino acids (Additional files 12 and http://13). In some families (7, 9, 14, 17 and 26) we detected only purifying selection. In other examples (e.g. family 6), the N-terminal part of the mature proteins is highly conserved and has been under strong purifying selection, while the C-terminal region appears to have been under positive diversifying selection. The most conserved amino acids are generally proline, glycine and cysteine (Figure 3A, Additional files 12, 13 and 14). These amino acids confer structural properties to proteins by providing fixed angle bends, sharp angle bends and opportunity for disulphide bonds, respectively.

### Protein structural analyses

Since by selection the CSEPs had little sequence similarity (BLASTP, e<10^-05^) to any protein previously structurally or functionally characterized, we carried out structural annotation using protein fold recognition methods [11], to search for potential relationships based on predicted structure (Additional file 15). The results of this structural annotation indicate that all CSEPs had comparatively low values for mean lengths, mean proportion disorder, mean maximum length of disorder, mean model quality and mean number of domains (Additional file 16). For example, compared with a random set of 71 proteins only detected in the epiphytic hyphae, all values are significantly lower in the CSEPs. When we control for length, all values are lower except for length and number of domains. Overall there are no statistically significant differences (apart from three-dimensional (3D) model quality) between CSEPs, proteins found in haustoria and infected epidermis and known fungal effector proteins. Ribonuclease template assignments are significantly overrepresented in CSEPs compared with all other sets apart from proteins found in haustoria and infected epidermis (i.e. sets known to include CSEPs) and in a set of 71 proteins selected at random from yeast, where there is no significant difference with this data (Additional file 17). Furthermore, assignments to hydrolase templates are significantly overrepresented in CSEPs compared with proteins from hyphae and a random set of yeast proteins (Additional file 17). We mapped the position of the residues predicted to be under statistically significant positive diversifying selection pressure onto the predicted 3D models of the proteins. We observed a variety of scenarios exemplified by the following case studies. The highest quality 3D models generated for CSEP family 12 (Figure 3 and Additional file 11) are all predicted to have approximately similar folds and were generated using ribonucleases as the top identified structural templates. The positions of the residues calculated to be under positive diversifying selection mostly occupy the surface of the globular structures (Figure 3 and Additional file 18A, right hand side images). Furthermore, these amino acids are located mostly within the loop regions of the structures, whilst the α-helix and β-strand secondary structural elements (Additional file 18A, left hand side images) are more conserved and contain residues under purifying selection. In other cases (for example family 21; Additional file 18B), the more variable regions are located in α-helices and β-strands and residues under diversifying selection are buried in regions more likely to lead to changes in folding (Additional files 11 and 18B). The models generated for other families are shown in Additional files 18C-E (see also http://www.reading.ac.uk/bioinf/CSEPs/).

### Most CSEPs harbor conserved cysteines, including N-terminal YxC-motifs, and most of these are predicted to form disulphide bonds

We compared the CSEPs against the known fungal effectors and *B. graminis* proteins that were found only in haustoria and infected epidermis, as well as the yeast and hyphae controls sets. The parameters measured were: length (as a control), amino acid frequency (A-Y), coiled-coil composition, transmembrane (TM) helix composition (as a control), low complexity regions, frequency of helical residues, frequency of strand residues and frequency of loop residues. We found that particular amino acids (C, F, H, I, N, S, and Y) and loop residues are significantly overrepresented in CSEPs, while several other amino acids (A, D, E, G, and K) are significantly underrepresented (Additional files 19 and 20). The similarity between CSEPs, known fungal effectors and *B. graminis* proteins found only in haustoria and infected epidermis is particularly striking with regard to the significantly higher frequency of cysteine residues. Manual inspection of multiple amino acid alignments of the CSEP families revealed that the cysteines are generally conserved and most families (27 out of the 35 largest) had an even number of cysteines (Table 1).

Many CSEPs (307; 63% of the 491) contain the previously described YxC-motif within the first 30 amino acids of the mature protein sequence (i.e., from which the N-terminal signal peptide was removed; Additional file 21) [6]. The frequencies of the three variants of this motif, YxC, FxC and WxC, are 47%, 49% and 4%, respectively. Of the 184 CSEPs without an N-terminal YxC-motif, there are 34 without any cysteine in the mature protein. In the remaining 150 CSEPs, 44 have a YxC-motif further towards the C-terminus. The latter is typical of the longer CSEPs (Additional file 21). Most CSEPs contain a cysteine close to the C-terminus. For example, 83% of the 307 CSEPs with an N-terminal YxC motif also have a cysteine within the last 30 C-terminal amino acids. In 65% of those, the cysteine occurred within ten amino acids from the C-terminus, preferably in positions four to seven (Additional files 14 and 22). Of the non-YxC CSEPs, only 26% have a cysteine within ten amino acids from the C-terminus. The majority (90%) of all CSEPs have at least two cysteines and thus in principle they have the capacity to form a minimum of one disulphide bond. This overrepresentation of cysteines and their conserved pattern prompted us to predict disulphide bonds using the tool Disulfide Bonding State and Connectivity Predictor “Disulfind” [12], which previously has been used for prediction of disulphide bonds in effector candidates [13]. We found that 69% of all possible disulfide bonds are predicted to be formed (Additional file 22).

### Many CSEPs show relatedness to ribonucleases

**Figure 5:**
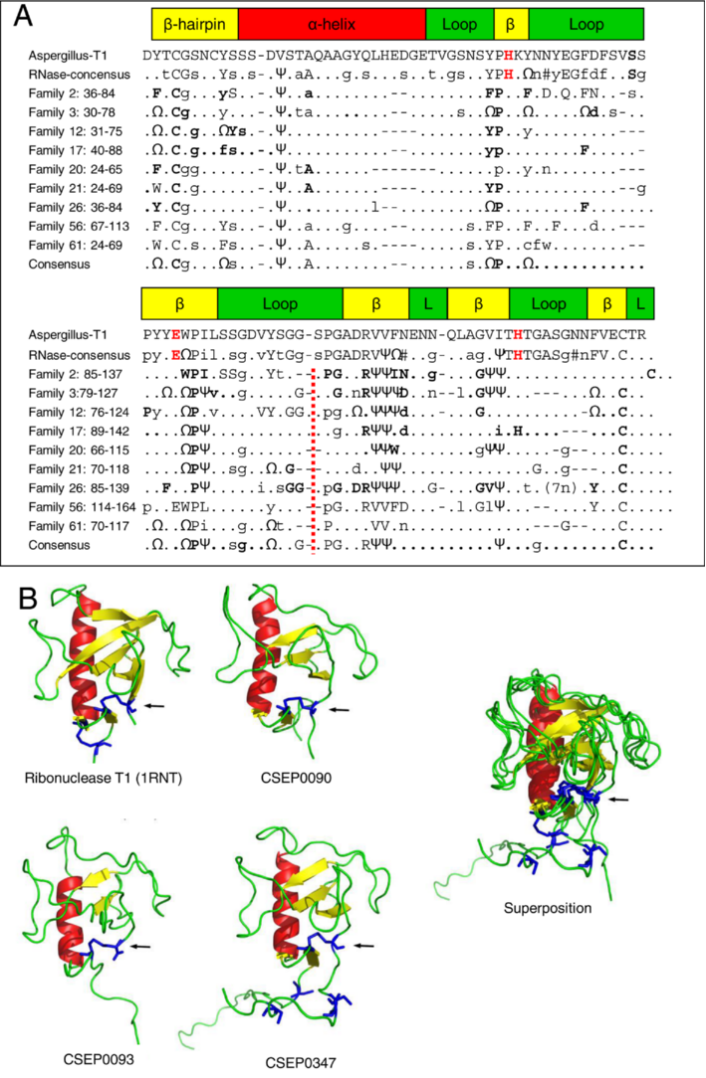
**Multiple sequence alignment and 3D models of ribonucleases and CSEPs. A**: Multiple sequence alignment of ribonuclease T1 from *Aspergillus oryzae*, a ribonuclease consensus sequence and selected CSEP family consensus sequences. The ribonuclease consensus was derived by aligning ribonucleases from *Aspergillus phoenicis* (P00653, *Penicillium brevicompactum* (P07446), *Grosmannia clavigera* (EFX05096), *Phaeosphaeria nodorum* (XP_001800520) and *Mycosphaerella graminicola* (EGP89360). The alignments were manually edited based on MultAlin-alignments (http://multalin.toulouse.inra.fr/multalin/multalin.html). The CSEP families included are primarily those showing most ribonucleases identified by InterProScan or by the structural annotation. The secondary structures (α-helix, β-sheets and loops) of ribonuclease T1 from *Aspergillus* shown on top are according to Pace et al. [54]. Catalytic active site residues in ribonucleases are indicated in red. Intron position is indicated by a red vertical dashed line; there is one exception, one member of family 56 does not have this intron. Amino acid numberings are the ranges for each family. Upper case letters indicate highly conserved positions, while lower case letters indicate that the positions are present in some of the family members only. Omega (Ω) is used for aromatic amino acids (F, Y and W), and psi (Ψ) is used for V, L and I. Letters in bold indicate that the positions are under purifying selection. Dots indicate non-conserved positions and dashes are gaps. **B**: 3D models of ribonuclease T1 and three CSEPs and their superposition. Arrows indicate the predicted disulphide bonds between the N- and C-terminal cysteines.

The InterProScan analysis revealed that 54 CSEPs (57 proteins in the entire *B. graminis* proteome) show affinity to ribonucleases/ribotoxins (see above, IPR016191). Also the IntFOLD structural analysis (see above) indicated that many CSEPs matched ribonuclease structural templates, particularly those of two well-characterized ribonucleases: T1 from *Aspergillus oryzae* (11-times) and U2 from *Ustilago sphaerogena* (16-times; Additional file 7). Thirty-seven CSEPs from many different families have top models of medium or high score in this category. Thus, two fundamentally different but complementary methods (InterProScan and IntFOLD) indicate the relatedness of a considerable subset of the CSEPs to ribonucleases. Based on the two procedures, we found that, across the phylogenetic tree, a total of 72 CSEPs, representing 15 different families and seven CSEPs not assigned to families, show similarity to ribonucleases, of which 35 were predicted by InterProScan, 18 by IntFOLD and 19 by both approaches (Additional file 7). We aligned consensus sequences obtained from nine of these CSEP families with the well-described *Aspergillus* T1 ribonuclease and a consensus sequence generated from several other ribonucleases. In this multiple sequence alignment, we observed considerable similarity between CSEPs and ribonucleases at the level of the primary amino acid sequence, and we identified approximately eight to nine positions that are highly conserved (Figure 5A). Moreover, the intron between the first and second exon of the ten *CSEP* families is at the same relative position. The predicted folds of some of the CSEPs are highly similar to that of ribonuclease T1 (Figure 5B) showing that, even though their amino acid identities are only about 20%, their predicted 3D structures are well conserved. It is also noteworthy that the native ribonuclease fold includes a disulphide bond as predicted in many of the CSEPs (see above), further strengthening the degree of similarity between these proteins. Overall, this suggests that these families may have a common origin. Despite the similarities, it seems likely that the ribonuclease activity was lost in these CSEPs, since well-known active site residues are absent (Figure 5A).

### *CSEP* family members cluster in the *B. graminis* genome

**Figure 6:**
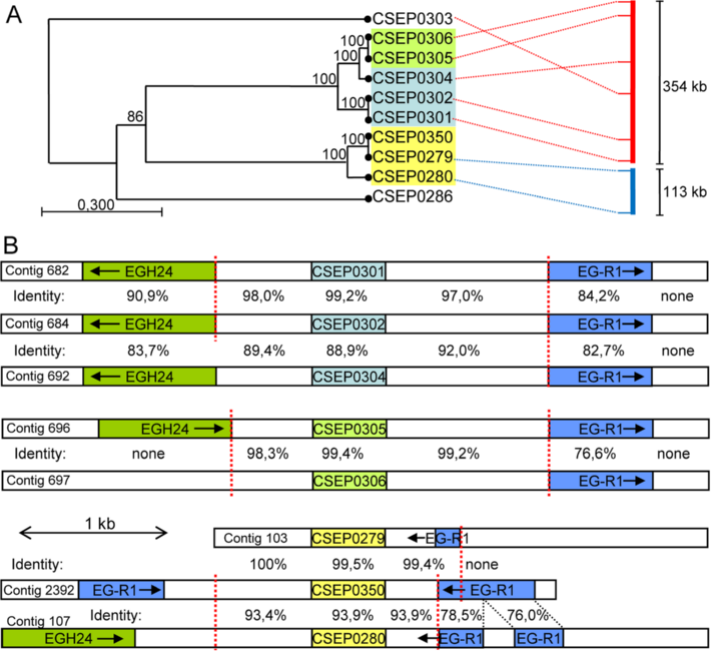
**Genome clustering of eight *CSEP *paralogs from family 7 on two sequence scaffolds. A**: Correlations between the phylogenetic relationships based on nucleotide sequences of *CSEP* paralogs from family 7 and their locations on the genomic sequence scaffolds 005496 (red) and 005502 (blue) indicated with dotted lines. Only the relevant parts of the sequence scaffolds (scale bar) are shown. The colour code of the *CSEPs* refers to the genomic organization shown in panel B. **B**: Schematic illustration of the genomic organization (encompassing about 5 kb) of the eight *CSEP* paralogs with the retro-transposable elements *Egh24* and *Eg-R1*[15,16]. The percentages of nucleotide sequence identity in pairwise comparisons are indicated and abrupt changes in sequence similarity are indicated with vertical dashed lines in red. The colour code of the *CSEPs* refers to the phylogenetic tree shown in panel A. Scale bars represent lenghts of DNA in base pairs.

The existence of discernible CSEP families suggests frequent gene duplication events during evolution of the *B. graminis* genome. To obtain clues about the underlying molecular mechanisms, we studied how *CSEPs* are organized in the genome. We analyzed in detail the distribution of 252 *CSEPs* belonging to 22 families, including the 16 largest families. We found that 207 genes (82%) are clustered family-wise on individual genomic sequence scaffolds (Additional file 23). In some families most or all genes reside on a single scaffold. For example, six out of the seven genes in family 9 cluster on one scaffold, and in family 2 we found that 18 out of the 32 genes cluster on one scaffold within 1429 kb (Additional file 24). The clusters with 2–18 members are on average 434 kb long and the mean distance between clustered *CSEPs* is 129 kb. In 13 cases gene-pairs are direct neighbors, separated by only 2–6 kb. Surprisingly, however, the most closely positioned gene pairs do not always encode the most closely related CSEPs. A comprehensive analysis of the distribution of all *CSEPs* showed that they are spread throughout the genome (Additional file 25), but two thirds of the 455 *CSEPs* located on 43 major sequence scaffolds were clustered family-wise (Additional file 25). The *B. graminis* genome is very rich in repetitive DNA sequences [10,14] and two very frequent and widespread retro-transposons, *Egh24* and *Eg-R1*, were previously characterized [15,16]. During genome annotation, we often noticed that *CSEPs* are embedded in regions flagged as repetitive DNA [10]. We further studied the three *CSEP* families already found to have highly similar 500 bp regions upstream and downstream of their exons in order to investigate how far the sequence similarities extend (Figure 6 and Additional file 26). Six of the ten family 7 members cluster on the same sequence scaffold as three pairs with more than 99% identity within the pairs, indicating recent gene-duplications. The very high sequence similarity extended only 1 kb or less up- and downstream of the coding region. Further away, most of the genes were flanked by one of the two SINE-type retro-transposons, *Egh24* or *Eg-R1*, but here the similarity is much lower than in the *CSEP* coding region and their up- and downstream regions (Figure 6). There is an abrupt change in the level of identity from approximately 97–99.5% to 90% or below at the point where the sequence of the two retro-transposons starts. This pattern indicated that local duplication events between the retro-transposons are likely to have taken place by unequal crossover, possibly mediated by the repetitive DNA sequences including a high-copy repeat previously identified in the wheat powdery mildew fungus (AJ002007.1). In addition to this case, four highly similar *CSEP* gene pairs from families 8 and 30 were analyzed for the content of the flanking genomic regions and again repetitive DNA sequences were present close to the *CSEP* genes and the patterns are to some extent conserved between the paralogs. However, in these instances it was not possible to identify an exact breakpoint using the level of sequence similarity as it was for family 7 (Additional file 26). The flanking genomic regions of several other highly similar *CSEP* pairs were analyzed. In general they are surrounded by repetitive DNA sequences and regions of high sequence similarity only extended ~1 kb or less up- and downstream of the exons.

## Discussion

Here we report the identification of 491 *CSEPs* in the *B. graminis* genome, nearly doubling the number previously described [10]. We explored systematically the evidence that the proteins encoded by these genes have effector-like properties using bioinformatics tools and expression studies. This work will facilitate the future investigation of their functional relevance in the interaction between the fungus and its host barley [17]. The typically short ORFs, the unrelatedness of their gene products to known proteins and the tight association of *CSEPs* with retro-transposons (see below) render the identification of *CSEPs* in the large and highly repetitive *B. graminis* f.sp. *hordei* genome a challenging task. Although our bioinformatics pipeline converged after three rounds of iteration on a set of 491 *CSEPs*, suggesting saturation, we cannot exclude the possibility that some *CSEPs* escaped our attention and are missing in the present analysis. Since we deliberately focussed our CSEP prediction on genes coding for proteins with no recognizable counterparts outside the powdery mildews, we also cannot exclude that some proteins with N-terminal secretion signal and identifiable sequence similarity to polypeptides in other species (e.g. secreted proteases) exert an effector function during *B. graminis* pathogenesis. The clustering of CSEPs into families of paralogs (Figure 2, Additional file 4) suggests that *CSEPs* have gone through iterated rounds of gene duplications during evolution, and some of them are now amongst the largest gene families in *B. graminis*; in fact, overall *CSEPs* represent >7% of the protein coding genes in the *B. graminis* genome. This is a remarkable testimony to the importance of CSEPs in the powdery mildew fungi, particularly when taken together with the loss of a large number of conventional ascomycete genes and reduction in gene family size observed in this fungus [10]. Since very few *CSEPs* have recognizable orthologs in the genomes of the two powdery mildew fungi, *Golovinomyces orontii* and *Erysiphe pisi*, representing other genera, we conclude that proliferation of *CSEPs* occurred after the separation of *B. graminis* from the dicotyledonous plant-infecting mildew lineage some 75 million years ago [18]. *CSEPs* have since undergone rapid evolution and diversification. It will be interesting to correlate the *CSEPs* to orthologs in other *B. graminis* “*formae speciales”*, for example the wheat mildew fungus *B. graminis* f.sp. *tritici,* to relate *CSEP* differentiation with the evolution of host specificity and analyse the variation between CSEPs of extant isolates [17].

Expression of effectors in the interaction between the obligate biotroph *B. graminis* and its host is expected to occur either in the appressorium and penetration peg or in the haustorium to be able to manipulate the plant. Indeed, we found that a large fraction of the *CSEPs* showed a significantly higher expression in haustoria compared to the epiphytic tissue isolated at five days after inoculation (Figure 2). This was further corroborated by the observation that expression of more than two thirds of the 97 CSEPs identified as proteins by mass spectrometry was only detected in haustorium-containing epidermis (Figure 2). In general, there was a clear congruence between the EST, the RNAseq expression and proteome data. Haustorium-specific effector expression is expected in order to suppress defence throughout the fungal life cycle [19]. On the other hand, we also predict that some *CSEPs* may be expressed very early during penetration and these might already be present in the germinating conidia and exert their function before haustoria are formed. Unfortunately, there is only limited transcript data available from conidia at the stage of penetration, but maybe the larger CSEPs (300–400 amino acids), belonging to the three largest families and in general showing low transcript levels (Figure 4) have functions at the earlier stages during infection. Here a low relative abundance may suffice due to the narrowly focussed area at the tip of the penetration hypha where the protein needs to act.

Diversification through positive selection for amino acid changes has occurred in many of the families of paralogs and points to adaptive modifications (Figure 3 and Figure 4, Additional files 11, 12 and 13). These may have resulted in increased virulence and/or avoidance of R-protein mediated recognition. Measuring positive diversifying selection is only possible in families where the coding sequences can be aligned with sufficient confidence and many of the *CSEPs* have diverged so much that they are too different for a reliable analysis. We may therefore have underestimated the degree of positive selection across the *CSEP* superfamily. In any case, the incidence of diversifying selection found here shows that it is a widespread and fundamental process in the evolution of *B. graminis CSEPs* as also demonstrated in other plant pathogenic plant pathogens [20,21]. However, we also find families (e.g. families 7, 9, 14, 17 and 26) showing only purifying selection. In these cases, *CSEP* evolution evidently favoured conserved structures to exert their functions. The CSEP families can be grouped in two main categories based on a number of characteristics (Figure 4). One group includes approximately 180 short CSEPs. These: 1) are typically 100 to 150 amino acids long; 2) have the strongest preferential transcript and protein accumulation in haustoria; 3) often have codons that show evidence of diversifying-selection, perhaps because they may be functioning in close interaction with plant targets and the R-protein surveillance system; 4) usually have only two or three cysteines and are thus not cysteine-rich as many apoplastic effectors suggesting that they may act inside the host cells. The other group of families comprises about 140 CSEPs, including those in the three largest families (1, 2 and 3) and several smaller families, and shares the following features: 1) they are relatively long CSEPs (300–400 amino acids); 2) they do not show high preferential expression in haustoria; 3) they have few codons subjected to positive diversifying selection, but in contrast they have many codons that have experienced pronounced purifying selection; 4) they often have several conserved cysteines predicted to form disulphide bonds in an oxidizing environment. The latter characteristic might indicate that their host targets are localized in the apoplast or extra-haustorial matrix [4]. The structure prediction studies and 3D modelling showed that CSEPs generally have similar characteristics as known effectors described in other fungi [4] and the proteins are detected preferentially in *B. graminis* haustoria in infected epidermis [8]. On average, the global model quality scores for the generated 3D models were in most cases poor or low (p>0.05), which is expected since most CSEPs had distant or no detectable homology to known template structures. However, a number of the structural models were of medium (p<0.05) and high (p<0.01) confidence, and when the amino acid residues under positive selection were mapped on these models, they are often in the loop regions predicted to be exposed and thus possibly available for interactions with other proteins as part of their effector functions (Figure 3D and Additional file 18). It will be interesting to discover whether experimental determination of the 3D structure of these proteins confirms these predictions, particularly regarding the relative position of the residues under diversifying-selection.

 The cysteine frequency is higher in CSEPs than in average *B. graminis* proteins. However, it is much lower than that found among the cysteine-rich secreted proteins from *Melampsora larici-populina*[22] and apoplastic cysteine-rich effectors from *Cladosporium fulvum*[4]. The highest conserved cysteine frequency in *B. graminis* CSEPs, found in family 3, is only 2.9% and the presence of the cysteine in the YxC motif contributes significantly to the higher frequency of this amino acid in the CSEPs compared to other *B. graminis* proteins. It has been suggested that the many cysteines of the *M. larici-populina* effectors are important for the overall fold topology rather than for resistance towards degradation in the apoplast [22]. However, given the conservation of the disulphide bond in the ribonuclease fold, it is most likely that this motif is important for protein stability [23]. Our analysis shows a similar picture although in the CSEPs it is not only cysteines, but also other commonly conserved amino acids (e.g. glycine and proline) that are predicted to contribute to structural properties.

The YxC-motif, first found in *B. graminis* CSEPs [6], is a common motif among effector candidates also in rust fungi, such as *Puccinia graminis* f.sp. *tritici*[6,24], *P. striiformis* f.sp. *tritici*[25] and *Melampsora larici-populina*[13,22]. Here we showed that the *B. graminis* CSEPs have mostly an N-terminal YxC-motif, but in the longer CSEPs it can occur over the whole length of the protein. The functional significance of this motif remains elusive. However, a conserved host cell targeting sequence (RXLR-DEER) followed by C-terminal functional regions has been found in other plant pathogens, for example in the oomycete *Phytophthora infestans*[21].

Using two different approaches (InterProScan and IntFOLD) we found that 72 of the 491 CSEPs have recognizable relationships to ribonucleases (Additional file 7). This is possibly an underestimation as sequence variation in residues that are critical for the assignment to this polypeptide category will probably lead to false-negative predictions. It is interesting to note that the vast majority (54 of 57) of proteins in the *B. graminis* genome with domain IPR016191 (ribonuclease/ribotoxin) are CSEPs. There are numerous additional proteins encoded by the *B. graminis* genome that have a relationship to RNA metabolism/turnover. These include for example proteins with InterProScan domains IPR000504 (RNA recognition motif domain, 60-times present), IPR012337 (ribonuclease H-like domain, 35-times present) and IPR001247 (exoribonuclease phosphorolytic domain 1, 6-times present), but none of them is found in the CSEP set. The IPR016191 (ribonuclease/ribotoxin) domain thus seems to be a hallmark of the CSEP family, suggesting that it might be important for effector structure or activity, while other RNA binding or modifying proteins encoded by the *B. graminis* genome might have housekeeping functions.

A secreted fungal ribonuclease appears to be the common origin of many CSEPs in different families, as an alignment suggests that 10–20 spaced and moderately preserved amino acids are conserved between ribonucleases and these CSEPs (Figure 5). These amino acids are likely to play important structural roles in scaffolding the CSEPs, being located typically in the β-sheets or at the border between a β-sheet and a loop region. Meanwhile, we found that the regions with amino acids under diversifying selection are located in the loops and predicted to be exposed on the surface of the proteins. Although the ribonuclease-like proteins are unlikely to be functional as RNA-degrading enzymes since they lack critical active site residues (Figure 5A), we speculate that some of these effectors could still be involved in interactions with host RNAs and modulate host immunity via this route. Alternatively, as extracellular ribonucleases are very stable molecules, highly resistant to proteolytic degradation, they may have had a rigid structure that could have been an ideal starting scaffold for evolving an effector arsenal, in which the loop regions were subjected to positive selection allowing the CSEPs to diversify and avoid recognition by host surveillance factors (R-proteins). A similar example of structural conservation among effector candidates has recently been found by Win et al. [26], who showed that RXLR effectors of the Peronosporales (oomycetes) often share a WY-domain that is structurally conserved despite high sequence divergence between different plant pathogenic species. The genes encoding the CSEPs shown in Figure 5A have a common relative intron location, further corroborating a common ancestor. Moreover, since this intron location is also shared in many other *CSEP* genes, it may be that a large proportion of the CSEPs have evolved from an ancestral microbial ribonuclease similar to ribonuclease T1. A model for *CSEP* gene amplification was suggested based on the observation that *CSEPs* belonging to the same family are very often clustered in the genome and in several cases separated by less than 10 kb (Figure 6). This hinted that they evolved by gene duplication events due to unequal crossovers [27,28]. Also in another biotrophic phytopathogenic fungus, *Ustilago maydis*, genes encoding small secreted proteins with unknown function were found in clusters [29], even though the spacing between those effectors was much shorter, possibly reflecting the general compactness of that genome compared to that found in *B. graminis*. Illegitimate recombination was found to be the major driving force in gene duplications in plants, for instance in the evolution of multi-locus resistance genes [30,31]. This clustering is in contrast to the situation for the family of *EKA* genes, encoding another type of putative *B. graminis* effectors that have spread across the entire genome by means of a transposable element [32]. We found that *CSEP* genes often are closely associated with two well-described SINE-type retro-transposons, *Egh24*, *Eg-R1* and another high copy repeat (AJ002007.1) originally found in the wheat powdery mildew *B. graminis* f.sp. *tritici,* which are all very abundant in the genome. The genomic regions adjacent to *CSEPs* in most cases are flanked by these repetitive DNA elements and the pattern is conserved between the closest paralogs. Unequal crossover is mediated by highly similar sequences and therefore the retro-transposable elements are very good candidates for facilitating such events. In *M. larici-populina* tandem repeats of *AvrM*-paralogs are also flanked by transposable elements [22]. Powdery mildews including *B. graminis* have lost the repeat-induced point mutation (RIP) pathway [10] and this may have allowed extensive amplification of transposable elements in the genome. Our findings here suggest transposable elements have helped *CSEPs* to multiply and proliferate as described for the *EKA* effector gene candidates [32]. If this is true, then the loss of the RIP pathway and resulting retro-transposon driven genome expansion could have conferred a selective advantage and facilitated evolution of powdery mildew fungi by potentiating proliferation of effector proteins.

Here we have shown that many *CSEPs* are likely to have evolved from (an) ancestral extracellular ribonuclease(s) through a series of gene duplications followed by diversifying positive selection. A number of different models for the evolution of gene duplications have been proposed and they can be classified depending on how gene duplications affect fitness, whether there is positive diversifying or purifying selection and whether there is pre-existing allelic variation [33]. Our observation that the transcript level of many *CSEPs* was high *in planta* is consistent with the view that the expression level is important for their function. For example, many effectors work by interacting with proteins where it matters to be present in abundance to inactivate their targets. Many effectors are also exposed to proteases leading to a fast degradation, so a high transcript level will be an advantage. A gene duplication resulting in two copies will often lead to a further increase in expression through a gene dosage effect and thereby increased fitness. Once duplicated, the genes can be subject to diversifying selection: indeed we have detected pronounced diversifying selection in some families. Overall this fits with the “diversifying selection model”, described by Innan and Kondrashov [33], explaining how gene families can evolve and result in new functions for the individual members. Our work is a further illustration of how a stable structural fold may act as a template for diversification [34].

## Conclusions

This comprehensive analysis indicates that CSEPs in *B. graminis* belong to a super-family of proteins, and it has validated the view that they are candidates for important effectors of pathogenicity. The findings from this work provide a solid foundation for proceeding with a systematic functional genomics analysis [17]. Furthermore, we propose a model of how these proteins evolved from a gene coding for a secreted ribonuclease by gene duplication associated with repetitive elements generated by retro-transposon activity. Subsequent diversification yielded a diverse palette of effector functions. We speculate that powdery mildew fungi benefit from an efficient repertoire of secreted effector proteins able to suppress host defence for the benefit of the fungus.

## Methods

### Identification and MCL clustering of effector candidates

*CSEP* genes were identified by the same criteria as described previously [10], except that predicted transmembrane domains overlapping with the predicted N-terminal secretion signal were discarded due to the similarity in amino acid patterns between the two signals. New *CSEP* candidates were found by identifying and annotating genomic candidate regions based on self-BLASTs or ORF predictions. We first performed BLAST searches against the genome (TBLASTN, e<10^-05^) using the previously identified 248 *CSEPs* (excluding two *CSEPs* with high similarity to transposable elements) as a query to identify candidate regions encoding other CSEPs. These candidate regions were then manually annotated using the protocol described [10]. The above procedure was repeated with the newly identified genes until no new candidate regions without annotated genes could be found. In parallel, all predicted ORFs with an N-terminal secretion signal and transcriptional evidence were taken as candidate regions and manually annotated.

In summary, the CSEPs have to fulfil all of the following criteria:

a) Contain a secretion signal as predicted by SignalP V3.0 (D-cutoff values > 0.5).

b) Contain no predicted transmembrane domains (after removal of the first 20 amino acids)

c) Have no similarity to other proteins in the NCBI NR database (BLASTP, e<10^-05^) except for hits to powdery mildews

### Gene family prediction

The Markov Cluster Algorithm (MCL) was used to identify clusters of similar proteins based on a graph constructed by a self-BLAST of the entire proteome or the CSEPs (BLASTP, e<10^-10^). The protocol as described by Enright et al. [35] was followed with I = 2.

### Phylogenetic analysis

For the CIRCOS plot shown in Figure 2 a multiple sequence alignment of the conceptual CSEP amino acid sequences was established using ClustalW (http://www.ebi.ac.uk/Tools/msa/clustalw2/) [36]. The alignment file was used for phylogenetic analysis *via* the phylogeny option of ClustalW (http://www.ebi.ac.uk/Tools/phylogeny/clustalw2_phylogeny/). The neighbour-joining algorithm was chosen to generate a tree file that was subsequently fed into MEGA4 (http://www.megasoftware.net/) [37] for visualization. For generation of a bootstrap consensus tree, ClustalW alignment and neighbour-joining analysis (100 replicates) were performed with MEGA5.

### EST evidence for *CSEPs*

*CSEPs* were BLAST-searched against the *B. graminis* EST resources available at COGEME, the phytopathogenic fungi and oomycete EST database [38], where most *B. graminis* sequences are from conidia [39] and the data-set previously published [6,10] (Additional file 8).

### Expression analysis of *CSEP* genes

The abundance of *CSEP* RNA was determined at two stages of *B. graminis* development: haustoria in infected barley epidermis and in the epiphytic structures (e.g. surface runner hyphae, conidiophore foot cells, conidiophores, conidia) isolated at five days after inoculation of two week-old barley primary leaves. The samples were equivalent to those used in our previous publication [10] and the analysis was carried out as follows. Three independent biological replicates were used for each stage. Total RNA was extracted and partially depleted of the ribosomal RNA (RiboMinus^TM^ Eukaryote, Life Technologies, Carlsbad, CA, USA). Whole transcriptome libraries were prepared from each sample (SOLiD Whole Transcriptome Analysis Kit, Life Technologies). Libraries were barcoded and pooled together before emulsion PCR amplification. One flow-cell was loaded with 316 million beads, and 50 bp fragments were sequenced with a SOLiD version 3 instrument (Life Technologies).

### Sequence mapping

Bowtie (version 0.12.7) was used for the mapping of sequence reads to the *B. graminis* genome, using the .cfasta .qual as the input files and output piped to a Binary Alignment/Map file (BAM). Due to the highly repetitive nature of the genome, the Bowtie mapping settings were restricted to allow one mismatch and uniquely aligned reads only. The SortSam module of the Picard sequencing tools (version 1.56) was used to order the reads in the BAM files according to their genomic position. The CoverageBed utility from the BedTools (version 2.11.2) collection was used to determine the number of read counts per gene, using the *B. graminis* gene annotation in a BED format.

### Data normalization and differential expression analysis

The read counts for each of the six biological samples were imported into R statistical software (http://www.r-project.org/) and pre-processed and analyzed with the R package EdgeR. EdgeR transforms the gene expression count data to pseudo count values using a quantile-to-quantile normalization, followed by an exact test for a negative binomial distribution to determine differentially expressed genes. The p-value was corrected for multiple testing using the False Discovery Rate (FDR) using the Stats R package. Using this approach, 2110 genes out of a total of 6865 genes were found to be differentially expressed at the 1% FDR level.

### Protein sequences databases for protein identification

The genome assembly of *Blumeria graminis* f.sp*. hordei* strain DH14 [10] (http://www.blugen.org/) was used to generate a protein open reading frame (ORF) database based on the gene annotations submitted to NCBI (http://www.ncbi.nlm.nih.gov/bioproject/28821).

### Protein identification by mass spectrometry

The mass spectrometry data used for this work was acquired from in-solution tryptic digest preparations of protein extracts from two different tissues, sporulating *B. graminis* hyphae and infected barley epidermis containing *B. graminis* haustoria [8]. The data are deposited in the PRIDE database (accession numbers 26886 to 26889; http://www.ebi.ac.uk/pride/). In order to identify the occurrence of CSEPs in the relevant datasets, we re-searched Mascot generic files (*mgf) of these datasets with the Mascot search engine vs. 2.3.02 (Matrix Science, London, UK). This was done simultaneously against the following three databases: *B. graminis* protein database, *B. graminis* CSEP database, and contaminants database as described in [8], with the exception that oxidation of methionine and proline was selected as variable modification. Peptide scores and estimation of the FDR were assigned using the Percolator algorithm [40] within the Mascot software [41]. For identification, a protein required two or more unique peptides with a score above the identity score threshold (p <0.05) as calculated by Percolator. Following manual inspection, it was observed that with the exception of an actin and a glucose-6-phosphate isomerase protein, *B. graminis* proteins did not share any identified peptide sequences with proteins from the contaminants database. In the case of the actin protein, it was deduced that the protein was of *B. graminis* origin rather than from contamination since the total protein score was higher for the *B. graminis* actin than for the putative human actin contaminant.

### Protein structure and function prediction

The FASTA formatted sequence files for the CSEPs were submitted to the IntFOLD server [42], which combines a suite of advanced tools for the prediction of protein structure and function from amino acid sequence. The IntFOLD server comprises automated methods for fold recognition (IntFOLD-TS), domain prediction (DomFOLD), disorder prediction (DISOclust), binding site residue predictions (FunFOLD) and 3D model quality assessment (ModFOLD) [11,43,44]. For each protein, the PDB header files of the top structural templates were scanned for keywords referring to functions, such as RNAse, ribonuclease and hydrolase, and their frequencies were recorded. Finally, the best 3D models for the CSEP families with confident (medium to certain) structure predictions (families 5, 12, 21, 22 and 23) were downloaded from the IntFOLD server. Each model was then visually inspected and the residues that were found to be under positive selection were highlighted using PyMol (http://www.pymol.org).

### Amino acid frequencies and different sequence/structural features

The individual amino acid frequencies and their occurrence within different sequence/structural features were calculated*.* The PSIPRED secondary structure prediction method [45,46] was used to calculate the frequencies of residues in each of the secondary structure elements (helices, strands or loops). The pfilt method [47] was used to calculate the frequencies of residues in coiled-coils, the frequencies of residues in transmembrane helices and the frequencies of residues in low complexity regions.

### Functional annotation

InterProScan analysis was conducted to identify functional domains [48].

### Disulphide bond predictions

Disulphide bonds were predicted with the tool Cysteines Disulfide Bonding State and Connectivity Predictor Disulfind (http://disulfind.dsi.unifi.it/) [12].

### Comparison of CSEP prediction data with those for other protein sets

In a previous study, the IntFOLD server was used to structurally and functionally annotate proteins found in specific tissue types of *B. graminis*: the haustoria (the feeding and effector-delivery organs of the pathogen) and the sporulating hyphae*.* The *B. graminis* data were then compared with sample sequence data sets obtained from *Saccharomyces cerevisiae*[8]. In the present study, we compared the CSEP predictions with the data obtained from our previous study, as well as with a data set of known fungal effectors obtained from the literature [4]. Thus, the following sequence data sets were compared with the CSEP data: Haustoria_only, 71 *B. graminis* proteins that were found to be exclusively expressed in haustoria; Hyphae_only_random, a random sub set of 71 *B. graminis* proteins that were found to be exclusively expressed in hyphae; Hyphae_only_length_dist, a sample of 71 proteins exclusively found in hyphae with the same distribution of lengths as the Haustoria_only proteins; Yeast_random and Yeast_length_dist, as above but for subsets of proteins from yeast; Hyphae_plus_Haustoria, the subset of 194 *B. graminis* proteins found in both hyphae and haustoria tissue, Known_Fungal_Effectors, the set of 39 verified fungal effectors identified from the literature; proteome_minus_CSEPs, the *B. graminis* proteins excluding the CSEP set. The length distributions of the proteins in each subset were also visually inspected to ensure that the sampling was representative with regard to protein size. Wilcoxon signed rank sum tests and Fisher’s exact tests were carried out using R ( http://www.r-project.org) in order to measure the statistical significance of differences between the CSEP prediction data and those from each of the comparison sets.

### Tests for positive and purifying selection

Amino acid alignments of CSEPs were carried out with the CLC main workbench (Aarhus, Denmark). Positive selection was studied within the families of paralogs by three methods. Codon-based z-tests of selection both as a pair-wise analysis and as an overall analysis were done in MEGA version 5 [37] using the modified Nei-Gojobori method with the transition/transversion ratio set to 1. To identify which codon sites were under positive or purifying selection we used a Bayesian inference approach and employed the Selecton server ( [49], [50]; http://selecton.tau.ac.il/) to run model M8 [51] and when positive selection was detected to run the model M8a versus model M8 as a statistical test of significant positive selection. Finally, we used the method 7 described by Liberles [52] to calculate the ratio of non-synonymous (Ka) to synonymous (Ks) nucleotide substitution rates of pairwise combinations of genes or branches of gene phylogenetic trees, which is available on-line at http://services.cbu.uib.no/tools/kaks. This method is incorporating codon bias and focusing on the branch-points reflecting the evolution of the individual paralogs in the families, pinpointing the events of positive selection to specific branch points of the phylogenetic tree. A codon usage table for *B. graminis* was employed ( http://www.kazusa.or.jp/codon/, [53]). The sequence identities in the coding and non-coding sequences of close paralogs of family 7, 8 and 30 were calculated by comparing the genomic regions 500 bp upstream to the start codon, the exons and the intron and then 500 bp after the stop-codon.

## Endnotes

The *B. graminis* f.sp. *hordei* genome sequence has been submitted to GenBank under genome project ID 28221. Submission of a revised assembly and annotations is in progress, and will be accessible under the same project ID. Pending the completion of the submission process, the updated sequences and annotations can be accessed at http://www.blugen.org/index.php?page=data. The RNASeq analysis is available from ArrayExpress (http://www.ebi.ac.uk/arrayexpress/) under accession E-MTAB-682. The full structural annotation data relative to CSEPs is available at http://www.reading.ac.uk/bioinf/CSEPs/. Mass spectra, MASCOT and associated metadata can be retrieved from the PRIDEdatabase; http://www.ebi.ac.uk/pride/.

## Abbreviations

CSEP: Candidate for secreted effector protein; EST: Expressed sequence tag; GO: Gene ontology; MCL: Markov clustering; ORF: Open reading frame; 3D: Three-dimensional; RNAseq: Whole transcriptome shotgun sequencing; R-protein: Resistance protein; TM: Transmembrane.

## Competing interests

The author(s) declare that they have no competing interests.

## Authors’ contributions

CP, RP and PDS, designed this study. EVLvT, JCA, GB and TAB, performed the computational and bioinformatic analysis. LJM and LVB, designed and performed the structure and function predictions based on structural analysis. LVB and RC, conceived and designed the proteomics analysis. XL, TM, RW, RP and PDS, annotated the *CSEP* genes. CP, HTC, RP and PDS, wrote the paper with contributions from all the other authors. All Authors read and approved the final manuscript.

## Supplementary Material

Additional file 1 **Summary of all CSEPs.** The table includes for all 491 CSEPs various types of protein and gene expression data. The table is sorted according to the MCL family of paralogs to improve the overview of the properties of the different families. Footnotes: 1) The CSEPs described previously [10] are in light blue cells and the new CSEPs are in light red cells. 2) The gene Ids are as published [10] and in Blugen database (http://www.blugen.org) 3) Signal peptide predicted with SignalP 4) BLASTP homologies to genomic sequence data [10] 5) InterProScan gene ontologies (http://www.ebi.ac.uk/Tools/pfa/iprscan/) 6) Only those having structural models belonging to RNases are included 7) IntFOLD model scores 8) Position for the first YxC-motif in the mature protein 9) Disulphide bonds predicted using Disulfind (http://disulfind.dsi.unifi.it/). The positions are for the bond-forming cysteine pairs in the mature protein 10) The ratio of expression in haustorial epidermal strips versus epiphytic material 5 dpi determined by RNA-sequencing 11) The columns Q to Z show the presence of the CSEPs in the EST libraries described in Additional file 8. Click here for file

Additional file 2 **Size distribution histogram of MCL families.** A: Number of families with a given family size. B: Number of CSEPs in families with a given family size. Click here for file

Additional file 3 **Analysis of selection on CSEPs.** The table shows the full data set from the analyses of positive and purifying selection for all 72 CSEP families. Footnotes: 1) Indicates whether the family has the YxC-motif in the N-terminus of the mature protein. The symbol ½ indicates that some members have and others do not have the motif. 2) The presence of a cysteine close to the C-terminus and the distance to the C-terminus 3) Conserved cysteines are in the mature protein. In some cases there are a few members which are truncated and therefore lacking the terminal cysteine, but in the table it is counted anyway 4) Length of proteins: The average lengths of the proteins were calculated for each family. If the average length was below 150 amino acids, it was coloured light green, if the average length was more than 300 it was coloured grey 5) Gene expression ratio in haustorial samples versus epiphytic samples and calculated as averages for each family. Colour codes: Orange: >100x, yellow: 50-100x, light yellow: 10-50x 6) Percentages of CSEPs in each family found only in haustoria samples by proteome analysis 7) Codon-based test of positive and purifying selection. The two left columns show the numbers of pairs with significant positive selection (z-tests at 5% level) compared to the total number of pairs within each family. The two right columns show the values of P less than 0.05 that are considered significant at the 5% level (modified Nei-Gojobori (assumed transition/transversion bias = 1)). The test statistic (dN - dS) and (dS - dN) are shown for positive and purifying selection respectively. dS and dN are the numbers of synonymous and nonsynonymous substitutions per site, respectively. 8) Codon-based calculations of positive and purifying selection using the Selecton-server and based on a Bayesian inference approach [49]. The left column indicate the number of codons under positive or purifying selection. The middle column shows the significant levels of model M8a versus model M8. The right column shows the average Ka/Ks-values calculated on the mature proteins. Pink: Purifying selection Ka/Ks<0,75, yellow - orange: Positive selection, stronger colour means stronger positive selection 9) Ka/Ks-value based on method 7 of Liberles [52] and calculated by service at the Bergen Center for Computational Science (http://services.cbu.uib.no/tools/kaks). The Ka/Ks-values are calculated on each branch point on a calculated binary cladogram. Click here for file

Additional file 4 **CSEP bootstrap consensus tree showing CSEPs present in the eight largest MCL families visualized by colour codes.** Yellow - Family 1; red - Family 2; blue - Family 3; green - Family 4; purple - Family 5; light blue - Family 6; grey - Family 7; green-blue - Family 8. Numbers at branches indicate bootstrap support on the basis of 100 replicates. The scale denotes the number of amino acid substitutions per site. Click here for file

Additional file 5 **CSEP bootstrap consensus tree showing CSEPs with Blast2Go hits.** Light blue: Ribonucleases: red - coiled coil; yellow, pink and light green are other types of (uncharacterized) domains. Numbers at branches indicate bootstrap support on the basis of 100 replicates. The scale denotes the number of amino acid substitutions per site. Click here for file

Additional file 6 **CSEP bootstrap consensus tree showing CSEPs conserved in *E. pisi *and *G. orontii *.** Highlighted are CSEPs with a recognizable hit (TBLASTN, e< 10^-05^) in the *E. pisi* and/or *G. orontii* genome. Colour code: blue - *G. orontii*, yellow - *E. pisi*, green - both *G. orontii* and *E. pisi.* Numbers at branches indicate bootstrap support on the basis of 100 replicates. The scale denotes the number of amino acid substitutions per site. Click here for file

Additional file 7 **CSEPs with relationships to ribonucleases.** Seventy-one CSEPs showing relationship to ribonucleases were identified by either InterProScan analysis for the identification of functional domains or by structural annotation through analysis of structural templates from IntFOLD predictions. CSEPs are sorted according to family number. Click here for file

Additional file 8 **The *B. graminis *EST sources that provide evidence for expression of the CSEPs.** A total of almost 52000 EST sequences were searched, but some of the libraries were mixed with barley transcripts and the total number of fungal transcript therein is unknown. The number of *CSEP* in the table indicates how many of the *CSEPs* we found represented in the different EST projects. However, in many cases there were several hits, so the number of *CSEP* ESTs is much larger. The EST library with most *CSEP* hits is the epidermal EST made from epidermal cells containing many haustoria but no other fungal material [6], and here we found 151 different *CSEPs*, but the total number of *CSEP* ESTs was 1299, which was 20% of the total number of fungal transcripts. Click here for file

Additional file 9 ***CSEP *expression plot.** Plot of sorted haustorial versus epiphytic expression ratios of the 349 *CSEPs* with a ratio above 2 or below 0.5 and where the expression levels are high enough to calculate a reliable ratio. The plot shows that 216 *CSEPs* are expressed ≥10-times more in haustoria than in epiphytic tissues. The y-axis is log_10_-scaled. Click here for file

Additional file 10 **Level of diversity at the nucleotide level in pairwise comparisons between members of three CSEP families.** Diversity was calculated as percentage of different nucleotides for the two exons, the intron and the 500 bp up- and downstream to the coding region. In case there is no homology in parts of the up- and downstream regions only the homologous region was used for the calculation. Click here for file

Additional file 11 **Protein structure and positive selection in CSEP family 21.** A: Amino acid alignment of the seven members obtained with CLC main workbench (see Methods). B: Evidence for selection on the paralog members of family 21 was estimated using the Selecton server ([49,50]; http://selecton.tau.ac.il/). Codon sites under positive diversifying (red) or purifying (purple and yellow) selection and conserved cysteines (yellow) are indicated by coloured circles. C: Cladogram with Ka/Ks-values indicated for the individual branches calculated using the on-line server at http://services.cbu.uib.no/tools/kaks. D: 3D protein models of two family 21 members are shown and the amino acids under positive diversifying selection are highlighted in red. Click here for file

Additional file 12 **Distribution of codons under selection in selected *CSEP *families.** Protein sequences and distribution of the amino acids under positive and purifying selection in families 1–35. The residues are coloured according to their calculated Ka/Ks-values, estimated using the Selecton server ( [49,50] http://selecton.tau.ac.il/). Codon sites under positive diversifying (red) or purifying (purple and yellow) selection are highlighted. The conserved cysteines are shown in yellow. Click here for file

Additional file 13 **Graphs of the distribution of codons under selection.** Thirteen *CSEP* families with amino acid sites under positive selection (orange) are represented. The most conserved positions are shown in pink with the conserved cysteines in yellow. The y-axis is the Ka/Ks-value and the x-axis is the position in the protein including the signal peptide, which is mainly under purifying selection. The Ka/Ks-values were calculated using the Selecton server ( [49], http://selecton.tau.ac.il/). Click here for file

Additional file 14 **CSEP amino acid alignments of families 1–35.** The proteins are aligned using CLC main workbench, as described in Methods. Click here for file

Additional file 15 **Summary of data obtained for *Blumeria *and yeast data sets.** This compilation is based on previously published data shown in grey [8] with the new sets and measures added. The CSEPs, Known_Fungal_Effectors and Haustoria_only sets have the lowest values in terms of: mean lengths, mean proportion disorder, mean maximum length of disorder, mean model quality and mean number of domains. In addition these sets have a higher proportion of top hits to ribonuclease and hydrolase structural templates. Click here for file

Additional file 16 **Calculated p-values for unpaired Wilcoxon signed rank sum tests for the CSEP data set.** The table shows the p-values for Wilcoxon signed rank sum significant tests for the CSEP set versus all other sets according to each data type (p<0.05 highlighted green). Footnote: The null hypothesis is that the data from each comparison set is equal to or lower in value than that from the CSEP set. The alternative hypothesis is that the data in the comparison set is greater in value. Significant p-values (p<0.05) are shown in bold. Click here for file

Additional file 17 **Calculated p-values for Fisher's exact tests for the CSEP data set compared against data from all other sets.** Shown are the categorical data regarding the proportion of ribonucleases and hydrolases analysed using a Fisher’s exact test (again, p<0.05 highlighted green). Footnote: p-values (p<0.05) are shown in bold, indicating significant over representation of the data type in the CSEP set. Click here for file

Additional file 18 **IntFOLD 3D models for selected CSEP families.** A: IntFOLD 3D models for CSEPs from family 12. Positively selected residues are highlighted in red. Left, cartoon view showing secondary structure types. Right, surface view showing globular structure. Images were rendered using PyMol. B: IntFOLD 3D models for CSEPs from family 22. Positively selected residues are highlighted in red. Left, cartoon view showing secondary structure types. Right, surface view showing globular structure. Images were rendered using PyMol. C: IntFOLD 3D models for CSEPs from family 5. Positively selected residues are highlighted in red. Left, cartoon view showing secondary structure types. Right, surface view showing globular structure. Images were rendered using PyMol. D: IntFOLD 3D models for CSEPs from family 21. Positively selected residues are highlighted in red. Left, cartoon view showing secondary structure types. Right, surface view showing globular structure. Images were rendered using PyMol. E: IntFOLD 3D models for CSEPs from family 23. Positively selected residues are highlighted in red. Left, cartoon view showing secondary structure types. Right, surface view showing globular structure. Images were rendered using PyMol.A. Click here for file

Additional file 19 **CSEPs show significant differences in amino acid frequencies and secondary structure (part 1).** The CSEP set compared with other sets according to: length (as a control), amino acid frequency (A-Y), coiled-coil composition, TM helix composition (as a control), low complexity regions, frequency of helical residues, frequency of strand residues, frequency of loop residues. The Haustoria_only set is compared with other sets according to: length (as a control), amino acid frequency (A-Y), coiled-coil composition, TM helix composition, low complexity regions, frequency of helical residues, frequency of strand residues, frequency of loop residues. The null hypothesis is that the Haustoria_set has greater frequencies of that in each column than the set. Click here for file

Additional file 20 **CSEPs show significant differences in amino acid frequencies and secondary structure (part 2).** The CSEP set compared with other sets according to: length (as a control), amino acid frequency (A-Y), coiled-coil composition, TM helix composition, low complexity regions, frequency of helical residues, frequency of strand residues, frequency of loop residues. The table contains the same information as Additional file 19 but with the reverse null hypothesis (or 1-p). Click here for file

Additional file 21 **Distribution of the YxC motifs.** A: Distribution of the YxC motifs among the 307 CSEPs having this motif within the first 50 amino acids. The cumulative number of the YxC, WxC and FxC versions of the YxC-motif is plotted versus the distance of the first amino acid of the motif from the signal peptide cleavage site. B: Distribution of the YxC motifs among the 352 CSEPs having one or more versions of this motif. The cumulative number of the YxC, WxC and FxC versions of the YxC-motif is plotted versus the distance of the first amino acid of the motif from the signal peptide cleavage site. Click here for file

Additional file 22 **Cysteines and prediction of disulphide bonds in CSEPs.** A: The histogram shows the number of CSEPs versus the position of the last cysteine from the C-terminus of the protein. B: Distribution of CSEPs containing 0 – 16 cysteines. C: The histogram shows the prediction of disulfide bonds in the CSEPs using Disulfind [12]. Click here for file

Additional file 23 **Clustering of *CSEPs *on sequence scaffolds.** The Table shows for each of the studied families how many members are clustered and the length of the scaffold region containing the members. The scaffold length includes both the sum of the sequence contigs and the calculated distances between the contigs. The average distance is the distance between two *CSEPs* on the scaffold if they were distributed evenly. Click here for file

Additional file 24 **The relationship between *CSEP *clustering on genome sequence scaffolds and their sequence homology.** The Figure shows families 2–13, 15, 16, 25, 30 and 33. The scaffolds are drawn as vertical, solid bars (colours indicate separate contigs) with a scale bar in the right bottom corner. The phylogenetic tree is based on nucleotide sequences and calculated using the UPGMA algorithm with CLC Main Workbench. Bootstrap values on the basis of 100 replicates are shown at the nodes, the scale bar at the left bottom corner indicates the number of nucleotide substitutions per site. The CSEPs not connected to any scaffold with a dotted line are not found to be clustered. Click here for file

Additional file 25 **Clustering of *CSEP *genes.** A: The 68 genomic sequence scaffolds of more than 100 kb are expressed in % of their sum (92 Mb, blue line) and ordered according to their length. The 463 *CSEP*s found on each scaffold of more than 100 kb are expressed in % of their total number (green line). B: The family-wise distribution of 455 *CSEPs* on the 43 scaffolds harboring at least two *CSEPs*. Families with at least three clustered members are colour-coded so that the coloured histograms show the number of clustered members from each family on each scaffold. Click here for file

Additional file 26 **Clustering of selected *CSEP *family members.** Genome clustering of four *CSEP* paralogs from family 8 and four *CSEP* paralogs from family 30 on their respective sequence scaffolds. The schematic illustration of the genome organizations with repetitive elements is shown below each dendrogram with indications of the sequence homologies in pair-wise comparisons (note that the colour coding in the dendrogram matches the colour coding in the scaffolds). The element *Egh24* is a SINE [15], the *Bgt* repeat is an un-characterized repeat (GenBank AJ002007.1) from *B. graminis* f.sp. *tritici*, the *EKA* paralog is an *AvrA10*/*K1*-paralog [32] . Vertical dotted red lines indicate abrupt breaks in sequence homology. The scale bars next to the dendrograms refer to the genomic scaffolds. Click here for file
