# Supplementary figures and images for: Structure and evolution of barley powdery mildew effector candidates
Source: BMC Genomics. 2012 Dec 11;13:694. doi: 10.1186/1471-2164-13-694 (PMC3582587; doi:10.1186/1471-2164-13-694)

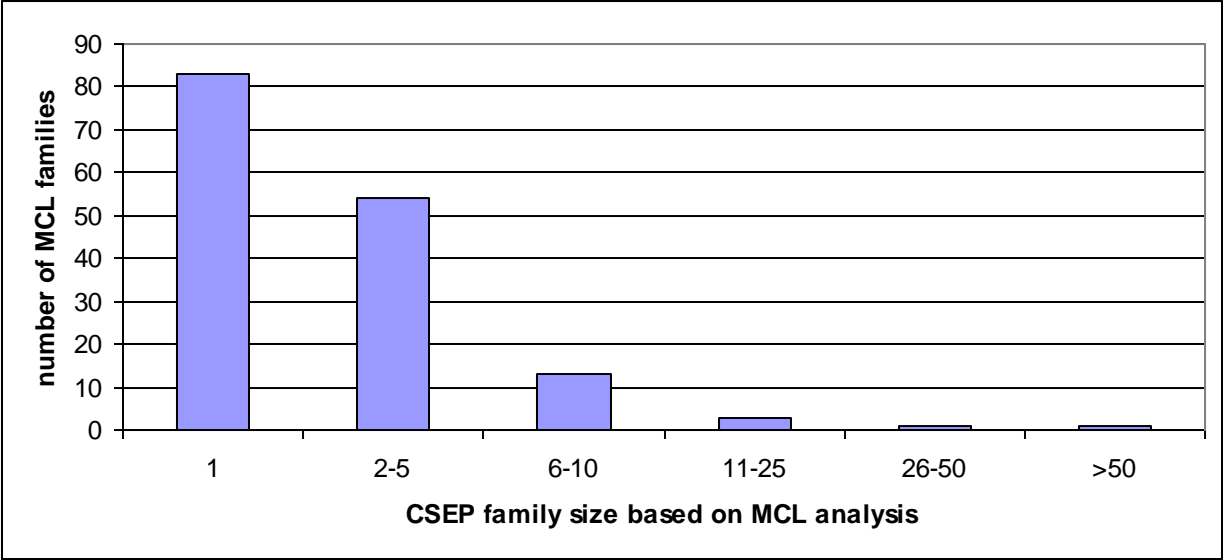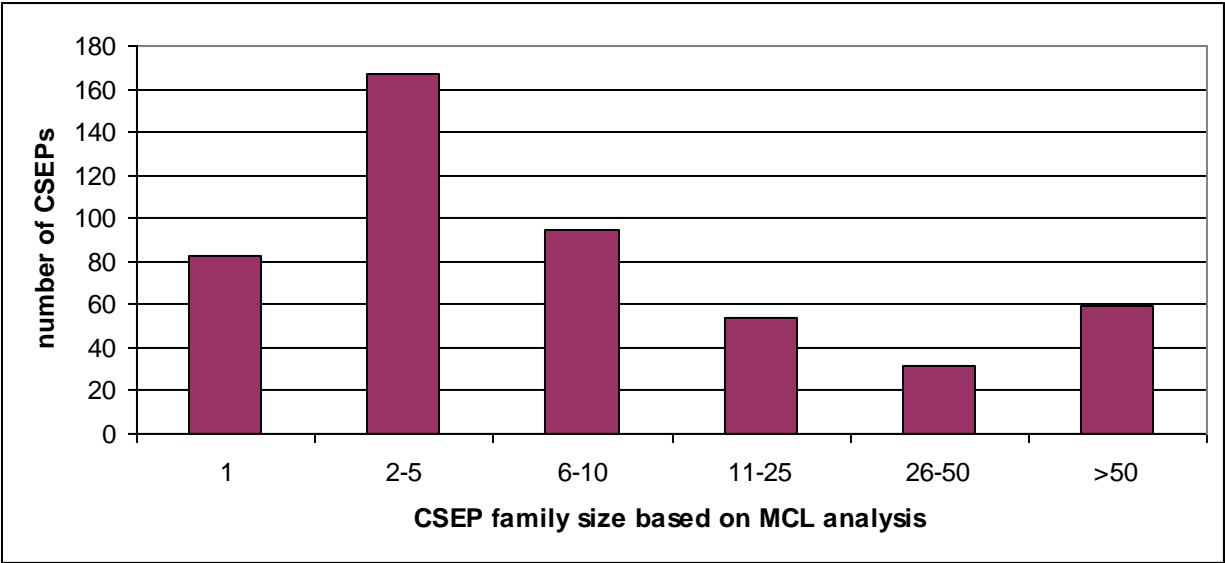

Additional File 2

Supplement: Additional file 2 — Size distribution histogram of MCL families. A: Number of families with a given family size. B: Number of CSEPs in families with a given family size. [file 1471-2164-13-694-S2.pdf]

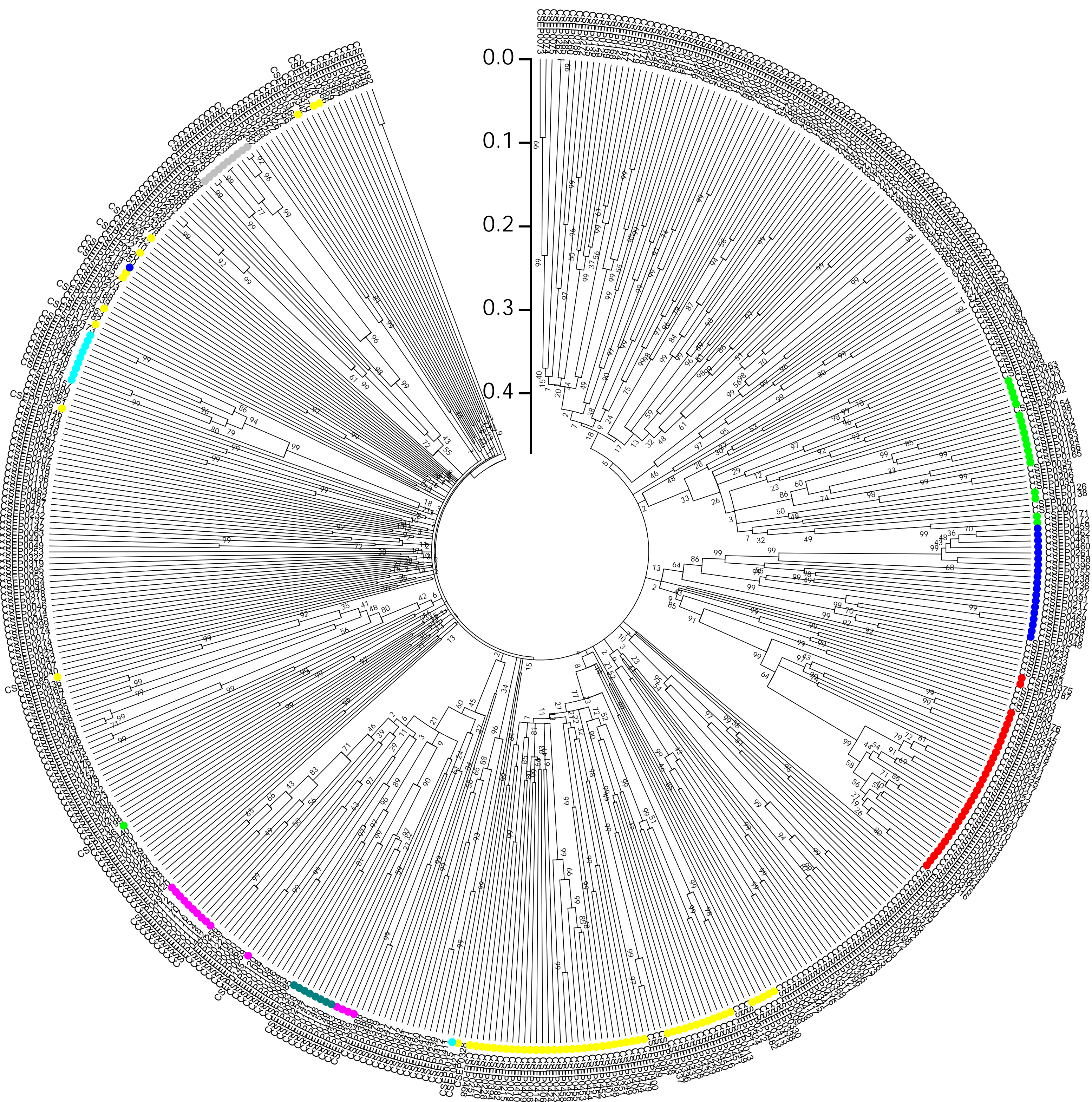

Supplement: Additional file 4 — CSEP bootstrap consensus tree showing CSEPs present in the eight largest MCL families visualized by colour codes. Yellow - Family 1; red - Family 2; blue - Family 3; green - Family 4; purple - Family 5; light blue - Family 6; grey - Family 7; green-blue - Family 8. Numbers at branches indicate bootstrap support on the basis of 100 replicates. The scale denotes the number of amino acid substitutions per site. [file 1471-2164-13-694-S4.pdf]

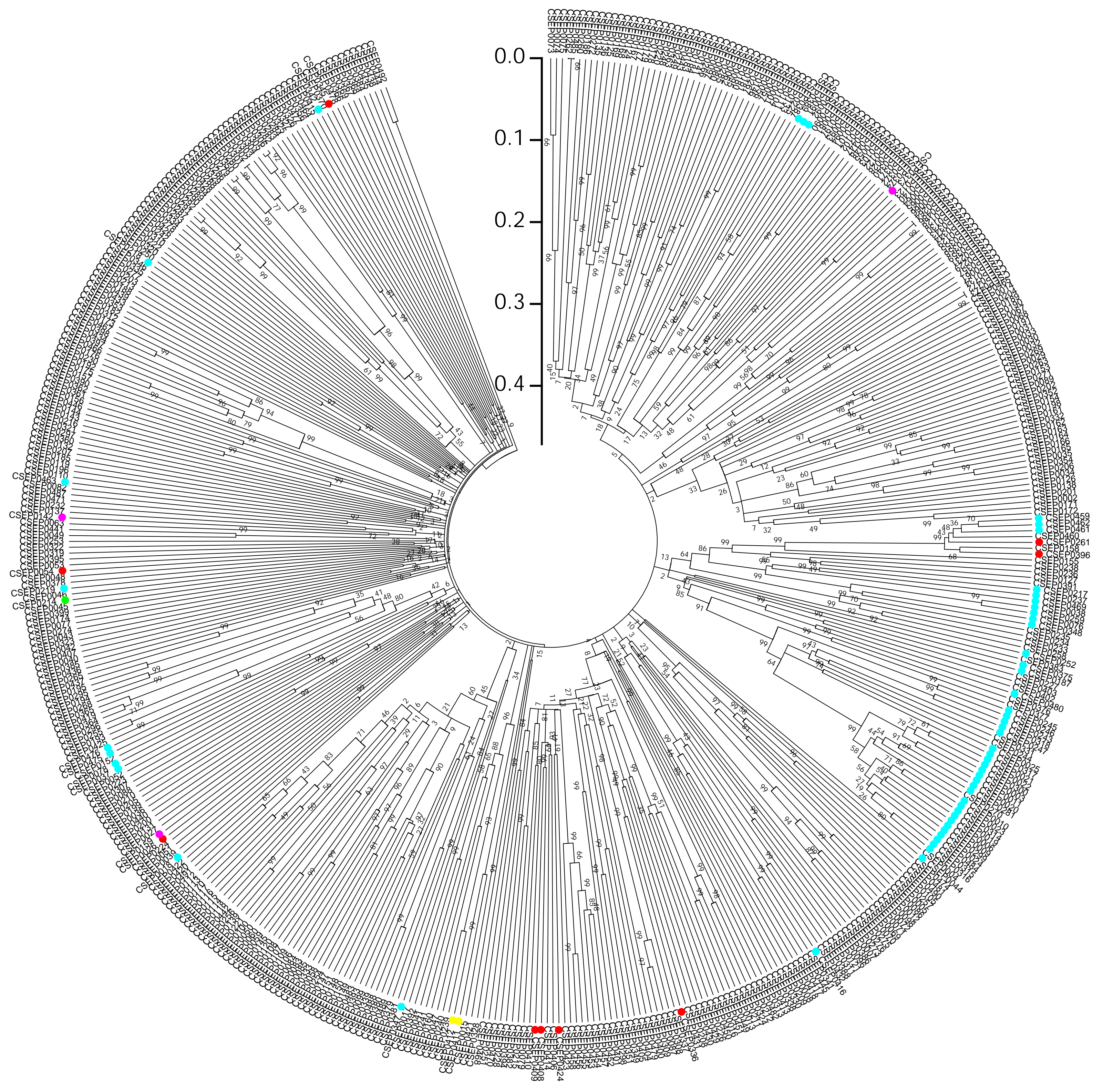

Supplement: Additional file 5 — CSEP bootstrap consensus tree showing CSEPs with Blast2Go hits. Light blue: Ribonucleases: red - coiled coil; yellow, pink and light green are other types of (uncharacterized) domains. Numbers at branches indicate bootstrap support on the basis of 100 replicates. The scale denotes the number of amino acid substitutions per site. [file 1471-2164-13-694-S5.pdf]

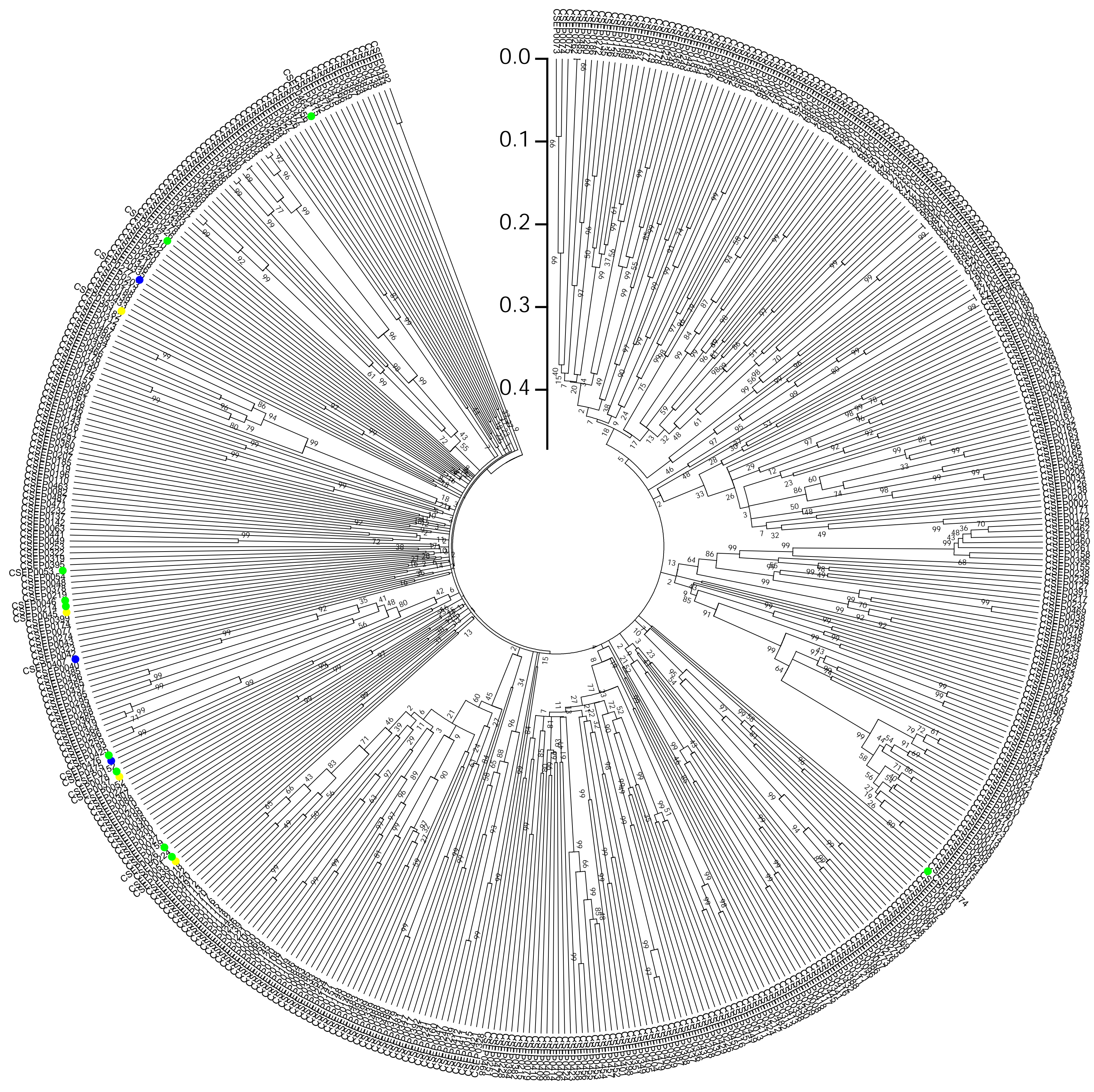

Supplement: Additional file 6 — CSEP bootstrap consensus tree showing CSEPs conserved in E. pisi and G. orontii . Highlighted are CSEPs with a recognizable hit (TBLASTN, e< 10-05) in the E. pisi and/or G. orontii genome. Colour code: blue - G. orontii, yellow - E. pisi, green - both G. orontii and E. pisi. Numbers at branches indicate bootstrap support on the basis of 100 replicates. The scale denotes the number of amino acid substitutions per site. [file 1471-2164-13-694-S6.pdf]

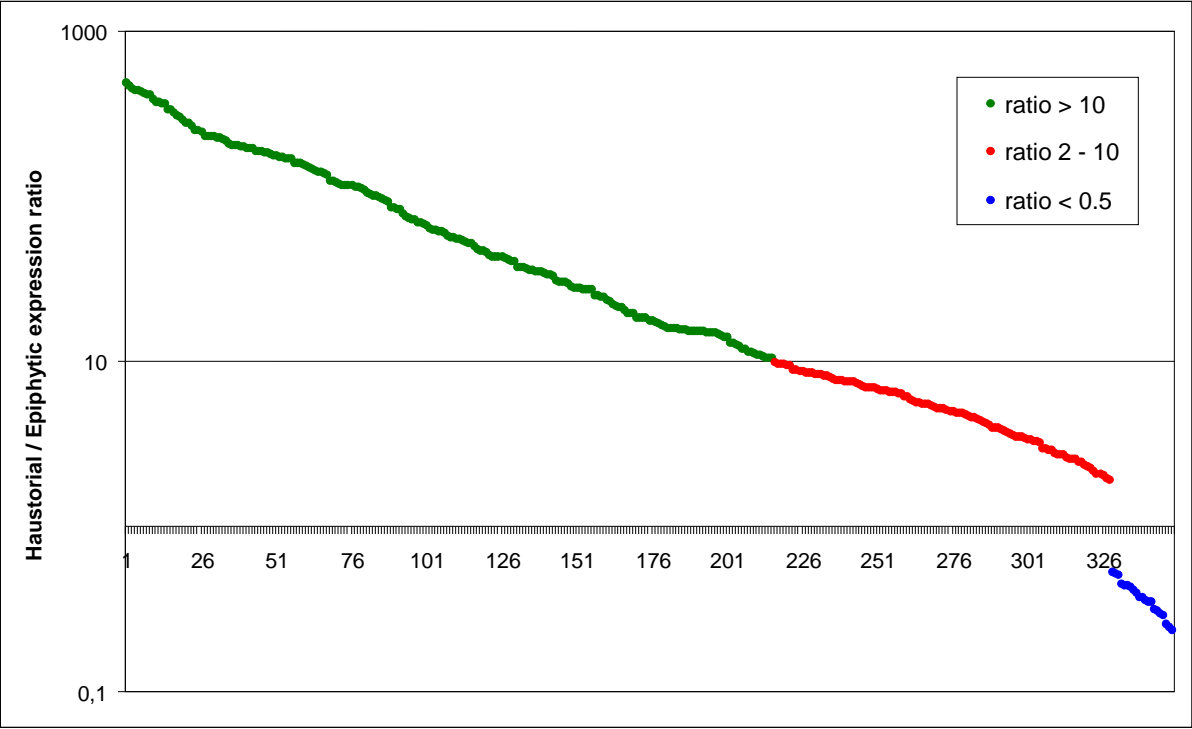

# Additional File 9

Supplement: Additional file 9 — CSEP expression plot. Plot of sorted haustorial versus epiphytic expression ratios of the 349 CSEPs with a ratio above 2 or below 0.5 and where the expression levels are high enough to calculate a reliable ratio. The plot shows that 216 CSEPs are expressed ≥10-times more in haustoria than in epiphytic tissues. The y-axis is log10-scaled. [file 1471-2164-13-694-S9.pdf]

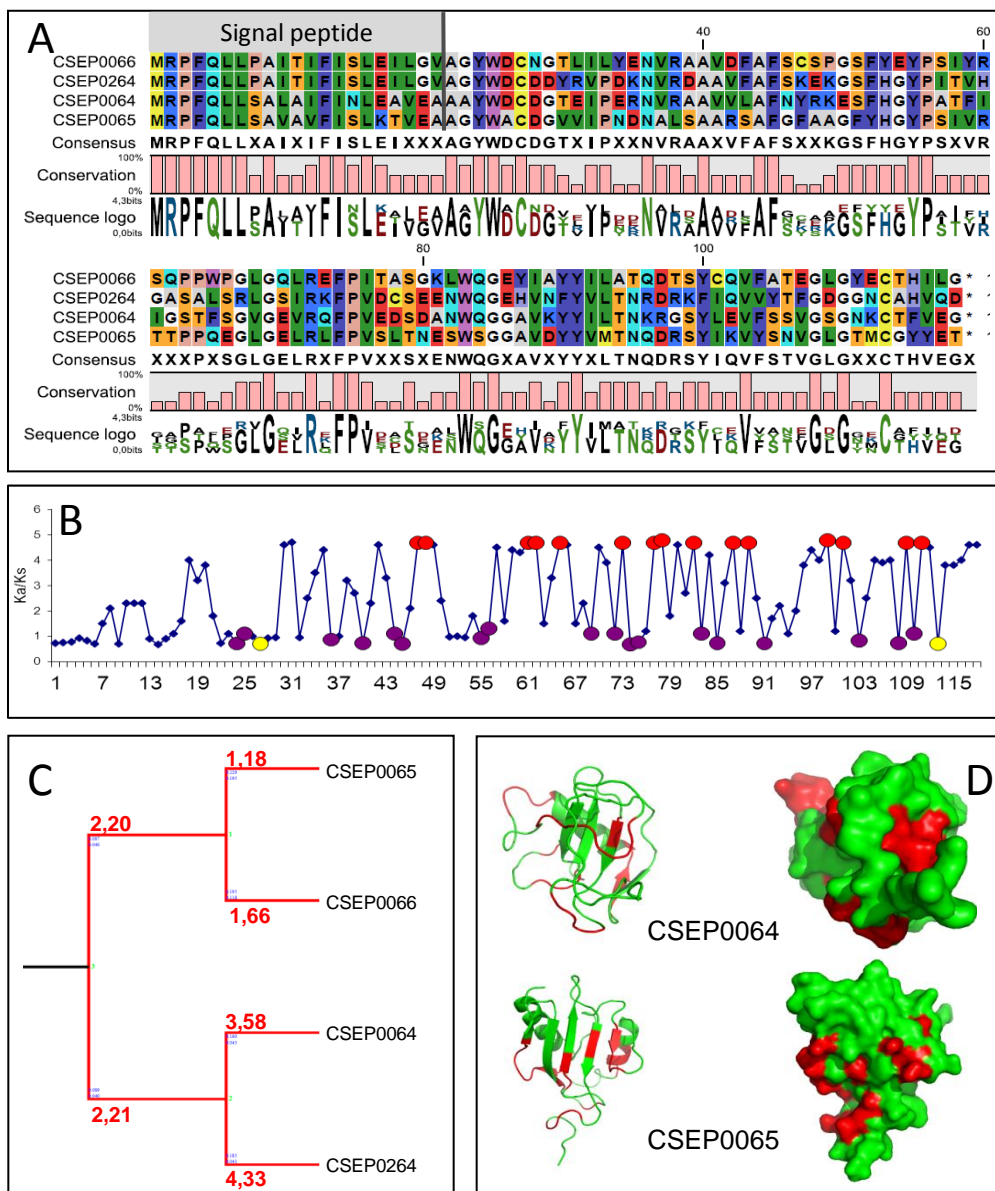

Additional File 11

Supplement: Additional file 11 — Protein structure and positive selection in CSEP family 21. A: Amino acid alignment of the seven members obtained with CLC main workbench (see Methods). B: Evidence for selection on the paralog members of family 21 was estimated using the Selecton server ([49,50]; http://selecton.tau.ac.il/). Codon sites under positive diversifying (red) or purifying (purple and yellow) selection and conserved cysteines (yellow) are indicated by coloured circles. C: Cladogram with Ka/Ks-values indicated for the individual branches calculated using the on-line server at http://services.cbu.uib.no/tools/kaks. D: 3D protein models of two family 21 members are shown and the amino acids under positive diversifying selection are highlighted in red. [file 1471-2164-13-694-S11.pdf]

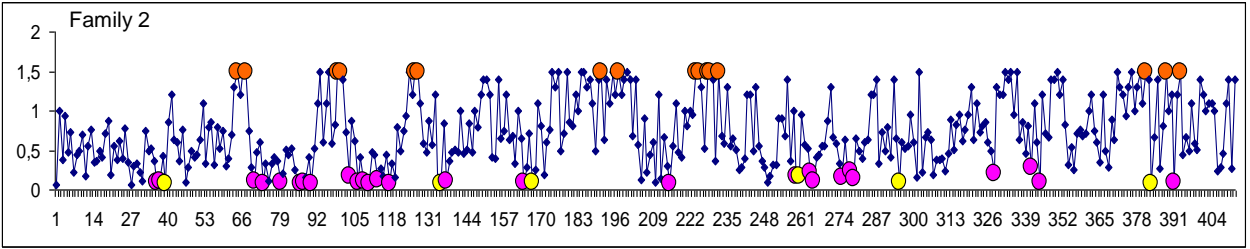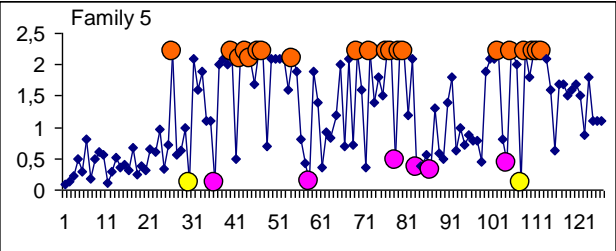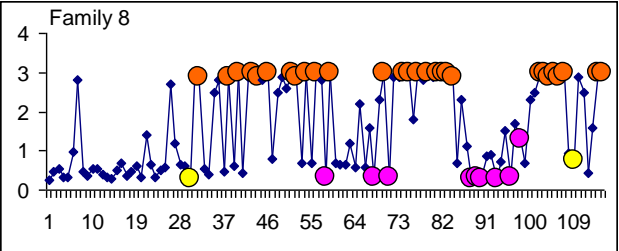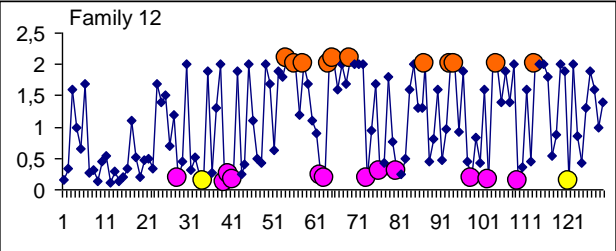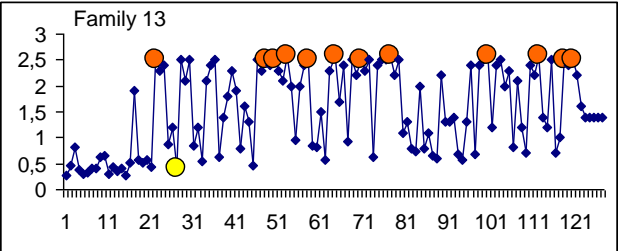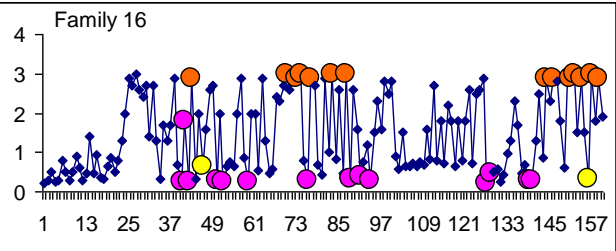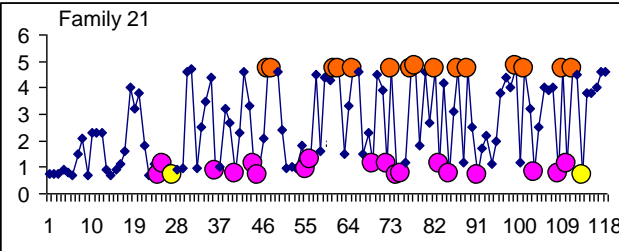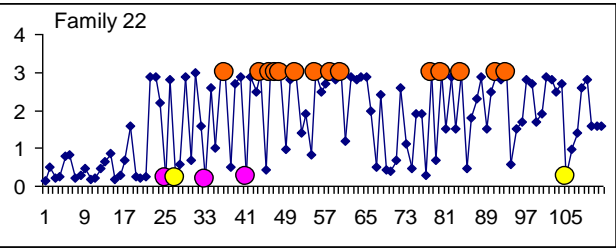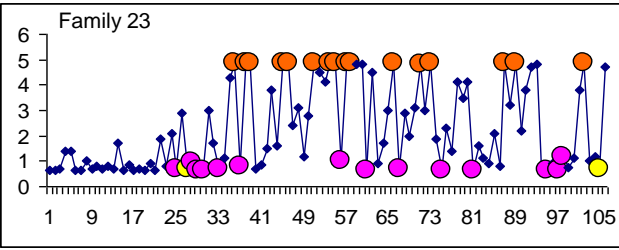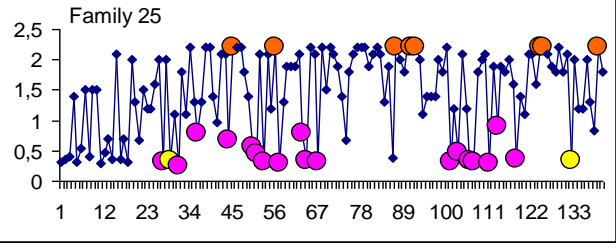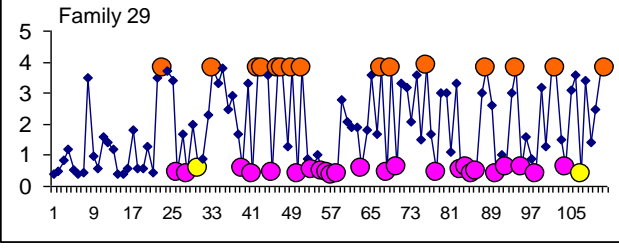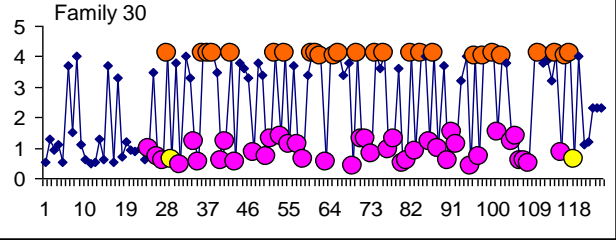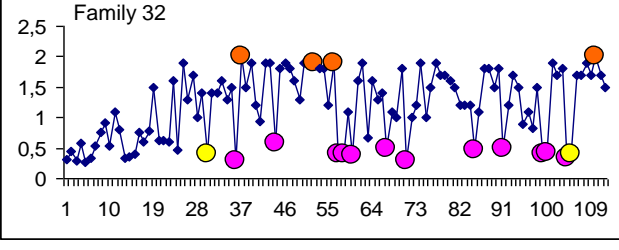

# Additional File 13

Supplement: Additional file 13 — Graphs of the distribution of codons under selection. Thirteen CSEP families with amino acid sites under positive selection (orange) are represented. The most conserved positions are shown in pink with the conserved cysteines in yellow. The y-axis is the Ka/Ks-value and the x-axis is the position in the protein including the signal peptide, which is mainly under purifying selection. The Ka/Ks-values were calculated using the Selecton server ( [49], http://selecton.tau.ac.il/). [file 1471-2164-13-694-S13.pdf]

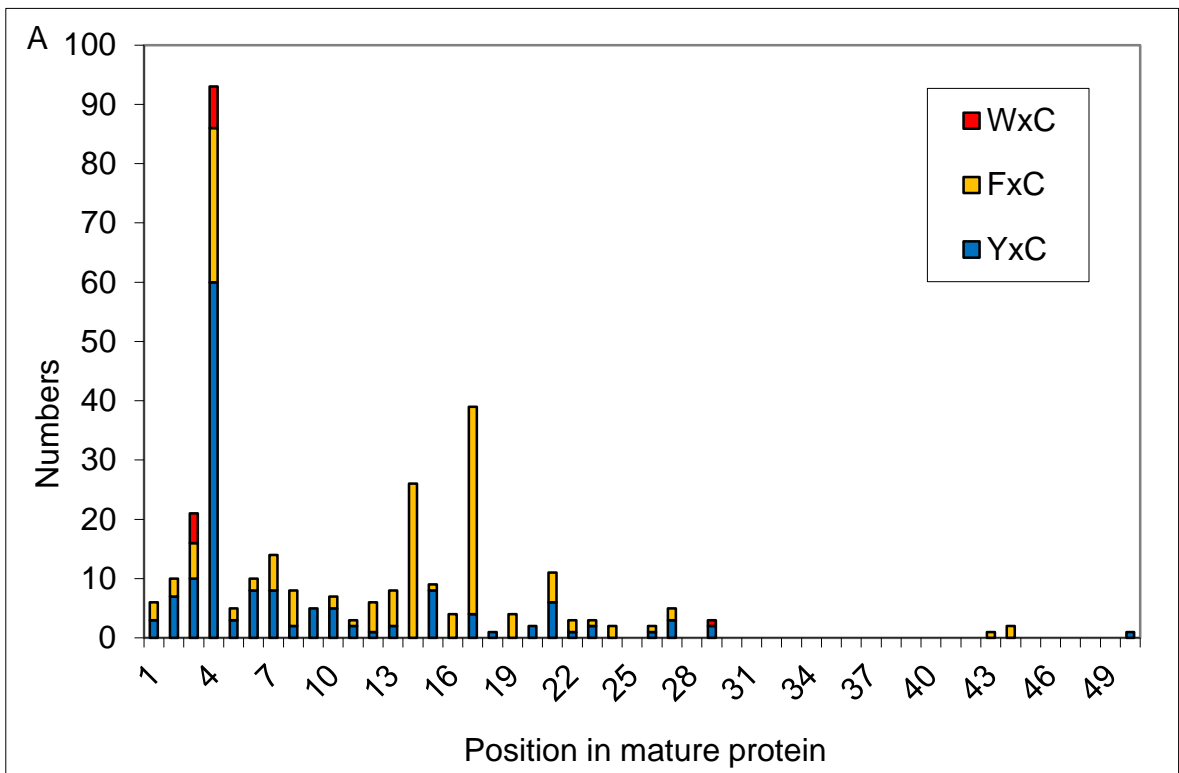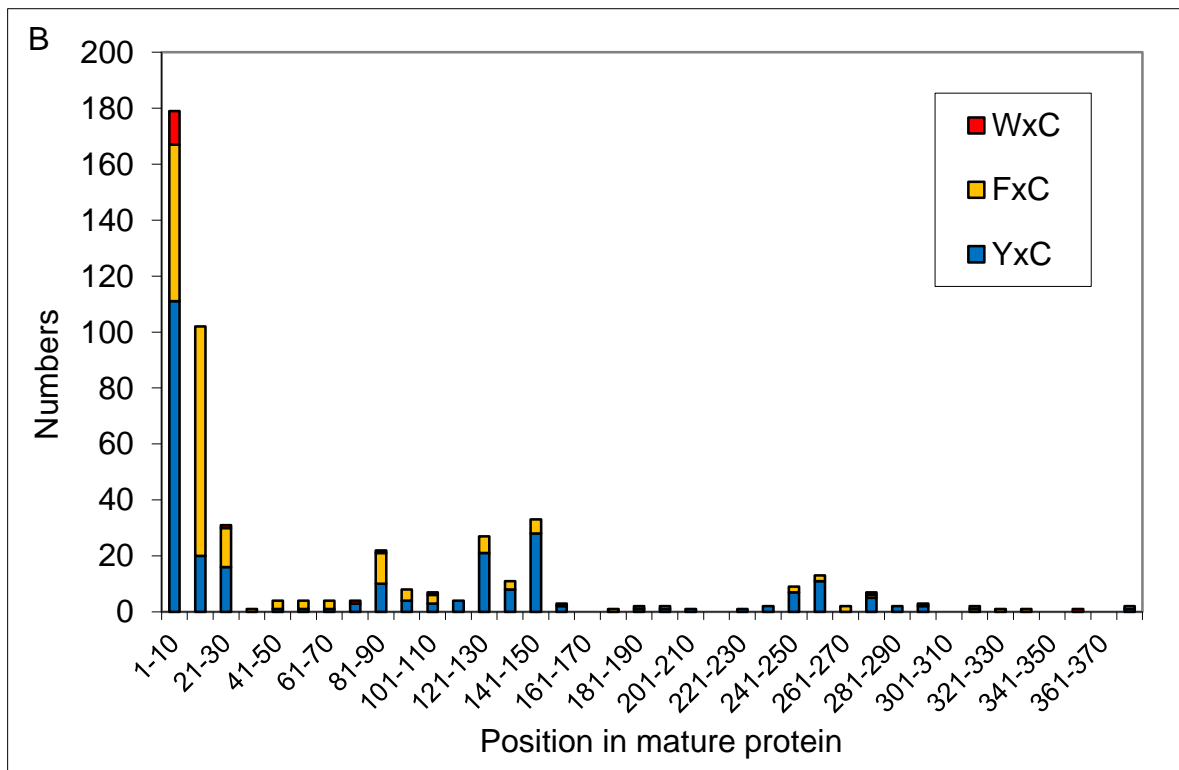

## Additional File 21

Supplement: Additional file 21 — Distribution of the YxC motifs. A: Distribution of the YxC motifs among the 307 CSEPs having this motif within the first 50 amino acids. The cumulative number of the YxC, WxC and FxC versions of the YxC-motif is plotted versus the distance of the first amino acid of the motif from the signal peptide cleavage site. B: Distribution of the YxC motifs among the 352 CSEPs having one or more versions of this motif. The cumulative number of the YxC, WxC and FxC versions of the YxC-motif is plotted versus the distance of the first amino acid of the motif from the signal peptide cleavage site. [file 1471-2164-13-694-S21.pdf]

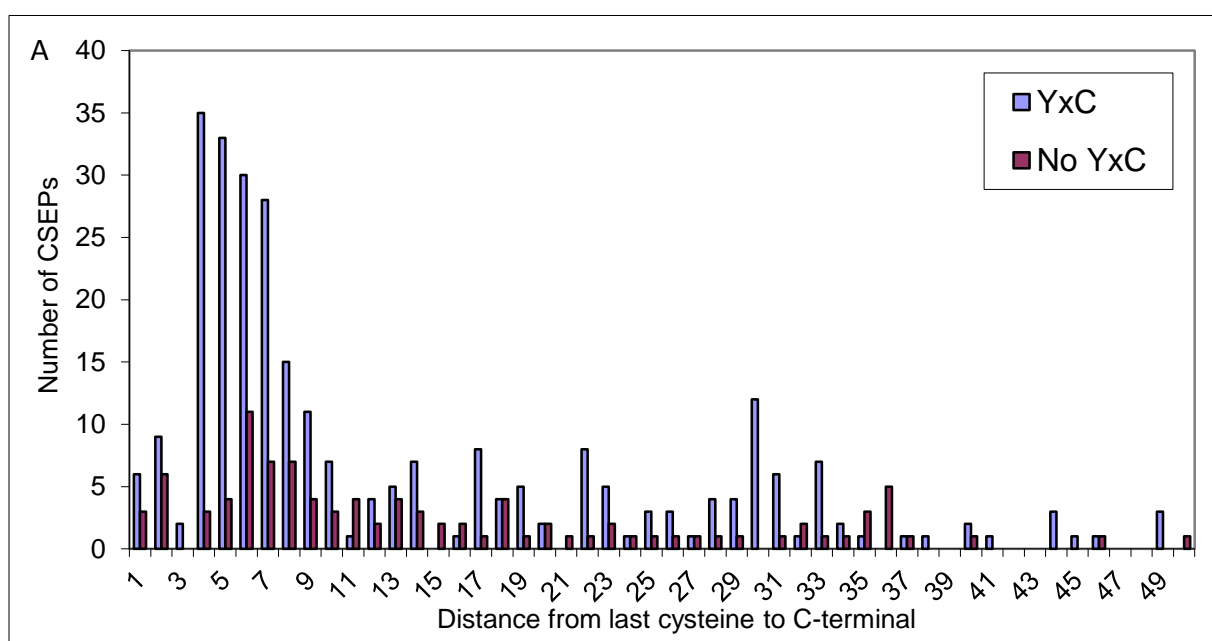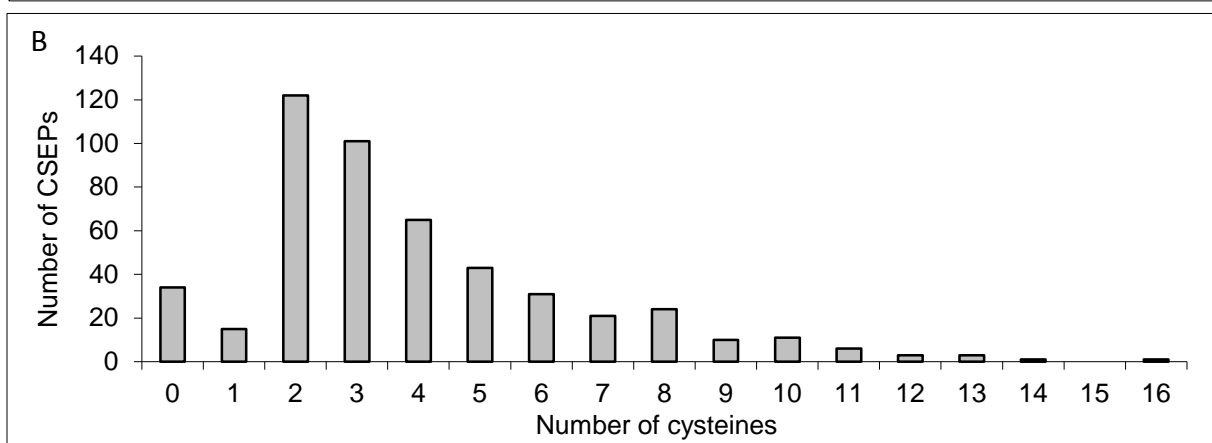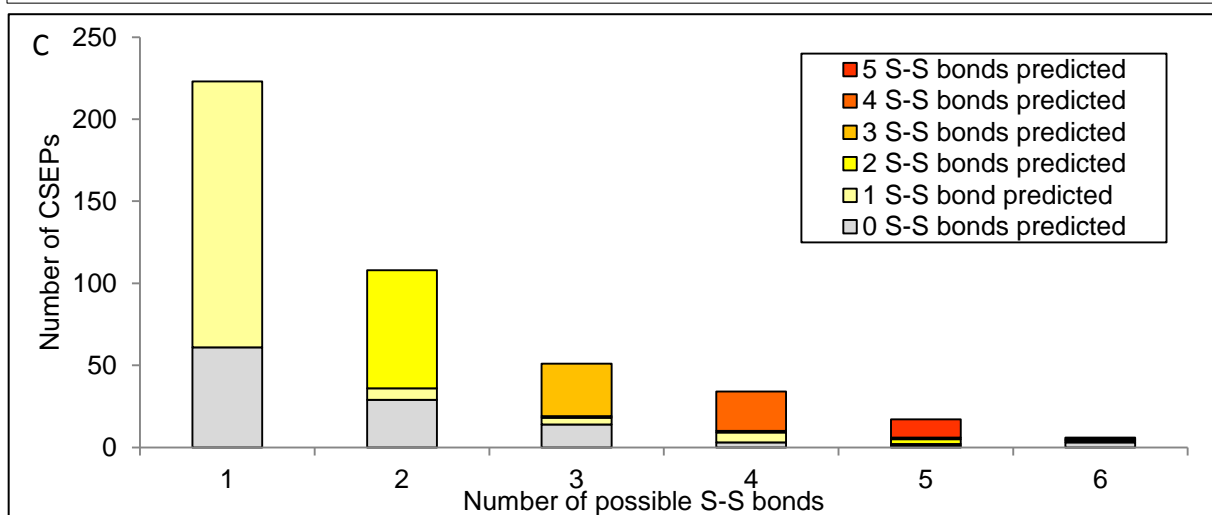

Supplement: Additional file 22 — Cysteines and prediction of disulphide bonds in CSEPs. A: The histogram shows the number of CSEPs versus the position of the last cysteine from the C-terminus of the protein. B: Distribution of CSEPs containing 0 – 16 cysteines. C: The histogram shows the prediction of disulfide bonds in the CSEPs using Disulfind [12]. [file 1471-2164-13-694-S22.pdf]

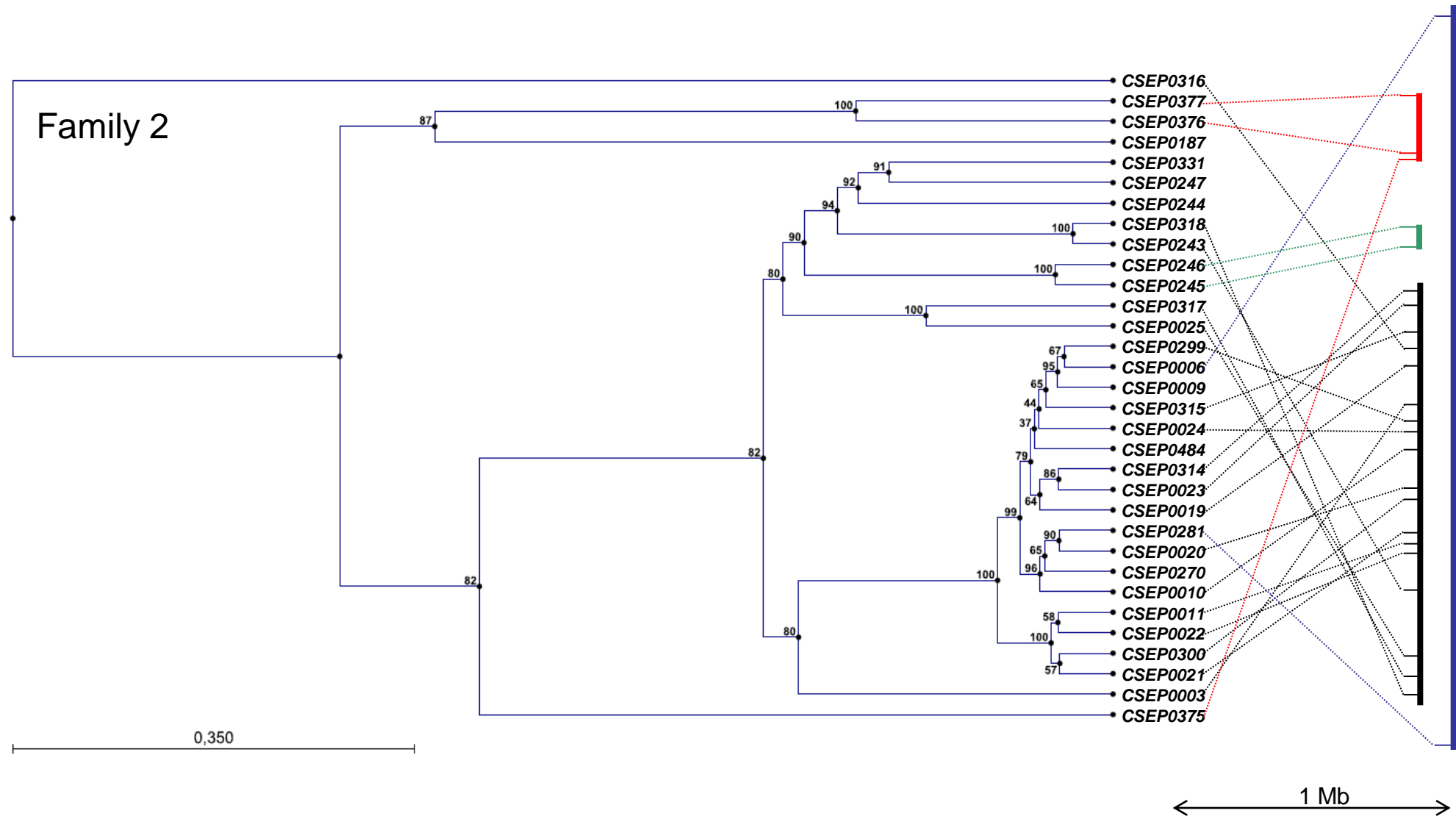

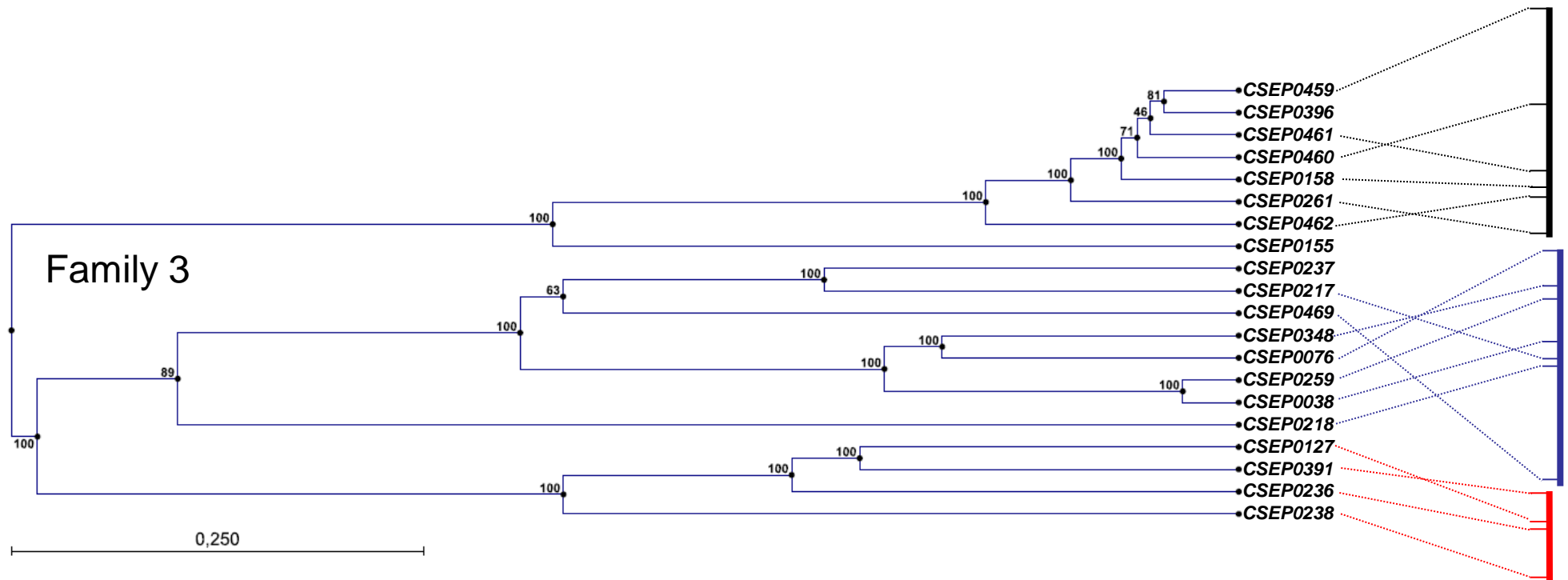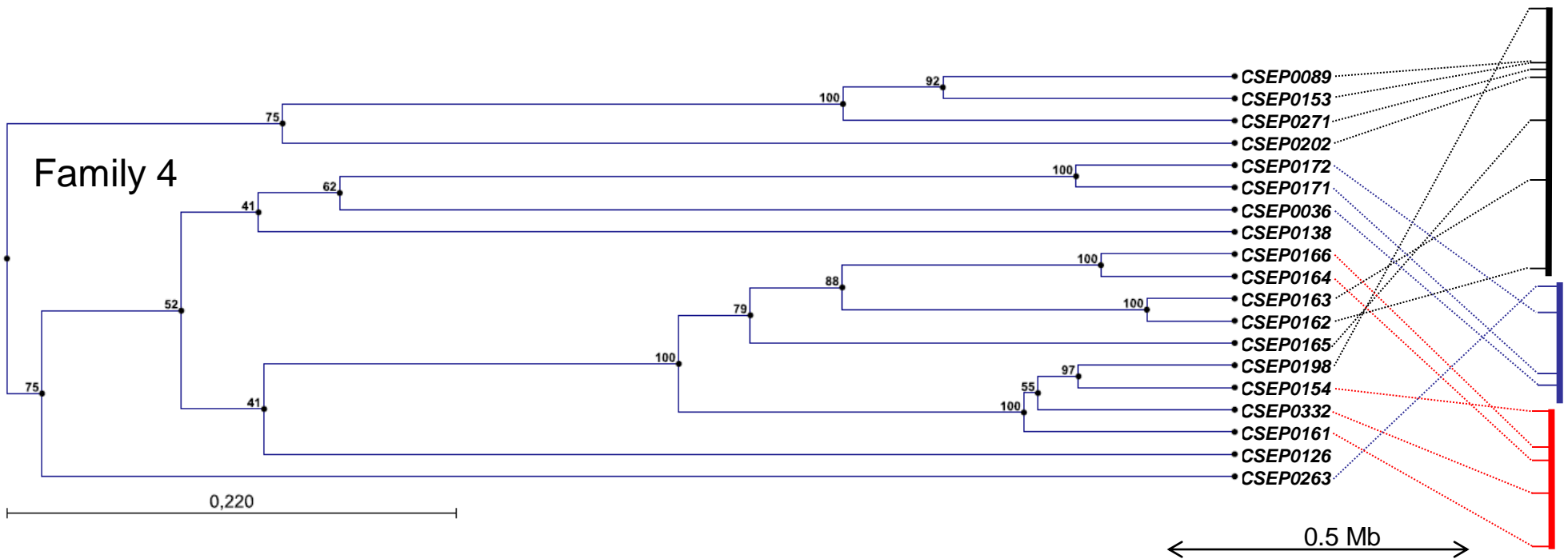

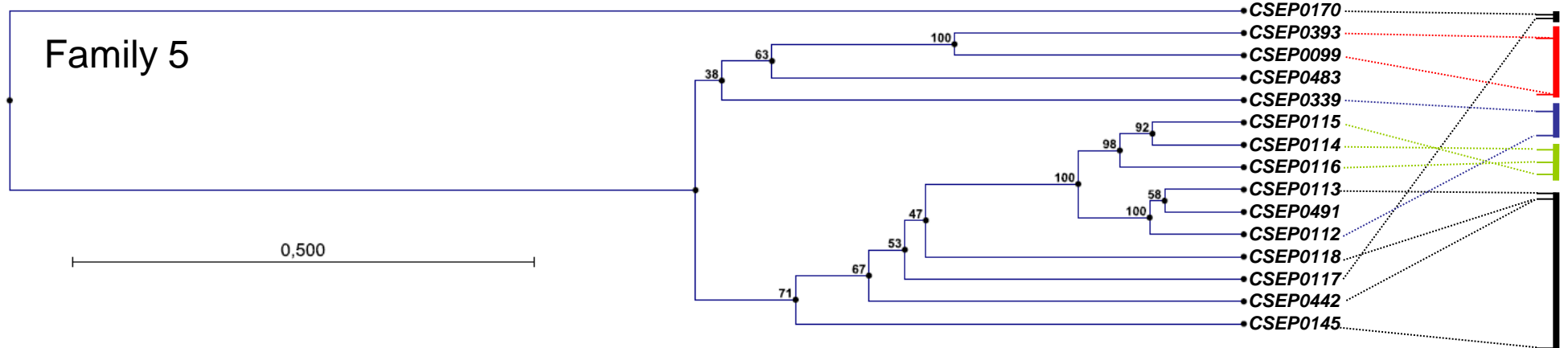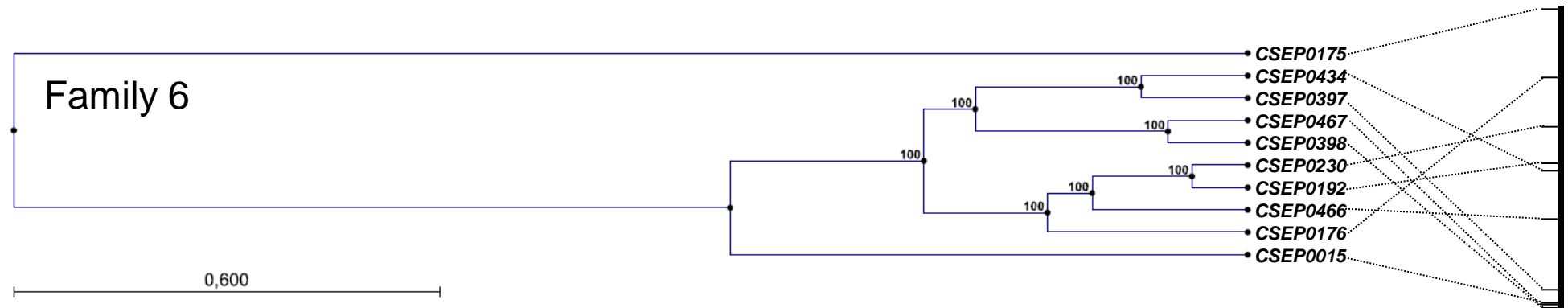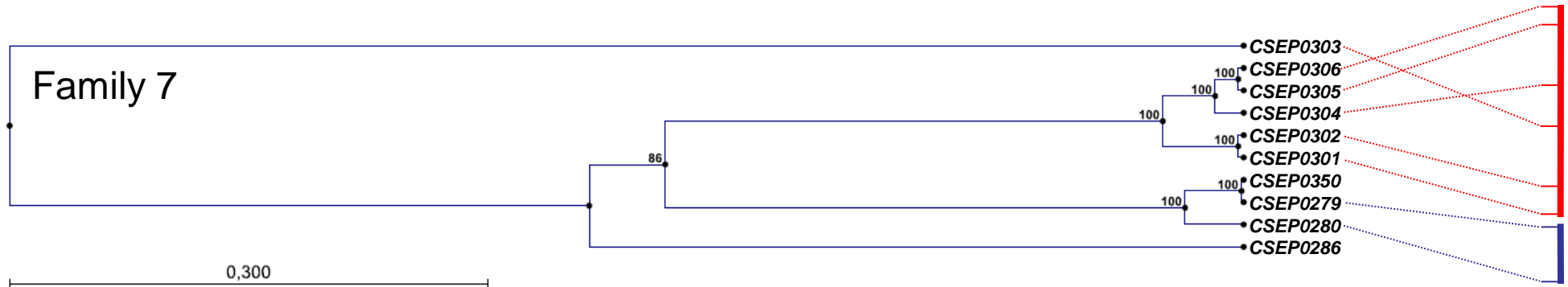

0.5 Mb

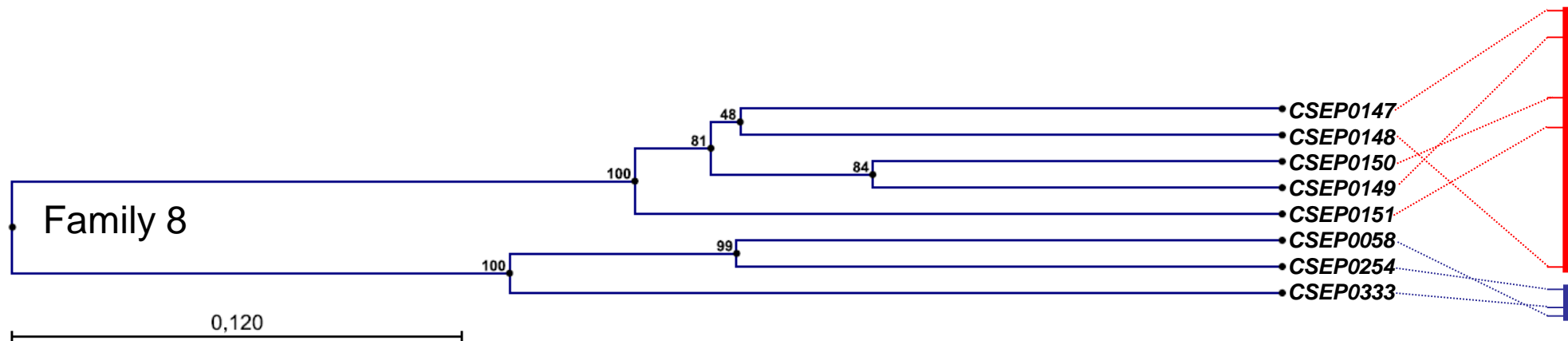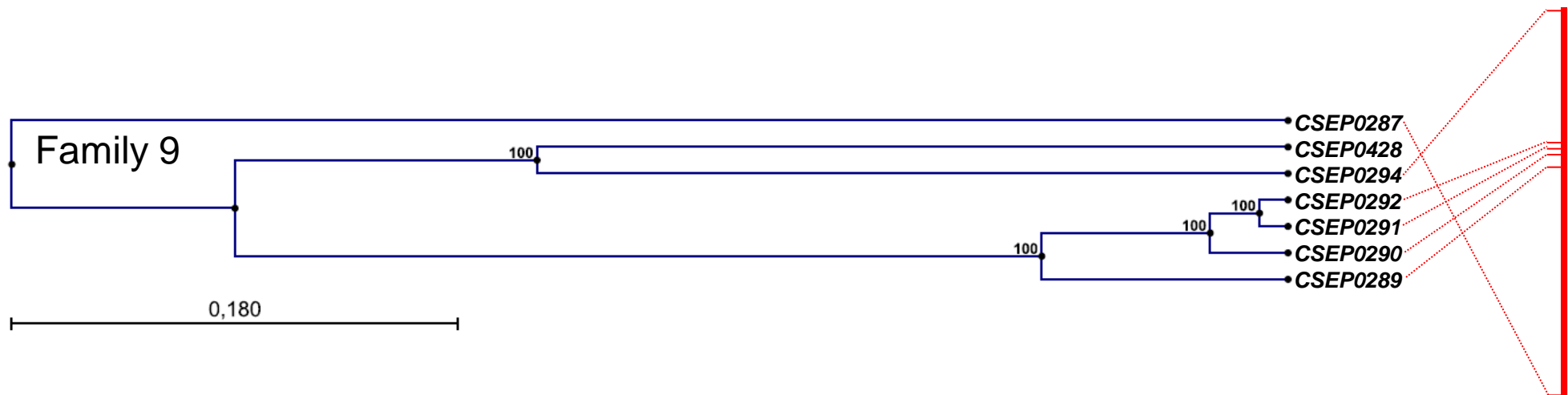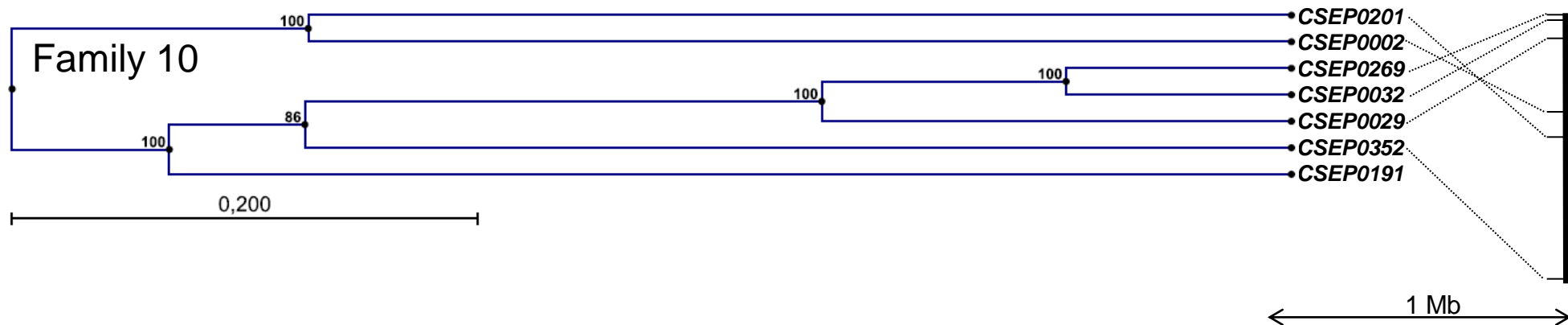

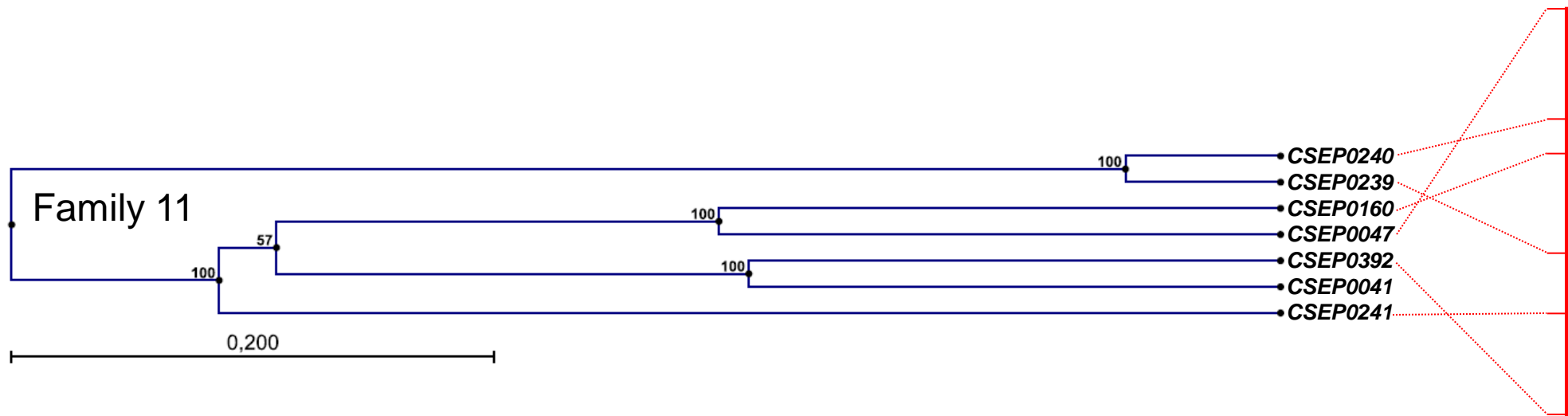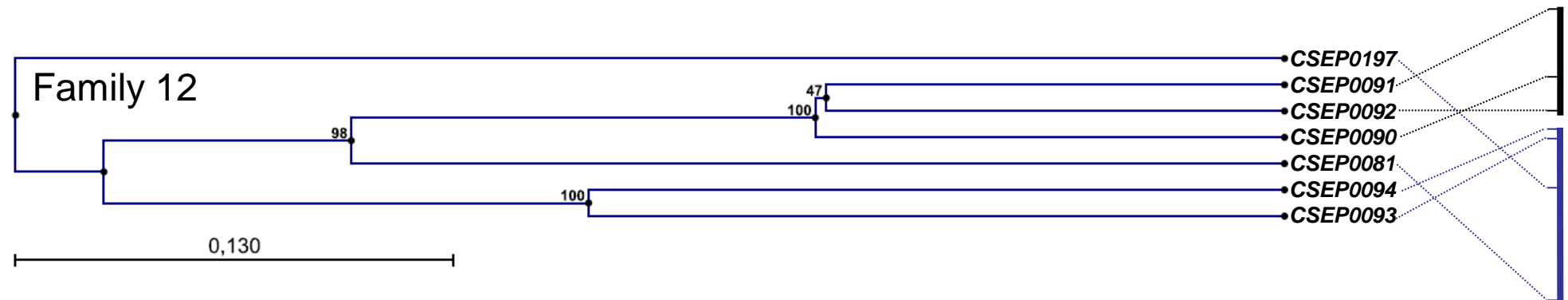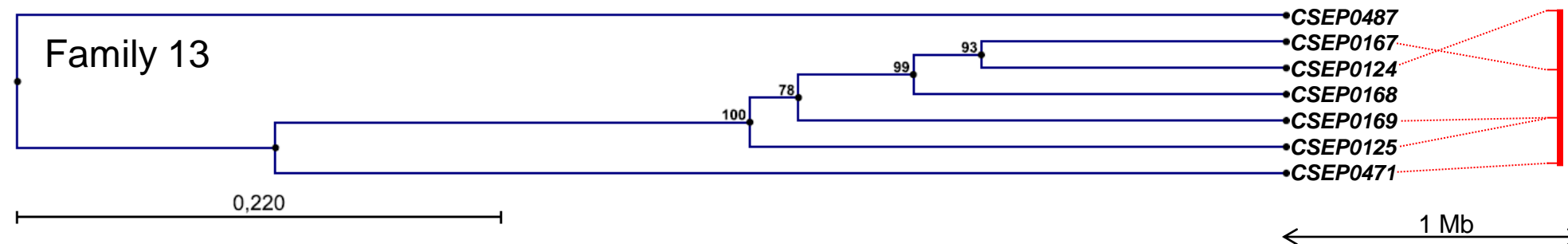

1 Mb

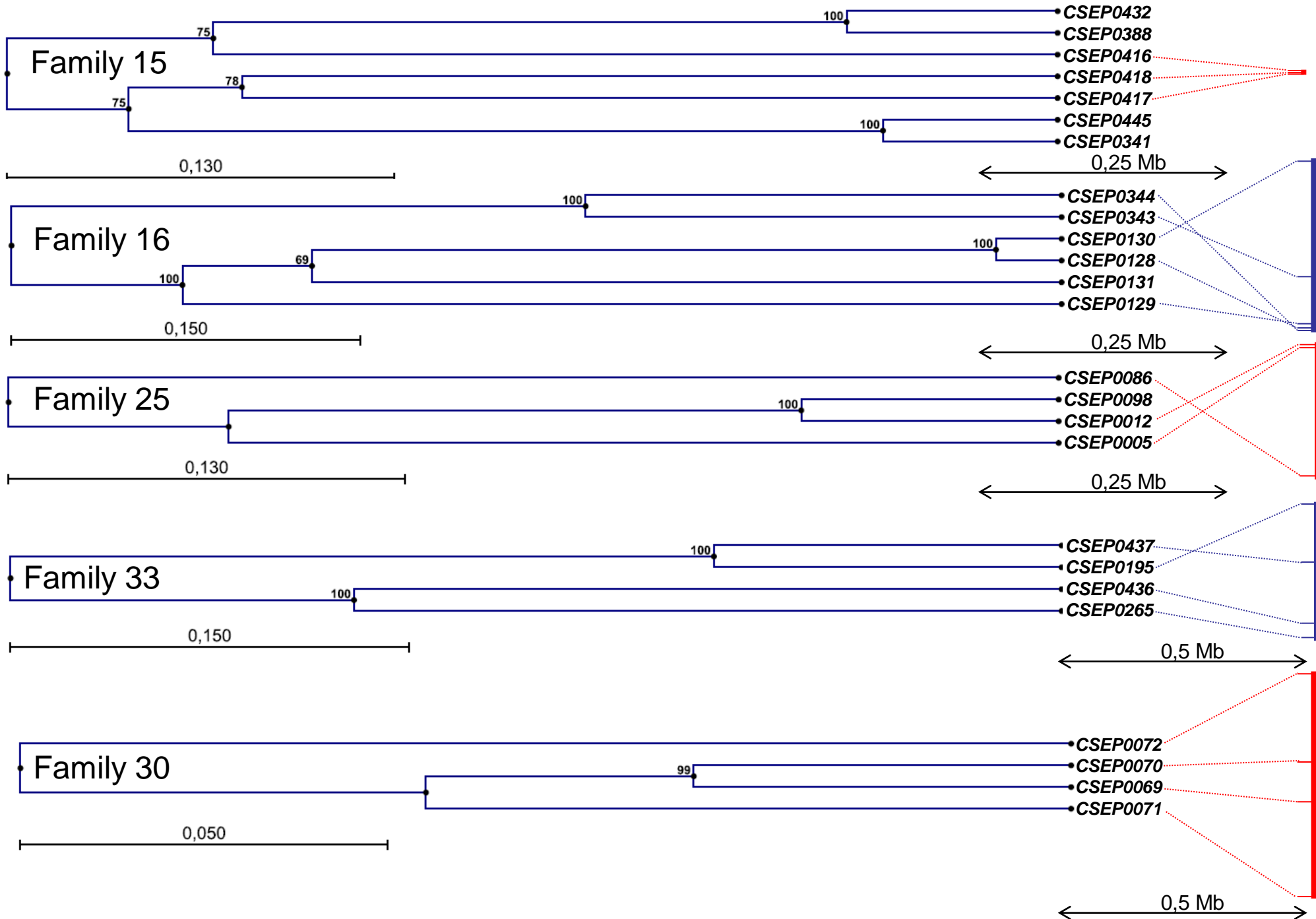

Supplement: Additional file 24 — The relationship between CSEP clustering on genome sequence scaffolds and their sequence homology. The Figure shows families 2–13, 15, 16, 25, 30 and 33. The scaffolds are drawn as vertical, solid bars (colours indicate separate contigs) with a scale bar in the right bottom corner. The phylogenetic tree is based on nucleotide sequences and calculated using the UPGMA algorithm with CLC Main Workbench. Bootstrap values on the basis of 100 replicates are shown at the nodes, the scale bar at the left bottom corner indicates the number of nucleotide substitutions per site. The CSEPs not connected to any scaffold with a dotted line are not found to be clustered. [file 1471-2164-13-694-S24.pdf]

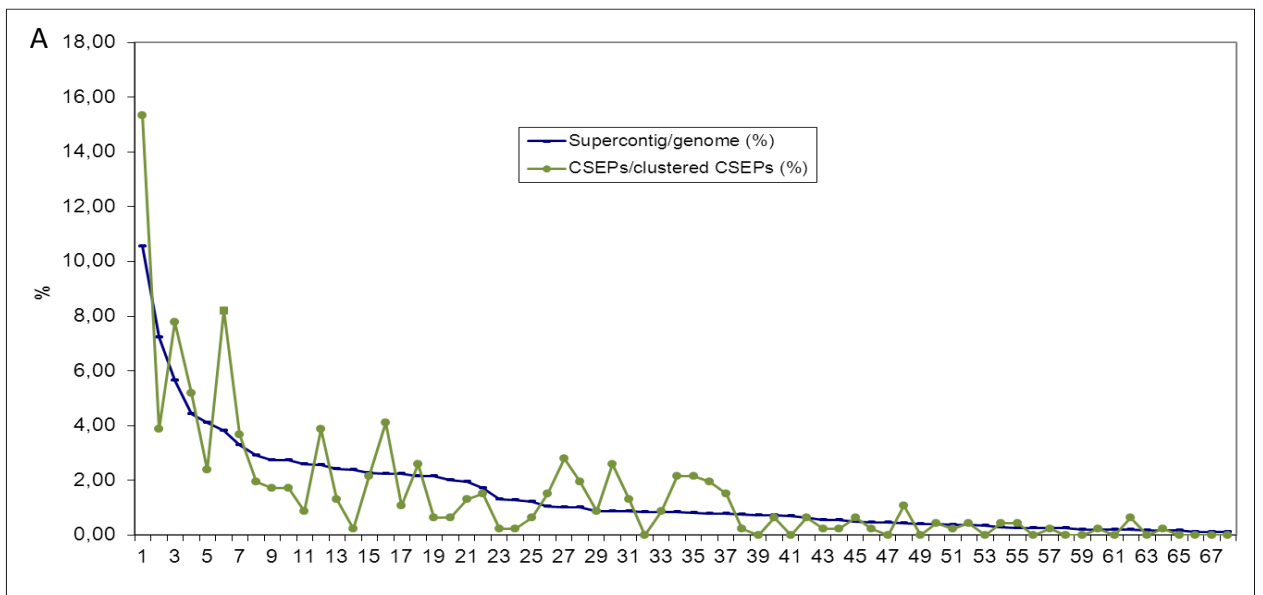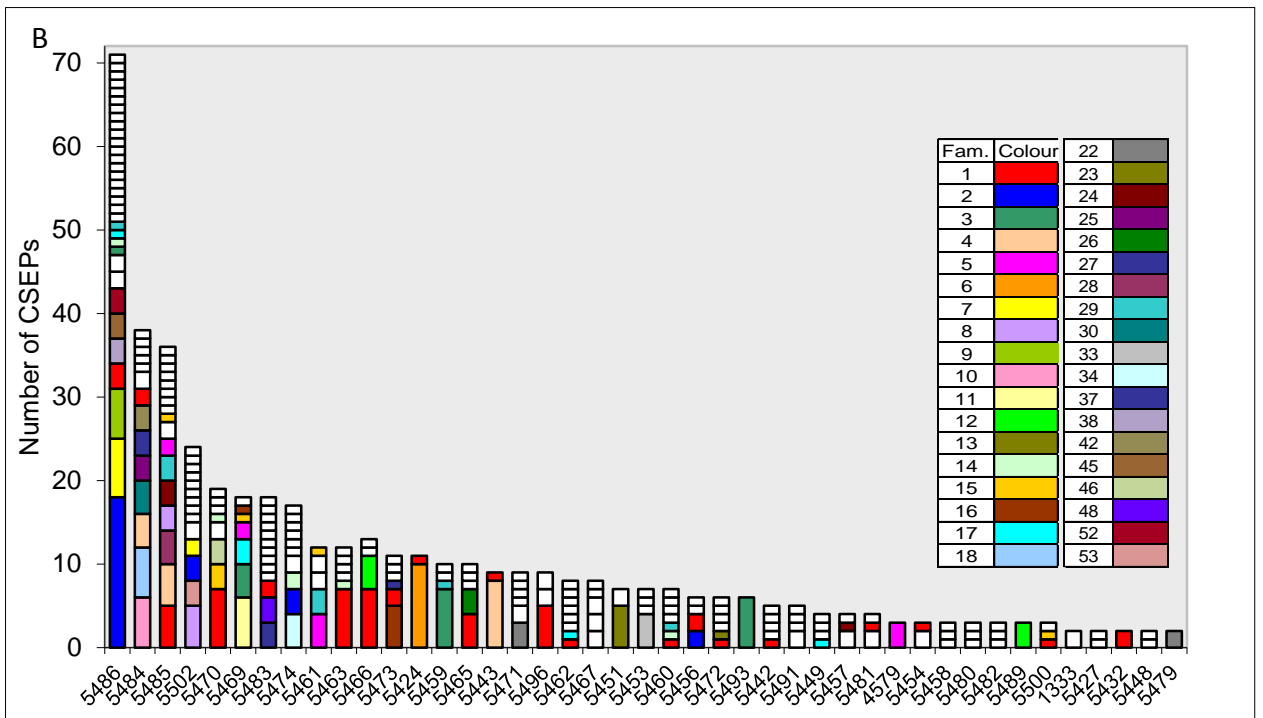

**Additional File 25**

Supplement: Additional file 25 — Clustering of CSEP genes. A: The 68 genomic sequence scaffolds of more than 100 kb are expressed in % of their sum (92 Mb, blue line) and ordered according to their length. The 463 CSEPs found on each scaffold of more than 100 kb are expressed in % of their total number (green line). B: The family-wise distribution of 455 CSEPs on the 43 scaffolds harboring at least two CSEPs. Families with at least three clustered members are colour-coded so that the coloured histograms show the number of clustered members from each family on each scaffold. [file 1471-2164-13-694-S25.pdf]

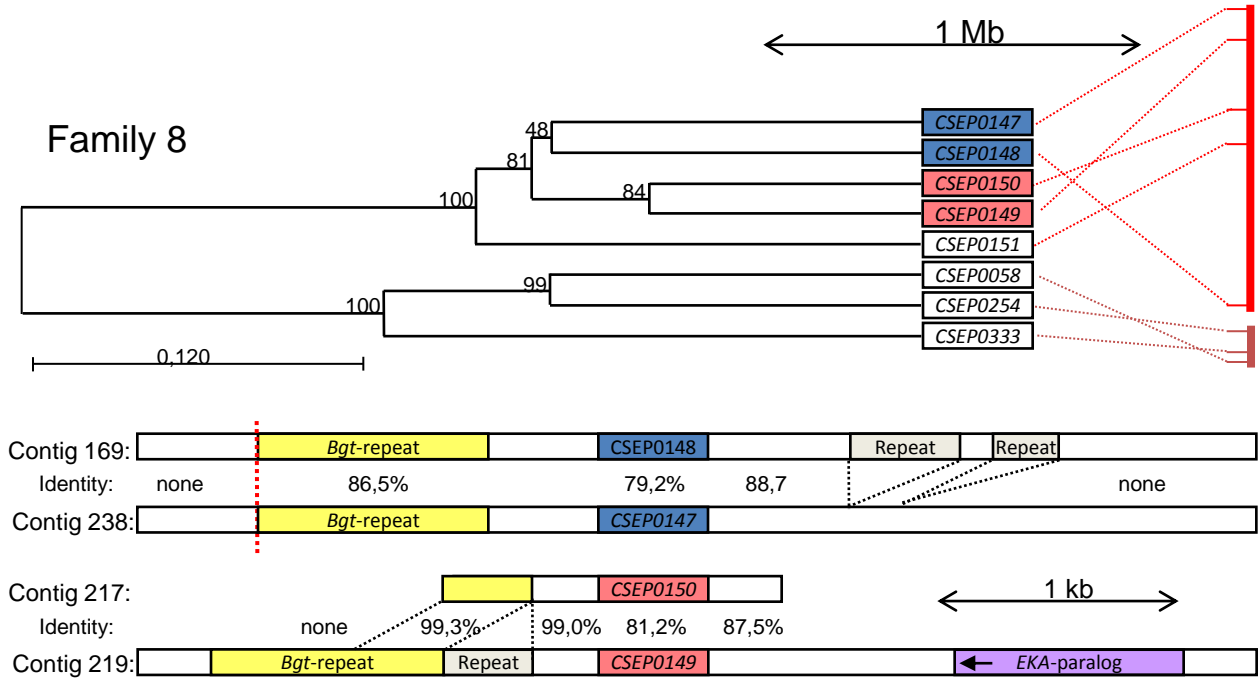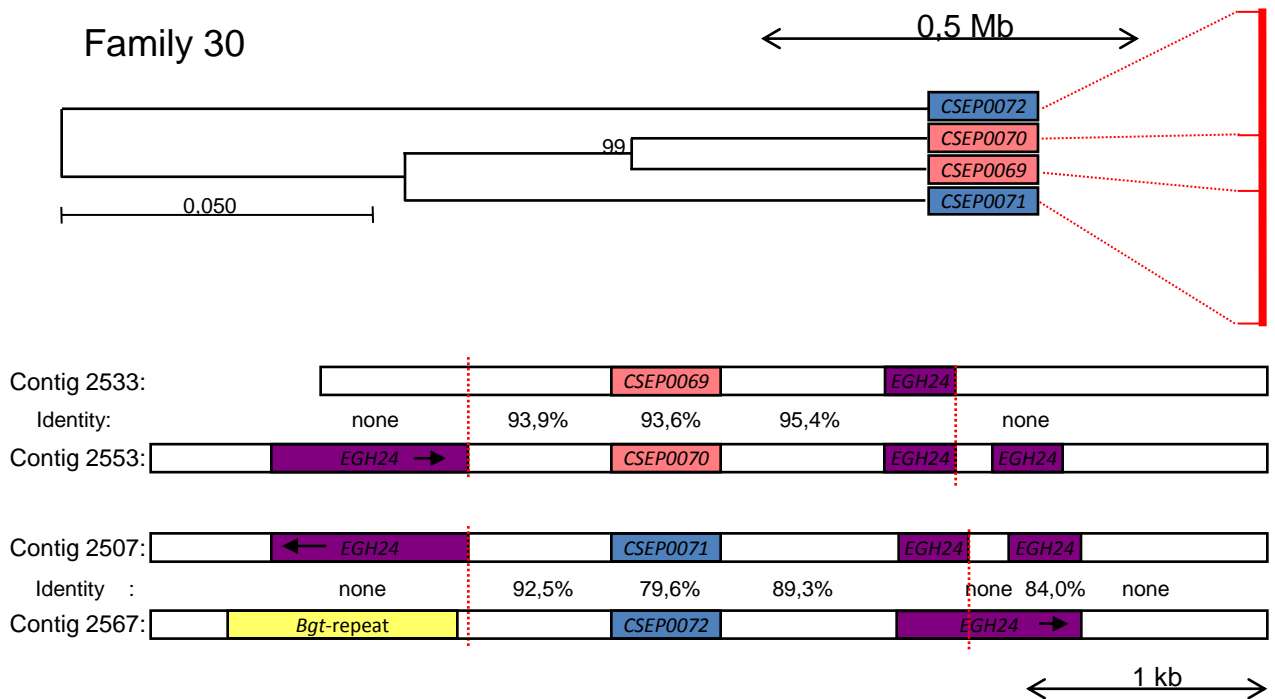

**Additional File 26**

Supplement: Additional file 26 — Clustering of selected CSEP family members. Genome clustering of four CSEP paralogs from family 8 and four CSEP paralogs from family 30 on their respective sequence scaffolds. The schematic illustration of the genome organizations with repetitive elements is shown below each dendrogram with indications of the sequence homologies in pair-wise comparisons (note that the colour coding in the dendrogram matches the colour coding in the scaffolds). The element Egh24 is a SINE [15], the Bgt repeat is an un-characterized repeat (GenBank AJ002007.1) from B. graminis f.sp. tritici, the EKA paralog is an AvrA10/K1-paralog [32] . Vertical dotted red lines indicate abrupt breaks in sequence homology. The scale bars next to the dendrograms refer to the genomic scaffolds. [file 1471-2164-13-694-S26.pdf]
