# Supplementary material for: Structure and evolution of barley powdery mildew effector candidates
Source: BMC Genomics. 2012 Dec 11;13:694. doi: 10.1186/1471-2164-13-694 (PMC3582587; doi:10.1186/1471-2164-13-694)
Supplement: Additional file 3 — Analysis of selection on CSEPs. The table shows the full data set from the analyses of positive and purifying selection for all 72 CSEP families. Footnotes: 1) Indicates whether the family has the YxC-motif in the N-terminus of the mature protein. The symbol ½ indicates that some members have and others do not have the motif. 2) The presence of a cysteine close to the C-terminus and the distance to the C-terminus 3) Conserved cysteines are in the mature protein. In some cases there are a few members which are truncated and therefore lacking the terminal cysteine, but in the table it is counted anyway 4) Length of proteins: The average lengths of the proteins were calculated for each family. If the average length was below 150 amino acids, it was coloured light green, if the average length was more than 300 it was coloured grey 5) Gene expression ratio in haustorial samples versus epiphytic samples and calculated as averages for each family. Colour codes: Orange: >100x, yellow: 50-100x, light yellow: 10-50x 6) Percentages of CSEPs in each family found only in haustoria samples by proteome analysis 7) Codon-based test of positive and purifying selection. The two left columns show the numbers of pairs with significant positive selection (z-tests at 5% level) compared to the total number of pairs within each family. The two right columns show the values of P less than 0.05 that are considered significant at the 5% level (modified Nei-Gojobori (assumed transition/transversion bias = 1)). The test statistic (dN - dS) and (dS - dN) are shown for positive and purifying selection respectively. dS and dN are the numbers of synonymous and nonsynonymous substitutions per site, respectively. 8) Codon-based calculations of positive and purifying selection using the Selecton-server and based on a Bayesian inference approach [49]. The left column indicate the number of codons under positive or purifying selection. The middle column shows the significant levels of model M8a versus m [file 1471-2164-13-694-S3.pdf]

Additional file 3

Families of paralogs		YxC motif and cysteines			Length	RNA-exp.	Protein-exp.	Modified Nei-Gojobori-method for selection[7]				Selecton-server results[8]			Ka/Ks based on Liberles (2001)[9]	
Family name	Number of members	Motif[1]	C-term. cysteine[2]	Conserved cysteines[3]	Average peptide length[4]	Haustoria/ Epiphytic Exp-ratio[5]	Preference for haustoria expression[6]	Z-test pair-wise Positive	Z-test pair-wise Purifying	Z-test overall Positive P-value/(dN-dS)	Z-test overall Purifying P-value/(dS-dN)	Codons under positive selection	M8 versus M8a-model test P-values	Average Ka/Ks-value	Branches with Ka/Ks>1 / total branches	Average Ka/Ks if Ka/Ks>1
1	59		no	3	326	15	5	18 / 1711	657 / 1711	1,000 / -2,903	0,001 / 3,059	21 / 397	0,01	0,68	8 / 116	1,38
2	32	FxC	no	4	396	6	0	3 / 496	249 / 496	1,000 / -3,987	0,000 / 5,644	14 / 412	0,001	0,73	15 / 62	1,15
3	20	F/YxC	no	10	384	6	0	0 / 190	133 / 190	1,000 / -6,200	0,000 / 6,125	5 / 411	0,001	0,70	5 / 38	1,23
4	19	FxC	yes (4-14)	2	153	58	16	8 / 171	29 / 171	1,000 / -1,626	0,058 / 1,584	18 / 163	0,001	1,20	10 / 36	1,56
5	15	F/Y/(H)xC	no	2	107	94	13	9 / 105	17 / 105	1,000 / -1,069	0,121 / 1,177	20 / 125		1,41	9 / 28	2,07
6	10	YxC	yes (1-9)	4	311	21	50	1 / 45	25 / 45	1,000 / -3,216	0,000 / 3,211	45 / 427	0,001	1,04	0 / 18	
7	10		no	none	131	3	0	1 / 45	23 / 45	1,000 / -3,150	0,000 / 3,309	none	no test	0,62	4 / 18	2,67
8	8	YxC	yes (4)	2	118	25	0	11 / 28	0 / 28	0,034 / 1,845	1,000 / -1,608	28 / 115	0,001	1,80	11 / 14	1,72
9	8		no	none	178	16	0	0 / 21	14 / 21	1,000 / -4,978	0,000 / 5,314	none	no test	0,52	0 / 12	
10	7	FxC	no	2	160	37	57	1 / 21	5 / 21	1,000 / -0,957	0,189 / 0,883	5 / 161		1,31	5 / 12	1,24
11	7		no	2	164	0	0	0 / 21	12 / 21	1,000 / -3,529	0,0001 / 3,205	3 / 171	no test	0,92	2 / 12	1,18
12	7	F/YxC	yes (10)	2	125	207	0	5 / 21	3 / 21	1,000 / -0,121	0,457 / 0,108	11 / 129	0,001	1,19	7 / 12	1,75
13	7	F/Y/(H)xC	yes (4)	2	123	30	14	9 / 21	0 / 21	0,003 / 2,830	1,000 / -3,243	12 / 126	0,001	1,69	8 / 12	2,17
14	7	YxC	no	2	127	0	0	0 / 21	13 / 21	1,000 / -4,601	0,000 / 5,108	none	no test	0,50	2 / 12	1,86
15	7	YxC	no	4	310	3	0	1 / 21	10 / 21	1,000 / -2,872	0,003 / 2,763	0 / 303	0,01	0,82	3 / 12	1,77
16	6	FxC	no	2	144	55	0	2 / 15	0 / 15	1,000 / -0,023	0,489 / 0,027	14 / 159	0,001	1,59	2 / 10	4,15
17	6	F/YxC	no	5	395	11	0	0 / 15	7 / 15	1,000 / -3,819	0,000 / 3,753	none	no test	0,56	0 / 10	
18	6		no	2	153	74	50	0 / 15	1 / 15	1,000 / -0,226	0,407 / 0235	4 / 183	0,001	0,97	1 / 10	1,10
19	5	F/YxC	yes (4)	2	115	87	0	1 / 10	1 / 10	1,000 / -1,259	0,128 / 1,139	0 / 112	not significant	0,93	3 / 8	2,56
20	5	FxC	yes (4)	2	128	168	0	0 / 10	1 / 10	1,000 / -1,208	0,121 / 1,175	0 / 130	0,05	0,92	3 / 8	1,23
21	5	WxC	yes (6)	2	118	156	20	9 / 10	0 / 10	0,000 / 4,605	1,000 / -3,928	11 / 118	0,001	2,76	8 / 8	2,53
22	5	YxC	yes (4)	2	105	31	20	2 / 10	0 / 10	0,298 / 0,532	1,000 / -0,532	13 / 112	0,001	2,00	2 / 8	2,16
23	5	YxC	yes (4)	2	107	197	20	8 / 10	0 / 10	0,000 / 4,624	1,000 / -4,660	16 / 106		2,61	7 / 8	1,95
24	4	F/YxC	yes (1-2)	2	125	403	50	1 / 6	1 / 6	1,000 / -0,415	0,308 / 0,503	0 / 126		0,80	1 / 6	1,29
25	4	YxC	yes (3-9)	2	132	168	25	3 / 6	0 / 6	0,006 / 2,573	1,000 / -2,822	7 / 136	0,001	1,56	6 / 6	1,36
26	4	YxC	yes (6)	8	392	6	0	0 / 6	3 / 6	1,000 / -2,699	0,004 / 2,738	0 / 396	not significant	0,68	0 / 6	
27	4	F/YxC	no	3	162	10	0	0 / 6	0 / 6	1,000 / -0,437	0,363 / 0,352	0 / 158	not significant	0,90	1 / 6	1,01
28	4	FxC	no	5	373	5	0	0 / 6	6 / 6	1,000 / -3,732	0,000 / 3,502	0 / 339	0,001	0,83	0 / 6	
29	4	FxC	yes (5-7)	2	115	67	0	3 / 6	0 / 6	0,006 / 2,561	1,000 / -2,451	15 / 112	0,001	2,09	5 / 6	2,48
30	4	YxC	yes (4)	3	121	101	75	4 / 6	0 / 6	0,003 / 2,793	1,000 / -3,303	26 / 124		2,45	6 / 6	4,80
31	4	YxC	yes (4-7)	2	119	141	25	0 / 6	1 / 6	1,000 / -2,055	0,020 / 2,087	9 / 128		1,37	1 / 6	1,15
32	4	F/YxC	yes (4)	2	112	168	0	2 / 6	0 / 6	0,035 / 1,825	1,000 / -2,030	4 / 112	0,01	1,35	5 / 6	4,11
33	4	YxC	no	2	150	0	0	0 / 6	5 / 6	1,000 / -4,262	0,000 / 4,647	1 / 162	0,001	1,03	0 / 6	
34	4		no	none	122	369	25	1 / 6	0 / 6	0,070 / 1,488	1,000 / -1,360	12 / 121	0,001	1,68	3 / 6	1,97
35	4		no	4	126	not calculated	25	0 / 6	0 / 6	1,000 / -0,397	0,352 / 0,382	0 / 124	0,05	1,07	3 / 6	1,41
36	3		no	4	139	62	0	0 / 3	2 / 3	1,000 / -1,545	0,057 / 1,588	not tested			0 / 4	
37	3		no	3	319	5	0	0 / 3	2 / 3	1,000 / -2,639	0,006 / 2,528	not tested			1 / 4	1,72
38	3		no	3	337	26	0	0 / 3	0 / 3	1,000 / -0,318	0,377 / 0,313	not tested			1 / 4	1,48
39	3	FxC	no	1	333	6	0	0 / 3	2 / 3	1,000 / -3,417	0,000 / 3,404	not tested			0 / 4	
40	3	FxC	no	2	152	43	0	0 / 3	0 / 3	1,000 / -0,962	0,131 / 1,125	not tested			2 / 4	1,27
41	3	Y/FxC	yes (3-4)	2	118	302	33	0 / 3	1 / 3	1,000 / -2,174	0,021 / 2,057	not tested			0 / 4	
42	3	FxC	no	3	144	8	0	1 / 3	0 / 3	0,018 / 2,126	1,000 / -2,076	not tested			4 / 4	1,47
43	3	F/YxC	yes (1-4)	2	124	182	33	0 / 3	3 / 3	1,000 / -4,233	0,000 / 4,328	not tested			0 / 4	
44	3	YxC	yes (4)	2	114	312	100	2 / 3	0 / 3	0,001 / 3,362	1,000 / -3,177	not tested			3 / 4	1,68
45	3	F/YxC	yes (3)	2	104	221	100	0 / 3	0 / 3	1,000 / -0,120	1,000 / -0,109	not tested			1 / 4	1,12
46	3	F/YxC	yes (3-4)	2	112	211	0	2 / 3	0 / 3	0,019 / 2,105	1,000 / -2,083	not tested			1 / 4	2,86
47	3	FxC	yes (1-3)	8	197	141	67	0 / 3	1 / 3	1,000 / -2,338	0,006 / 2,541	not tested			1 / 4	1,01
48	3	F/YxC	no	5	183	7	0	0 / 3	2 / 3	1,000 / -1,997	0,015 / 2,202	not tested			1 / 4	1,15
49	3	YxC	yes (4)	2	118	100	0	1 / 3	0 / 3	0,036 / 1,812	1,000 / -1,626	not tested			2 / 4	1,70
50	3	YxC	yes (4)	2	102	21	0	3 / 3	0 / 3	0,001 / 3,248	1,000 / -3,159	not tested			3 / 4	3,32
51	3	F/YxC	no	2	156	8	0	0 / 3	1 / 3	1,000 / -1,542	0,066 / 1,515	not tested			0 / 4	
52	3	YxC	no	3	157	0	0	0 / 3	2 / 3	1,000 / -4,729	0,000 / 4,299	not tested			0 / 4	
53	3		no	3	268	6	0	0 / 3	3 / 3	1,000 / -5,529	0,000 / 5,529	not tested			0 / 4	

Additional file 3

54	3		no	4	196	35	33	0 / 3	2 / 3	1,000 / -3,193	0,005 / 2,646	not tested			0 / 4	
55	3		no	4	272	8	0	0 / 3	0 / 3	1,000 / -0,160	0,441 / 0,149	not tested			1 / 4	1,03
56	2	FxC	no	4	161	21	0			0,214 / 0,796	1,000 / -0,814	not tested			not tested	
57	2	½	no	4	188	0	0			1,000 / -4,041	0,000 / 3,904	not tested			not tested	
58	2		no	none	159	0	0			1,000 / -6,951	0,000 / 7,163	not tested			not tested	
59	2	F/YxC	yes (4)	2	137	152	0			0,113 / 1,218	1,000 / -1,050	not tested			not tested	
60	2	YxC	no	2	131	43	100			1,000 / -1,998	0,027 / 1,944	not tested			not tested	
61	2	WxC	yes (3)	2	117	199	50			0,034 / 1,844	1,000 / -1,728	not tested			not tested	
62	2	½	no	1	149	17	0			1,000 / -1,093	0,161 / 0,995	not tested			not tested	
63	2	½	yes (4)	1	101	159	50			0,231 / 0,737	1,000 / -0,734	not tested			not tested	
64	2	WxC	yes (3)	3	114	88	100			0,000 / 3,384	1,000 / -3,651	not tested			not tested	
65	2	YxC	yes (4)	1	107		0			0,001 / 3,322	1,000 / -3,699	not tested			not tested	
66	2	YxC	yes (2)	3	111		0			1,000 / -1,159	0,143 / 1,070	not tested			not tested	
67	2		no	4	144		50			0,072 / 1,470	1,000 / -1,446	not tested			not tested	
68	2		no	none	121		0			1,000 / -2,740	0,005 / 2,628	not tested			not tested	
69	2		no	3	213		0			0,417 / 0,211	1,000 / -0,195	not tested			not tested	
70	2		no	none	121		0			0,453 / 0,117	1,000 / -0,140	not tested			not tested	
71	2		no	2	188		0			1,000 / -1,130	0,130 / 1,131	not tested			not tested	
72	2	½	no	6	179		0			1,000 / -3,908	0,000 / 3,691	not tested			not tested	
										Positive select.	Purifying selec.					

1) Indicates if the family has the YxC-motif in the N-term. of the mature protein. The symbol ½ indicates that some members have and others do not have.

2) The presence of a cysteine close to C-terminal and the distance to the C-terminal

3) Conserved Cysteines are in the mature protein. I some cases there are a few members which are truncated and therefore missing the terminal cysteine, but in the table is counted anyway

4) Length of proteins: The average length of the proteins were calculated for each family. If the average length was below 150 AA, it was coloured light green, if the average length was more than 300 it was coloured grey

5) Gene expression ratio in haustoria-samples versus epiphytic samples and calculated as averages for each family. Colour codes: Orange: >100x, yellow: 50-100x, light yellow: 10-50x

6) Percentages of CSEPs in each family found only in haustoria samples by proteome analysis. Yellow indicate more than 20%

7) Codon-based Test of Positive and Purifying Selection. The two left columns show the numbers of pairs with significant positive selection (z-tests at 5% level) compared to total number of pairs within each family.
The two right columns show the values of P less than 0.05 are considered significant at the 5% level (modified Nei-Gojobori (assumed transition/transversion bias = 1))
The test statistic (dN - dS) and (dS - dN) are shown for positive and purifying selection respectively. dS and dN are the numbers of synonymous and nonsynonymous substitutions per site, respectively.

8) Codon-based calculations of positive and purifying selection using the Selecton-server and based on a Bayesian inference approach (Doron-Faigenboim et al. 2005).
Left column indicate the number of codons under positive or purifying selection.
The middle column shows the significant levels of Model M8a versus model M8.
The right column shows the average Ka/Ks-values calculated on the mature proteins. Pink: Purifying selection Ka/Ks < 0,75, Yellow - orange: Positive selection, stronger colour means stronger positive selection

9) Ka/Ks-value based on method 7 of Liberles (2001) and calculated by service at Bergen Center for Computational Sciencehttp://services.cbu.uib.no/tools/kaks.
The Ka/Ks-values are calculated on each branch point on a calculated phylogenetic tree
