# Supplementary material for: Structure and evolution of barley powdery mildew effector candidates
Source: BMC Genomics. 2012 Dec 11;13:694. doi: 10.1186/1471-2164-13-694 (PMC3582587; doi:10.1186/1471-2164-13-694)
Supplement: Additional file 7 — CSEPs with relationships to ribonucleases. Seventy-one CSEPs showing relationship to ribonucleases were identified by either InterProScan analysis for the identification of functional domains or by structural annotation through analysis of structural templates from IntFOLD predictions. CSEPs are sorted according to family number. [file 1471-2164-13-694-S7.pdf]

Additional File 7.

Seventy two CSEPs that show a relationship to ribonucleases as identified by either InterProScan analysis for identification of functional domains or by structural annotation through analysis of structural templates from IntFOLD predictions.

CSEP ID	Family	InterProScan	Structural annotation
CSEP0003	2	ribonuclease activity	
CSEP0006	2	ribonuclease activity	Ribonuclease Ms
CSEP0009	2	ribonuclease activity	
CSEP0010	2	ribonuclease activity	
CSEP0011	2	ribonuclease activity	Ribonuclease U2
CSEP0019	2	ribonuclease activity	
CSEP0020	2	ribonuclease activity	
CSEP0021	2	ribonuclease activity	Ribonuclease T1
CSEP0022	2	ribonuclease activity	Ribonuclease U2
CSEP0023	2	ribonuclease activity	Ribonuclease T1
CSEP0024	2	ribonuclease activity	Ribonuclease T1
CSEP0025	2	ribonuclease activity	
CSEP0187	2	ribonuclease activity	
CSEP0243	2	ribonuclease activity	
CSEP0245	2	ribonuclease activity	
CSEP0246	2	ribonuclease activity	
CSEP0247	2	ribonuclease activity	
CSEP0281	2	ribonuclease activity	
CSEP0299	2	ribonuclease activity	Ribonuclease T1
CSEP0300	2	ribonuclease activity	Ribonuclease U2
CSEP0314	2	ribonuclease activity	
CSEP0315	2	ribonuclease activity	Ribonuclease U2
CSEP0316	2	ribonuclease activity	Ribonuclease T1
CSEP0317	2	ribonuclease activity	Ribonuclease Ms
CSEP0318	2	ribonuclease activity	
CSEP0375	2	ribonuclease activity	
CSEP0484	2	ribonuclease activity	
CSEP0038	3	ribonuclease activity	
CSEP0076	3	ribonuclease activity	
CSEP0217	3	ribonuclease activity	
CSEP0218	3	ribonuclease activity	
CSEP0237	3	ribonuclease activity	
CSEP0259	3	ribonuclease activity	
CSEP0348	3	ribonuclease activity	
CSEP0459	3	ribonuclease activity	
CSEP0461	3	ribonuclease activity	
CSEP0462	3	ribonuclease activity	
CSEP0469	3	ribonuclease activity	
CSEP0090	12		Ribonuclease U2
CSEP0091	12	ribonuclease activity	Ribonuclease U2

CSEP0092	12	ribonuclease activity	Ribonuclease U2
CSEP0093	12		Ribonuclease Ms
CSEP0197	12		Ribonuclease T1
CSEP0416	15	ribonuclease activity	
CSEP0128	16		Ribonuclease U2
CSEP0129	16		Ribonuclease U2
CSEP0130	16		Ribonuclease U2
CSEP0252	17	ribonuclease activity	Ribonuclease Ms
CSEP0389	17	ribonuclease activity	
CSEP0463	17	ribonuclease activity	
CSEP0101	20		Ribonuclease T1
CSEP0102	20		Ribonuclease T1
CSEP0064	21		Ribonuclease U2
CSEP0066	21		Ribonuclease T1
CSEP0402	26		Ribonuclease Ms
CSEP0480	26	ribonuclease activity	
CSEP0235	28		Ribonuclease Ms
CSEP0027	41		Ribonuclease Ms
CSEP0037	56	ribonuclease activity	Ribonuclease U2
CSEP0078	56	ribonuclease activity	Ribonuclease U2
CSEP0140	61		Ribonuclease U2
CSEP0132	62		Ribonuclease T1
CSEP0347	62	ribonuclease activity	Ribonuclease Ms
CSEP0475	72	ribonuclease activity	
CSEP0476	72	ribonuclease activity	
CSEP0008			Ribonuclease U2
CSEP0017			Ribonuclease T1
CSEP0018		ribonuclease activity	Ribonuclease Ms
CSEP0044		ribonuclease activity	
CSEP0219		ribonuclease activity	
CSEP0342		ribonuclease activity	Ribonuclease Ms
CSEP0344			Ribonuclease U2
