# Supplementary material for: Structure and evolution of barley powdery mildew effector candidates
Source: BMC Genomics. 2012 Dec 11;13:694. doi: 10.1186/1471-2164-13-694 (PMC3582587; doi:10.1186/1471-2164-13-694)
Supplement: Additional file 8 — The B. graminis EST sources that provide evidence for expression of the CSEPs. A total of almost 52000 EST sequences were searched, but some of the libraries were mixed with barley transcripts and the total number of fungal transcript therein is unknown. The number of CSEP in the table indicates how many of the CSEPs we found represented in the different EST projects. However, in many cases there were several hits, so the number of CSEP ESTs is much larger. The EST library with most CSEP hits is the epidermal EST made from epidermal cells containing many haustoria but no other fungal material [6], and here we found 151 different CSEPs, but the total number of CSEP ESTs was 1299, which was 20% of the total number of fungal transcripts. [file 1471-2164-13-694-S8.pdf]

Additional File 8. The *B. graminis* EST sources used for expression evidence of the *CSEPs*.

A total of almost 52000 EST sequences were searched, but some of the libraries were mixed with barley transcripts and the total number of fungal transcript is unknown. The number of *CSEPs* in the table indicates how many of the *CSEPs* we found represented in the different EST projects. However, in many cases there were several hits, thus the absolute number of *CSEP* ESTs is much larger. The EST library with most *CSEP* hits is the epidermal EST library made from epidermal cells containing many haustoria but no other fungal material (Godfrey et al 2010). Here we found 151 different CSEPs, but the total number of *CSEP* ESTs was 1299, which was 20% of the total number of fungal transcripts.

**EST-project/ or database**	**cDNA source**	**No. of seq.**	**No. of *CSEPs***	***Bgh* isolate**	**Reference**
COGEME (University of Exeter)	Primarily conidia and germinating conidia	6944, (3241 unisequences)	38	C15, mixed	Thomas et al. 2001
Epidermal-EST-library	Epidermal cells without epiphytic material, 8 dpi	9928, (~6512 fungal ESTs made into ~3200 fungal unigenes)	151	A6	Godfrey et al. 2010
Conidia	Conidia	5174	25	DH14	Spanu et al. 2010
20 hpi	Germinating conidia 20 hpi	1280	22	DH14	Spanu et al. 2010
3 dpi	Epiphytic hyphae 3 dpi	4987	20	DH14	Spanu et al. 2010
6 dpi	Epiphytic hyphae 6 dpi	4873	31	DH14	Spanu et al. 2010
Epidermis-mapped	Epidermal cells without epiphytic material, 7 dpi	448	50	DH14	Spanu et al. 2010
Epidermis non-mapped	Epidermal cells without epiphytic material, 7 dpi	1054	7	DH14	Spanu et al. 2010
Cleistothecia	Cleistochecia 3 month after inoculation	4438	42	DH14 x CC52	Spanu et al. 2010
454-sequencing	Epiphytic hyphae 5 dpi	12827	110	DH14	Spanu et al. 2010
