# Supplementary material for: Structure and evolution of barley powdery mildew effector candidates
Source: BMC Genomics. 2012 Dec 11;13:694. doi: 10.1186/1471-2164-13-694 (PMC3582587; doi:10.1186/1471-2164-13-694)
Supplement: Additional file 10 — Level of diversity at the nucleotide level in pairwise comparisons between members of three CSEP families. Diversity was calculated as percentage of different nucleotides for the two exons, the intron and the 500 bp up- and downstream to the coding region. In case there is no homology in parts of the up- and downstream regions only the homologous region was used for the calculation. [file 1471-2164-13-694-S10.pdf]

Additional File 10. ***CSEP* nucleotide diversity within families**

The table shows the level of diversity at the nucleotide level in pair-wise comparisons between members of three *CSEP* families. Diversity is calculated as percentage of different nucleotides for the two exons, the intron and the 500 bp up- and downstream of the coding region. In case there is no homology in parts of the up- and downstream regions only the homologous region is used for the calculation.

**Family 7**	% differences at nucleotidelevel				
Pair-wise comparison	500 bp upstream	1. exon	intron	2. exon	500 bp downstream
CSEP0301 - CSEP0302	1,7	0,0	1,7	1,8	0,6
CSEP0301 - CSEP0304	10,5	13,7	6,8	9,5	7
CSEP0301 - CSEP0305	12,3	13,7	11,9	10,7	8
CSEP0301 - CSEP0306	12,0	14,3	11,9	11,2	8,2
CSEP0302 - CSEP0304	11,2	13,7	5,1	8,9	6,8
CSEP0302 - CSEP0305	12,9	13,7	10,2	11,2	8
CSEP0302 - CSEP0306	12,7	14,3	10,2	11,8	8
CSEP0304 - CSEP0305	6,7	1,7	5,1	7,1	6,4
CSEP0304 - CSEP0306	7,1	1,7	5,1	7,7	6,4
CSEP0305 - CSEP0306	1,3	1,1	0,0	0,6	0,4
Average of all pairs	8,8	8,8	6,8	8,0	6,0
Length of fragments	< / = 500	175	59	169	< / = 500
**Family 8**	% differences at nucleotide level				
Pair-wise comparison	500 bp upstream	1. exon	intron	2. exon	500 bp downstream
CSEP0147 - CSEP0148	9,5	21,3	7,8	25,7	11,2
CSEP0147 - CSEP0149	10,3	23,4	23,5	25,7	12,7
CSEP0147 - CSEP0150	10,2	24,7	19,6	29,2	12,7
CSEP0147 - CSEP0151	12,0	25,5	17,6	31,0	11,3
CSEP0148 - CSEP0149	9,0	17,4	15,7	24,8	12,0
CSEP0148 - CSEP0150	9,6	26,4	11,8	28,3	9,3
CSEP0148 - CSEP0151	6,9	24,7	9,8	30,1	11,9
CSEP0149 - CSEP0150	1,0	20,9	17,6	15,0	13,3
CSEP0149 - CSEP0151	10,7	23,0	15,7	27,4	15,8
CSEP0150 - CSEP0151	10,9	26,4	15,7	32,7	
Average of all pairs	9,0	23,4	15,5	27,0	12,3

Length of fragments	< / = 500	235	51	113	< / = 500
**Family 30**	% differences at nucleotidelevel				
Pair-wise comparison	500 bp upstream	1. exon	intron	2. exon	500 bp downstream
CSEP0069 - CSEP0070	5,6	7,1	5,2	8,0	4,6
CSEP0069 - CSEP0071	0,8	12,6	5,2	10,6	6,0
CSEP0069 - CSEP0072	6,4	17,8	6,9	28,3	8,4
CSEP0070 - CSEP0071	6,0	14,6	1,7	13,3	4,6
CSEP0070 - CSEP0072	6,8	19,8	3,4	33,6	10,8
CSEP0071 - CSEP0072	6,8	18,2	1,7	31,9	9,3
Average of all pairs	5,4	15,0	4,0	20,9	7,3
Length of fragments	< / = 500	250	58	113	< / = 500
