# Supplementary material for: Structure and evolution of barley powdery mildew effector candidates
Source: BMC Genomics. 2012 Dec 11;13:694. doi: 10.1186/1471-2164-13-694 (PMC3582587; doi:10.1186/1471-2164-13-694)
Supplement: Additional file 12 — Distribution of codons under selection in selected CSEP families. Protein sequences and distribution of the amino acids under positive and purifying selection in families 1–35. The residues are coloured according to their calculated Ka/Ks-values, estimated using the Selecton server ( [49,50] http://selecton.tau.ac.il/). Codon sites under positive diversifying (red) or purifying (purple and yellow) selection are highlighted. The conserved cysteines are shown in yellow. [file 1471-2164-13-694-S12.pdf]

Additional File 12

Family 1: Positively selected sites found. Sequence is CSEP0228

1 2 3 4 5 6 7
Positive selection Purifying selection

1	11	21	31	41
MSCAIAILLN	TVGRLVVTGL	DNGLSHYGVY	NVPLTYQFLV	PEEKLGIHVS

51	61	71	81	91
TSQITQPDTH	VVAYCSIINN	LSQLVDIITK	DIPDITRSPK	LSDSEYVDLE

101	111	121	131	141
TKCRSYVERK	SLATLELSMS	ELKKDGHCTD	DNLASLNFQG	ILGVSGNYSC

151	161	171	181	191
FAAPVESPSF	NITADEPIRL	ESFLSEGELL	RDRGLVIRPN	AMAWFQGHLH

201	211	221	231	241
IFEQRVSDDD	DHTWWYPLTS	IGQEDTNAPI	IVEFIYQAIP	QISWFMNVII

251	261	271	281	291
GFNKLMVTTS	LPGLMLNASQ	EESSKTYQNR	NPENLDHPKY	IYDEEFLERR

301	311	321	331	341
KDYIQFKFCK	DTSKLLNNLG	LPTSEYDESF	GCHVPKYMSD	EESIGQLKMI

351	361	371	381	391
KLPPQSDTSL	PAETFTRQPG	EGWLCDERDL	WADGPPKKAP	SISRKLQ

Additional File 13. Continued: Family 2: Positively selected sites found. Sequence is CSEP0006

1	11	21	31	41
MWINLCVTLS	ISWLILQVSC	DNIPDSDMYL	PDGTNGFVCD	LDFYSIDHVR
51	61	71	81	91
EAAKKGVEAF	FYKRKVDKFP	KTFEDTQLYN	VKSDILLSWP	IISSGLFYYR
101	111	121	131	141
TPGNSRLIIN	TRGQIMGIVV	ISFQKPSHKI	SYNKCIPVRR	SLEENNKGQS
151	161	171	181	191
LLNECWGLAT	PTFGYNCGSK	FFPKSILDKM	IGPDAIKNYQ	QRLSGRNKLS
201	211	221	231	241
CLIKYTGDEF	SGVDLYWYPL	HQKLSDKRLA	GPSSRFRVVF	DISNSEFKGI
251	261	271	281	291
IDVENRNKKC	VTVWDVSSIS	SNNIYISSST	LNLDKVGDGH	WPQTCFGHKL
301	311	321	331	341
KSKIMWLYLE	FALKYWMSTL	NESNPNLPLT	SQKFIYSWFI	RPGETNKDSS
351	361	371	381	391
NHVFAIAYNT	KHNNYKLYLM	KTRNVKVDVH	NPCLEFSTDD	IDRLRKHIGD
401	411			
KHNPEESLSI	DR			

Additional File 12. Continued: Family 3: Positively selected sites found. Sequence is CSEP0217

1	11	21	31	41
MKTSFALYLA	LTTLQVGIKG	SVIGENDYIC	GTDVITAANI	FKSIDKACHT
51	61	71	81	91
LYHSRKVIRY	PADYDASATF	FISDARLFSW	PLMKDERIFN	TGRAGNIRLI
101	111	121	131	141
IDSSCRFIGI	IQKTINQPDQ	RCFRPMKLPR	DMVDSSNPLP	GRLPVLRGYS
151	161	171	181	191
CGGIVFDSVQ	VEKVRQETLK	EFQRWNSLNH	QISLTTLRVV	QELYGSMVFL
201	211	221	231	241
LPKKTPVSPT	QLLRLADYSC	FIVIDSNHKT	LGMVSRSIDN	WDKCDELWEM
251	261	271	281	291
EPERHHIHHP	QKNSVGETVF	EGLTDYECGP	FKFLRETINS	HMQIACTIFQ
301	311	321	331	341
KIQMSSAHFQ	RGRLCSAQID	TDTGSFEVWK	FPLQLPERDL	ITEHILSSNN
351	361	371	381	391
CVIMLDEQCY	FHGVYMYSKK	TKEKCNQLFP	QYVQETLDYE	DEISDLSWVD
401				
EMIVDWSSPL	K			

Additional File 12. Continued: Family 4: Positively selected sites found. Sequence is CSEP0154

```
1          11         21         31         41
MRLSRIAMIF QSASFCTISL AARFMVHPHE DSKVFFCDME FEQETYARID

51         61         71         81         91
REKIIDTGTI QTLNEELNRL VADTTDQRFV QFYDTDNGGY EYFNFPTRFR

101        111        121        131        141
KRYDGNDLAI TEYIMVIDQQ GRPCSMMMSK TLVLKGIRNR RIVQRSYSLC

151        161
AVGTKYSPQW RQI
```

Family 5: Positively selected sites found. Sequence is CSEP0118

```
1          11         21         31         41
MKIFSPASTA ALACLLLVVP VALAVGYFQC SHDERFSLAY IQHKDSQCEA

51         61         71         81         91
RHAKDDEPRG PNGEVYRTTH IMTVPRVGFF VYYIIQVIDD SPTYRLYEDL

101        111        121
SNEWEPCVYQ ETNKHTDSDS DESDI
```

Additional File 12. Continued: Family 6: Positively selected sites found. Sequence is CSEP0015

```
1          11         21         31         41
MRPLALLTAI PVFYSVYVAG DNAYHCSDSE GNTNTTYDQA LLEGSVKDTC

51         61         71         81         91
LPNSELSAAQ LAANVEAKKY PQVWVDSQDY GFNGTVLLWK YQGNSSEGLD

101        111        121        131        141
ESDALIFTDN ACNIMGLVQK SKGFYTICKG AHEHFDEKVI TNTMHKNVQR

151        161        171        181        191
RWIFGNDDDD NDEDDEVNKL HGFAGSSSDN AITLSPNIER EYDNYNRGRK

201        211        221        231        241
GWSYSRKVPK YLFPDPPPTL QQADDDTSSY ASTDYGTASQ SFSSTNGDDS

251        261        271        281        291
SQYNEGSEYD YGSQYQGGSS SSYQKSRSKK KGFVSGLFGS SRKQRYLPAT

301        311        321        331        341
ADFPSTRNPP KPDLSRGRPS GKIIITPGCP TRPPPNDGYT MRPYRPTFEY

351        361        371        381        391
GPDGTILLIT DKVTTMDKVT TMDKVITMDK IIPNQCYNIR IIFLKWVDAH

401        411        421
DTRQDSFKKI SKDLVTLCFR FSRAVFL
```

Additional File 12. Continued: Family 7: No positively selected sites found! Sequence is CSEP0058

```
1          11         21         31         41
MRFFLFILAL MVAGFMAVPT GPYIVRRTNK KTAHTKFLET VDGFRWQDKV

51         61         71         81         91
DTWTEKTRIT IENVLEKIST FYTDKFGKKA GSIPKPSGSK SLSPAVHDAS

101        111        121        131        141
HSNHTKRFLQ QDPFGYRLDG EKTFSYYNKK SKVHLDHLFR EYQNSSA
```

Family 8: Positively selected sites found. Sequence is CSEP0058

```
1          11         21         31         41
MKFISTATTA ALAGILLLAP ATYGNRYYKC ELSDPVSEAD VIKMVSDRTL

51         61         71         81         91
EKNADIHPKV PTGESHKSYM FMKRTYANRP RITAYLVQVY GDPKEYQLSL

101        111
HAGGRWHLCS LEDES
```

Additional File 12. Continued: Family 9: No positively selected sites found. Sequence is CSEP0290

1 11 21 31 41
MQIFILFLAL FMTGLMAVPI SIHGIGSLSH RNIGDVNPNI IENPNSVNNV

51 61 71 81 91
KIFGNGVDRT DLMQQIASTK ESNRFLGENN ADVGTSLSLT NDNFPVTRSG

101 111 121 131 141
GYLSALTYNP TRVMGGVAPP RKHAKRALYQ SPSGFQLDGK HRFPYYNQRS

151 161 171 181
IRRVNGLVND HKILSTNRRL RRPSVQRKPL LLNKEKSSSP

Family 10: Positively selected sites found. Sequence is CSEP0029

1 11 21 31 41
MRFFNMAFIL QSAALFLPAL ASYKISHTNE EEKKFMCYNR AIYARDYWEE

51 61 71 81 91
KLYADRLVQT KQKTEISMKD ITNISWPTNK SDLVLYDDNN ALHYYFHMLT

101 111 121 131 141
SIADSVNIRG QTVLIRYSLL IDNYGRVCSI VMHEQGASES ETRLLHDVIP

151
ELTHPFCVLF G

Additional File 12. Continued: Family 11: Positively selected sites found. Sequence is CSEP0047

```
1          11         21         31         41
MKFPVSLVTI AISTVALAYC VPPSSQPTLP EGQEVISNSL KVPGENALEY

51         61         71         81         91
CSSEFLSYPI RLFNVAIKPT IPTINEQVEV FVDGFTGIPL SPEVQLRVFD

101        111        121        131        141
WASGRDLSEP INFFKYMNSI NAQVPDHVGQ FRLFFRFKPE PTILYSGQIG

151        161
IELNYRKDFK LLCVKTPIRI F
```

Family 12: Positively selected sites found. Sequence is CSEP0092

```
1          11         21         31         41
MKFLHSTEIT TILSLFASLC ATQGVNDGYP MYNCRGVIFY SDSVTNSAAR

51         61         71         81         91
AHRLKMGEIN GYPQKLNSFN ADGPPPHRIF PMVRDEDVYS GGIESFYFVI

101        111        121
VDRSGQQQGV AYSVADGYMP CQTIVNPEY
```

Additional File 12. Continued: Family 13: Positively selected sites found. Sequence is CSEP0125

1 11 21 31 41
MKIFSLISLA AILSHLTPAI ANQNYKCHDQ IIGPIPLMES VNNAFKYYNE

51 61 71 81 91
EAKPGPADKL FIPSAFDLKI KYKDGTFDVK IEVGANSQKQ IIYVKAYCQG

101 111 121
KTFDCTPTNE PPTYNKLQPK AENTKM

Family 14: No positively selected sites found in the protein. Sequence is CSEP0364

1 11 21 31 41
MSIFISIFFL ISCLTLVYPI NGMKIERRFI GSVHKGYQCN KKKHNMYDIE

51 61 71 81 91
PTLKSACKAY IKRKQKKWFF IYFINRTVFK RFSESKRFGF PPDTQITPVL

101 111 121 131
PKGLGKISLY VMLTLISSGI YWQTPRIFIC VEA

Additional File 12. Continued: Family 15: Positively selected sites found. Sequence is CSEP0418

1 11 21 31 41
MKNLYHTFRC QSILLKAIFF MLLTSANASL TLQYVKRSME PGYHCHNKLY

51 61 71 81 91
SFGEIEKVRK LACKAFLSTR DDARRPIVRQ EYINVENLIY EWSLPANPTI

101 111 121 131 141
NPTIHPTDQT RKYTGKIIFN NRCELIDVAC YERKTKQEYL CQKVSKVSSS

151 161 171 181 191
TTSGDDKVLE EPFVQCGSLA LKTEEIREYA RSKSFDSLLE FHEVRETLGR

201 211 221 231 241
IDGPWKRALF SKELIIRHRS NDILYEIVVD KHDEVRGMVV THHISRKLTS

251 261 271 281 291
AEAHDGEIIK GPRKAKLKKH TIRLVCFFHK TFPLLRPALI SDVSNPLKRK

301
ALV

Additional File 12. Continued: Family 16: Positively selected sites found. Sequence is CSEP0343

1	11	21	31	41
MKLFTISGAA	KLFFILAPVI	AMPDNYHSGS	TPVPDQDNDF	SFYCPSDQIY
**51**	**61**	**71**	**81**	**91**
NKNYLISVVQ	EARQIMRDSY	TQYVYPSTFN	HFNYDIIGEL	WHYPLTGNDK
**101**	**111**	**121**	**131**	**141**
PMKFSCFARF	LKLINGFNVL	VSYGAQDFVV	FNTNNDIAGV	AHRKSVNGRS
**151**				
IFEPCSLLG				

Family 18: Positively selected sites found. Sequence is CSEP0229

1	11	21	31	41
MKLLSMTAVA	LLIEFSVSIH	LSEDDGAFIN	EPKLPVLDSN	FGMDCYSDKT
**51**	**61**	**71**	**81**	**91**
YGQITVKSAA	AEAYTNTCNS	PGQMERCTFD	RFPMENLFLH	PIGGGYVSHL
**101**	**111**	**121**	**131**	**141**
GVPAQHYIII	NSAGQFLAGM	LKYETHGNLV	SALCRFTPYT	AFLSYAESRR
**151**	**161**	**171**	**181**	
LNTQGDRNYY	GSQYGQAGQQ	PPIGSNRRSH	SGY	

Additional File 12. Continued: Family 17: No positively selected sites found in the protein. Sequence is CSEP0225

1	11	21	31	41
MHNILLSSFL	VLLHTYLHIT	GSSASPTSNG	KSKLGLSTDD	FVCNGEYVSS

51	61	71	81	91
SIVRQSIDAA	WKALFTTQPN	DVYVNHVEDT	RIFGGTSKTL	FSGPIPIHGD

101	111	121	131	141
YGLPDEGFKT	IARVVFDFQR	HLVGIITIHK	NGVRHCREII	GASNPASFDA

151	161	171	181	191
NLHDLLGYKC	ANEVFDYRYV	ASSYTEAYNE	LSNGSIRPGG	YPKIIDRPGS

201	211	221	231	241
PHGAQFSWPL	LPYSKLYNHG	DATYNEFISF	TDSIETLSVY	YTDNYREHIC

251	261	271	281	291
PAIWTNKTPA	LSNPDFLMKH	YRAPESSDSL	IHCHGKSFEP	HFVADNIKIA

301	311	321	331	341
KRAHEFRSKH	KLDLLFPEPI	GDESASPGQS	NWLWPLRIQE	DFHQVRQRSI

351	361	371	381	391
EYFLVLDGSF	EFVGVFSFEN	KNYSPCLSEG	SKNLSHDMPQ	DYLPQ

Additional File 12. Continued: Family 19: Positively selected sites found. Sequence is CSEP0139

```
1          11         21         31         41
MKVLLAASTA GLAAFLLLVP TAYGLHYFKC NSRRIFAYSV IVGYTDSATY

51         61         71         81         91
EQIQAGDPHF AGGHTYGAYR FTDYLPDGSP RNYLVQMVNV NPYYRLFESS

101        111
GGRWHICTME GS
```

Family 20: Positively selected sites found. Sequence is CSEP0063

```
1          11         21         31         41
MKIQNMKWFI ALMFTPKFAY ADYVFNCYGL DVTRELIRRA YLLSMNGYVP

51         61         71         81         91
DYPVITSISY LYNNEPIGIF PILGDDETSI AAELYRPFLI WSQSFLDWGV

101        111        121
YFLGDNGYEI CYKNPAYNGY TFRQIAFGNL
```

Additional File 12. Continued: Family 21: Positively selected sites found. Sequence is CSEP0064

1 11 21 31 41
MRPFQLLSAL AIFINLEAVE AAAYWDCDGT EIPERNVRAA VVLAFNYRKE

51 61 71 81 91
SFHGYPATFI IGSTFSGVGE VRQFPVEDSD ANWQGGAVKY YILTNKRGSY

101 111
LEVFSSVGSG NKCTFVEG

Family 22: Positively selected sites found. Sequence is CSEP0180

1 11 21 31 41
MRLLPLASFI ALMSHLISVS AEYKYVCPSK IKFTTARIDM LRNDISSGSI

51 61 71 81 91
TEGVLIITSS GLRVLTYIFD RKIIDGVAYD FMVTVNYRDK LTRVYEKESE

101 111
HTRICELIFV LD

Additional File 12. Continued: Family 23: Positively selected sites found. Sequence is CSEP0248

```
1          11         21         31         41
MKNLSFVSLA LFLSHLMPVL AVKNYTCGGV VVPAKTIKSE IEKKFDTIPL

51         61         71         81         91
LDGQTYVAAA ECGKTVFEVN PPRNGGSPLS VEIAFNYRQE VMSITATRDN

101
ETVDCK
```

Family 24: Positively selected sites found. Sequence is CSEP0111

```
1          11         21         31         41
MRFPHLVHLS VIFGFFTFES AAYYTCPSGI LIQNDTVRSR AQEIMNLGVQ

51         61         71         81         91
LDVHRTPGQV EMSNITFYGS KAGGDLWFTS DFNPPITTEN TFKISISYPG

101        111        121
EEIVLTEFQK FGSGSQEGTC DVSVEM
```

Additional File 12. Continued: Family 25: Positively selected sites found. Sequence is CSEP0012

1	11	21	31	41
MRTATLLAFV	GTWLGSLAGI	VRFEPEYLCG	NDVHIPVSDV	NNSLNLFQEQ
51	61	71	81	91
AINCQQGFNI	GSPGYLYDGK	PKNRASVSTA	MHAFGFDFID	LNIDHWYEYI
101	111	121	131	
IVYDIDFKLQ	KFEAVRLRGS	KERKVLKCEY	DPRYQD	

Family 27: Positively selected sites found. Sequence is CSEP0034

1	11	21	31	41
MRFSSLAIVC	QIASIFVTSK	AWAILNHIDE	QTKRFNCDGV	YVRYNEFASR
51	61	71	81	91
QRTRLVKSTD	DRNSRAWSLV	VQQLLNNKPM	GEEDPSVMFR	DDASAHLTYY
101	111	121	131	141
KLDDLTKLDN	YGSQSLRTDY	VVAIDGSNRA	CAVILRRQDQ	AWDHSWMGEP
151				
TFQFCTIY				

Additional File 12. Continued: Family 26: Positively selected sites found. Sequence is CSEP0403

1 11 21 31 41
MQFTVRQSTT KFRRLHIILF LTISVIGLTL QADISGYICD DLVVPYDKVV

51 61 71 81 91
EVAKKASEFV KSVDGRYRFP ALLEDTQLFG VEGQNMFVVP FRRFDSMRSG

101 111 121 131 141
GNKGRDRIVL DASGNFKGVV YDTNDGNVHP MPTYRKCRPF LDDKHLELEK

151 161 171 181 191
YIDDGYQISG FACGSNFLDR DVETNLYNEC IRYISLPQKI KDKYKFPRCR

201 211 221 231 241
YAFQKINELW FQYNTNKNYD NKQTKVINSF MATFNNRCEF LNIKRISRVI

251 261 271 281 291
THEEPCSYTW TAIPSAEATM TDSLFDVQSK NYKISDEFYA CKSYIFRITS

301 311 321 331 341
IQNHMFHIFS LLLSNEPSSI EHMMHRQGEL LLWPMTLPER HEPRINHPLT

351 361 371 381 391
FSIGFDRSNQ FSGIYFVTGS EWTWKKAKPC PNFNLLESRL KARGSG

Additional File 12. Continued: Family 28: Positively selected sites found. Sequence is CSEP0234

```
1          11         21         31         41
MIAWTVLSIL ALLPLCFAKP ALRKSHTELF RCNLKYISMV QAQEAVKLGC

51         61         71         81         91
NQMEVAPPAS LFPASFSGSE LFGLVGVGLF TWPVFPSKNV LREDYGLNRV

101        111        121        131        141
VFDSSCRLVG LIHIKPTGPE CCLHLMEIED LKMTHELKVY DTRWDSLQLY

151        161        171        181        191
GYRFDDELYL KNSVEEFVKF NFQSFRRNPS IKNALSMYPN LEQFTKFEIR

201        211        221        231        241
TRPIELGPNG IQQAAVRFNR RLIINSNKEI IGICRKSDNV WKPFAELRIL

251        261        271        281        291
NDVYKLKVIS ARGPERGYEG LHGMNHNGIQ VSTAMLRSHL LMACKVLMER

301        311        321        331
NMGTNTNDEN SMLAKMESTS DRLIFTWPLR MPENFVHGM
```

Additional File 12. Continued: Family 29: Positively selected sites found. Sequence is CSEP0059

```
1          11         21         31         41
MKFLSAASMA TLSGLLLSAT AHAADPYFEC SMNTAVSFSG IIFYEQSHEY

51         61         71         81         91
LDAEPGDPEG PNGEIYPARR FTRVRRDGSD VLILIQSLDE YPLRRAYEKT

101        111
EQGWRLCPFH KP
```

Family 30: Positively selected sites found. Sequence is CSEP0070

```
1          11         21         31         41
MRTSTLLRCL FTWLVTAITT VQCQNDYHCS NNIVVKGDVI QRALNDISTD

51         61         71         81         91
STSTLYAAGT LYVNDVFVNR WWKEMIVPES YYVGGTDTSY RLIYDDNKFV

101        111        121
RSIQLVYHFR LGIRISQCTE VIEE
```

Additional File 12. Continued: Family 31: Positively selected sites found. Sequence is CSEP0105

1 11 21 31 41
MHSNRLLSCL GIWLISIIGL VQCITEYYCG NNVVISQELI QSVSEIAISE

51 61 71 81 91
LRAGVTWHTS LSGIIYRGNL CGLPNPTYRW FIQQSEDENS EVVMKYFLLV

101 111 121
SASCEVQGVI STGWRKKLGE DDTWCTEA

Family 32: Positively selected sites found. Sequence is CSEP0106

1 11 21 31 41
MKFSNTASMA ALAGLSLLIP AVHGTLMFDC GNDARFLTST VMEYLSIATA

51 61 71 81 91
ASIEDDDPPY AQGQVNGAYR FTKNINNVNR TFLVQSTNIH PFYRFFEITS

101 111
GHYECPLKEH YA

Additional File 12. Continued: Family 33: Positively selected sites found. Sequence is CSEP0195

```
1          11         21         31         41
MRSYINIICL AIIAAIIFAA PNNTLSLHRP VLSAMDKRNI GKDHTGYQCG

51         61         71         81         91
STKHKMNKIL DAIQMACPDP AQSDSFWFSY NDSGFPKTFS EAQRLGLPKD

101        111        121        131        141
TMITPVRGGF FDQLQFHYAA FEPVTCKLRG VIRQKDNKKD GVFQLCQYTT

151        161
WPLSWTDPFH PG
```

Family 34: Positively selected sites found. Sequence is CSEP0371

```
1          11         21         31         41
MKTFQFASIV AGLSFLKTTI AAGDDYITLG MTSIKVQHIH SVARDMWTMD

51         61         71         81         91
IDYYKSEYQG RYPAFWGNVR GKPARKYPLL YEGKVWTHGS FMYYVVATED

101        111
LSYIRLYYEG TESCDREVPS P
```

Additional File 12. Continued: Family 35: Positively selected sites found. Sequence is CSEP0380

1	11	21	31	41
MKFLNTVSAS	ALSALILGSV	VQGYVLKCST	GKEFDNQTMD	ELASKAEYQY
51	61	71	81	91
GHESYPLGPD	GEKCKAYEFN	SQLPNSQSSL	ISAYSATDHS	HLARATIPYL
101	111	121		
VQVCDSMESY	RLFEWYDYRW	VKCD		
