# Supplementary material for: Structure and evolution of barley powdery mildew effector candidates
Source: BMC Genomics. 2012 Dec 11;13:694. doi: 10.1186/1471-2164-13-694 (PMC3582587; doi:10.1186/1471-2164-13-694)
Supplement: Additional file 14 — CSEP amino acid alignments of families 1–35. The proteins are aligned using CLC main workbench, as described in Methods. [file 1471-2164-13-694-S14.pdf]

CSEP family 1.

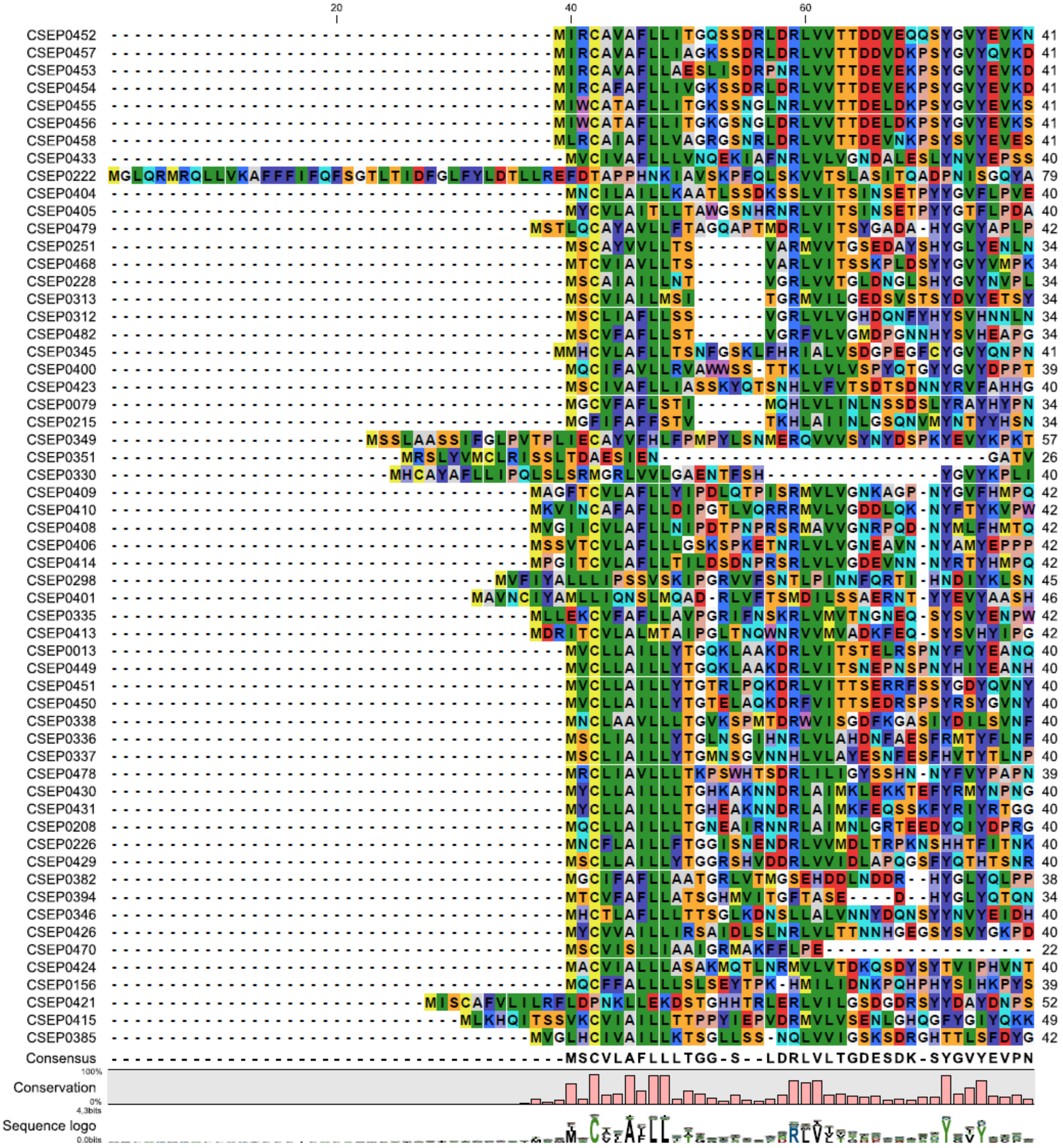

CSEP family 1 (continued).

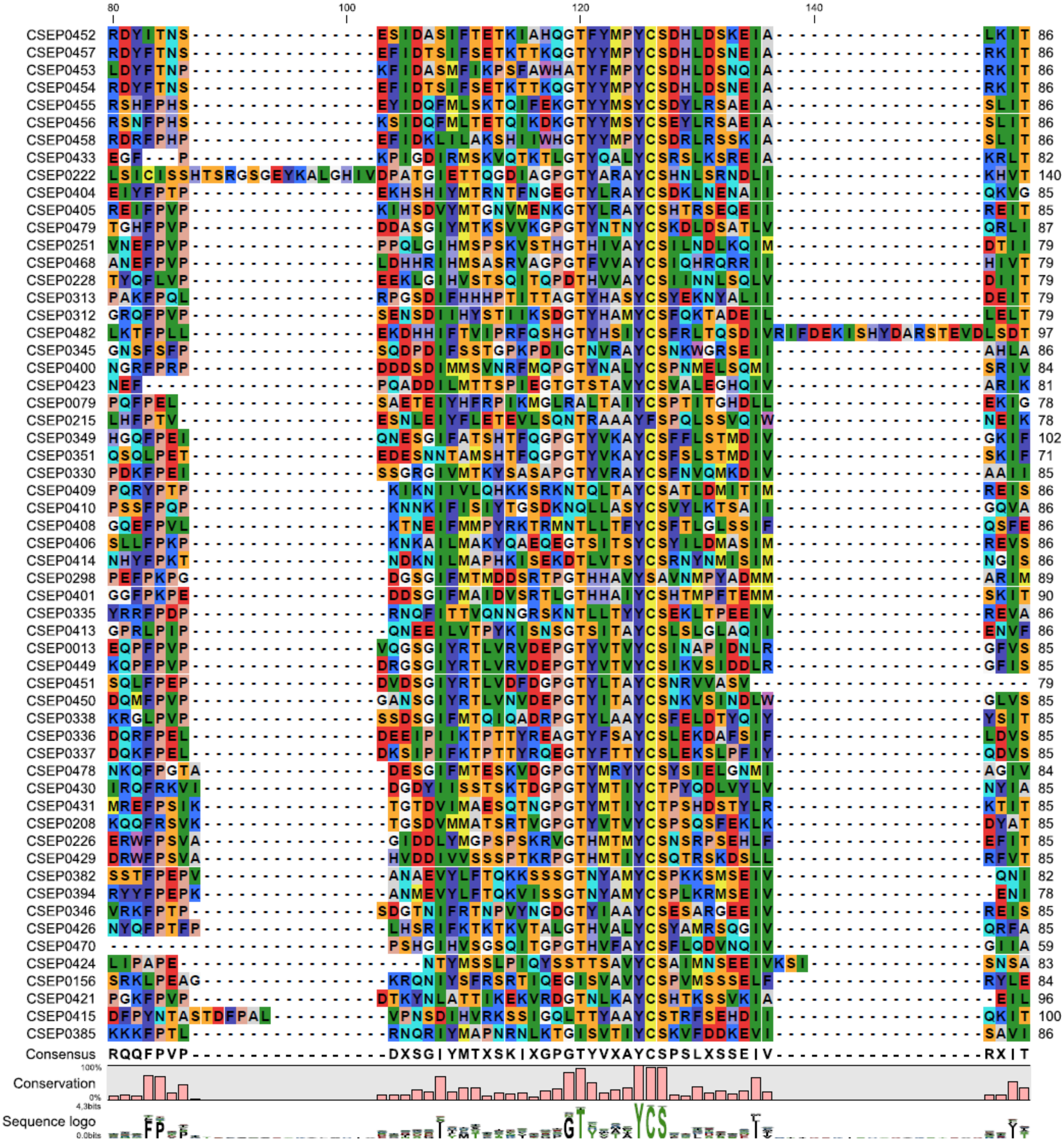

CSEP family 1 (continued).

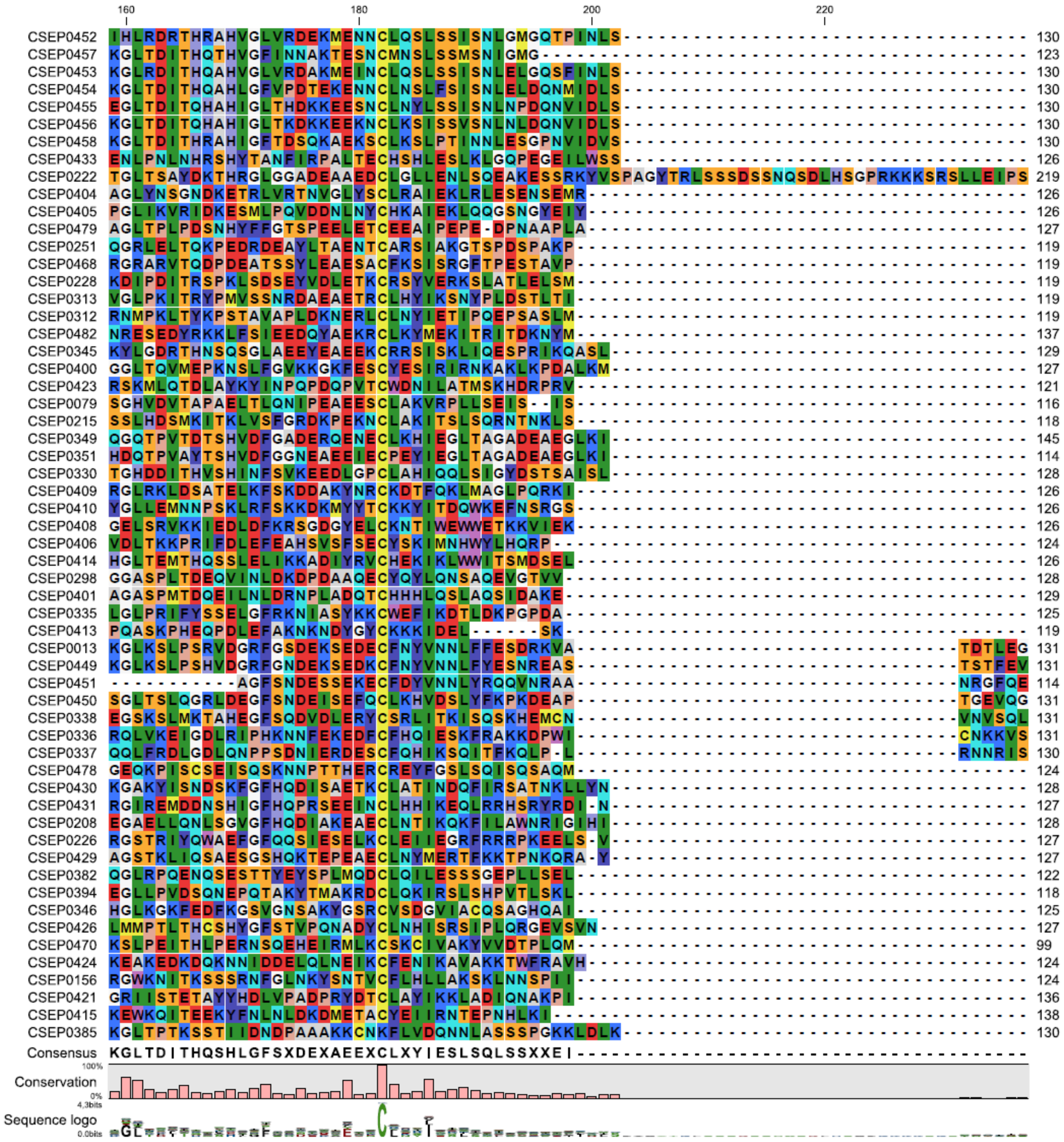

CSEP family 1 (continued).

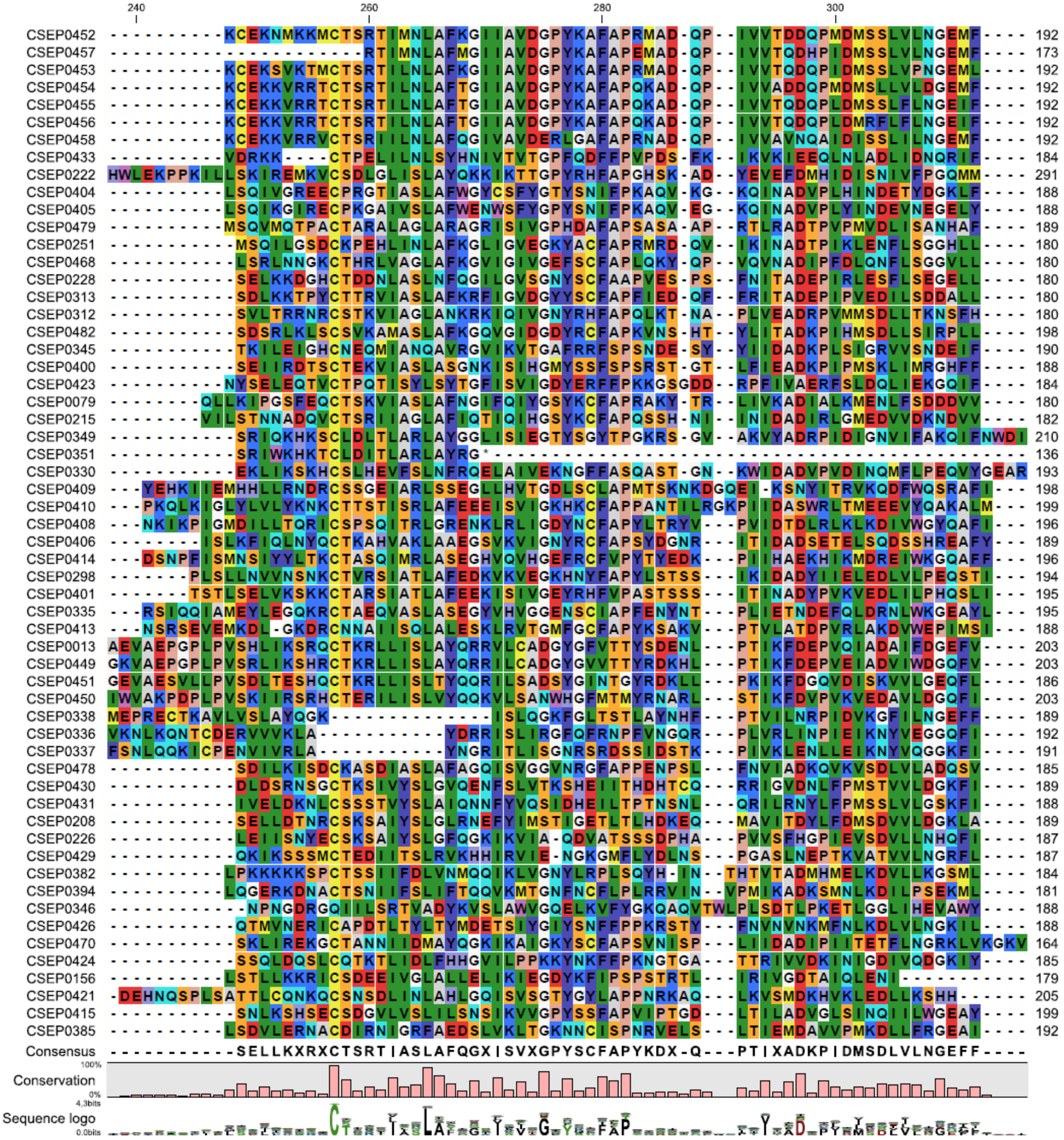

Additional file 14

CSEP family 1 (continued).

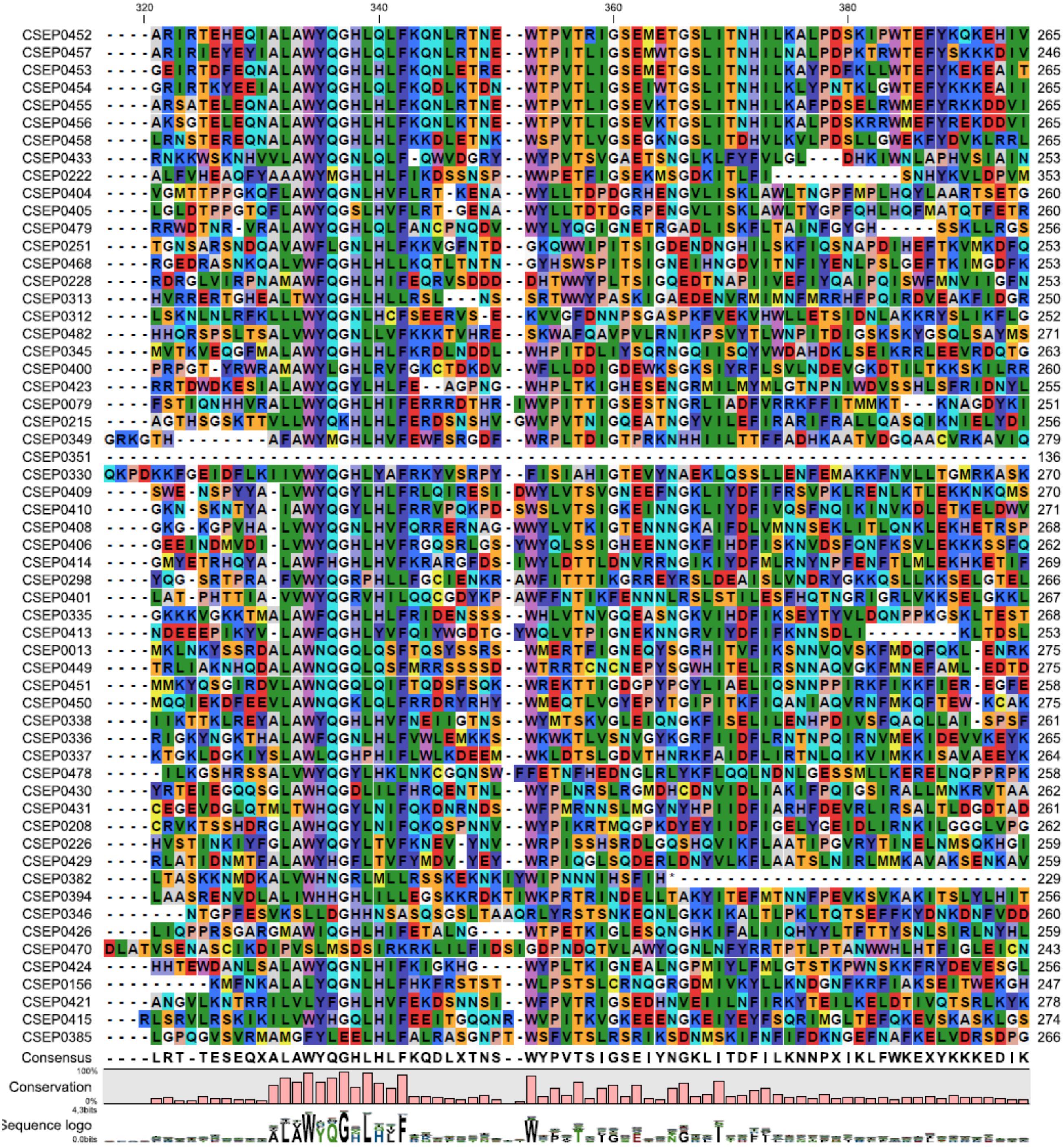

CSEP family 1 (continued).

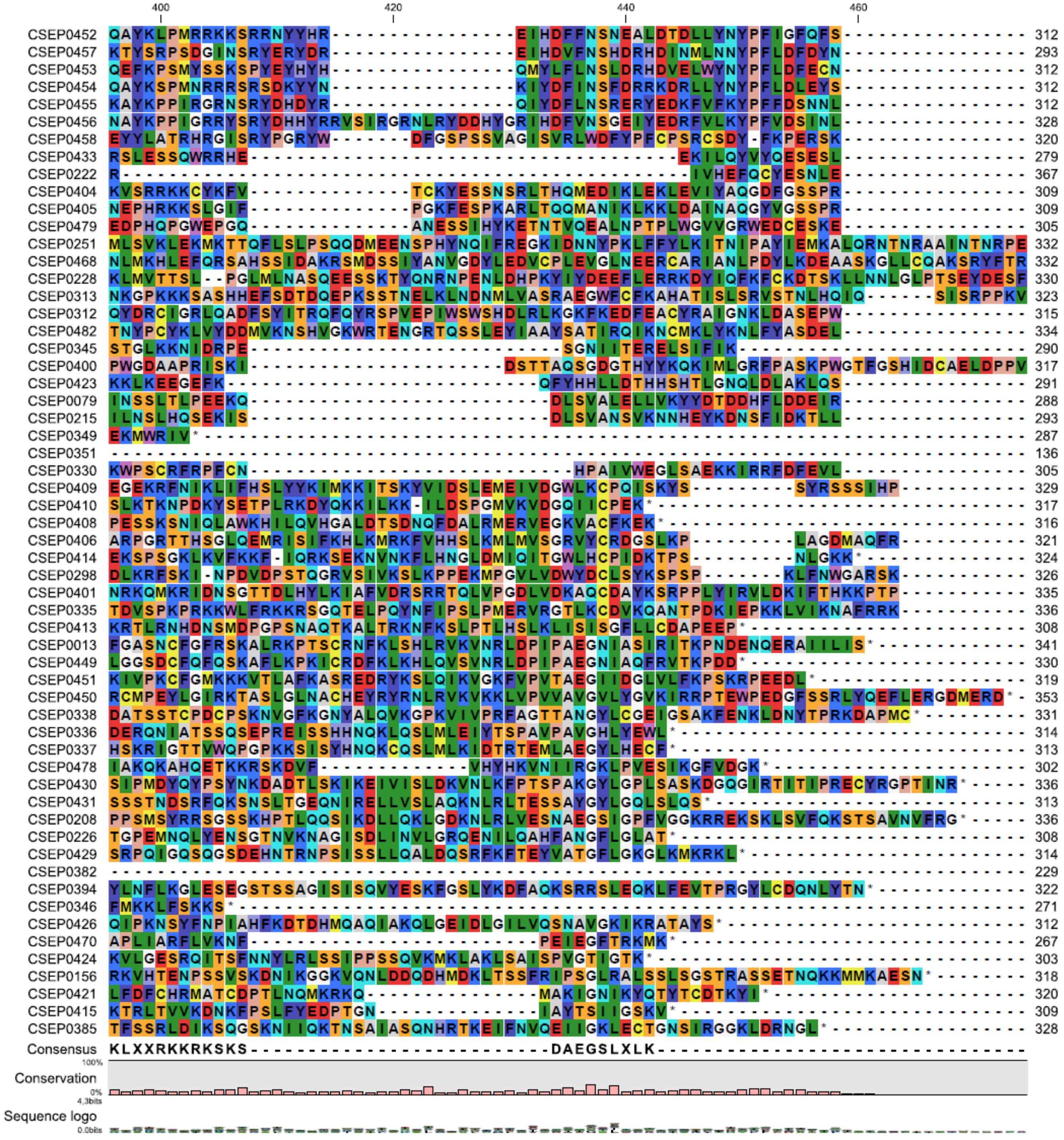

Additional file 14

CSEP family 1 (continued).

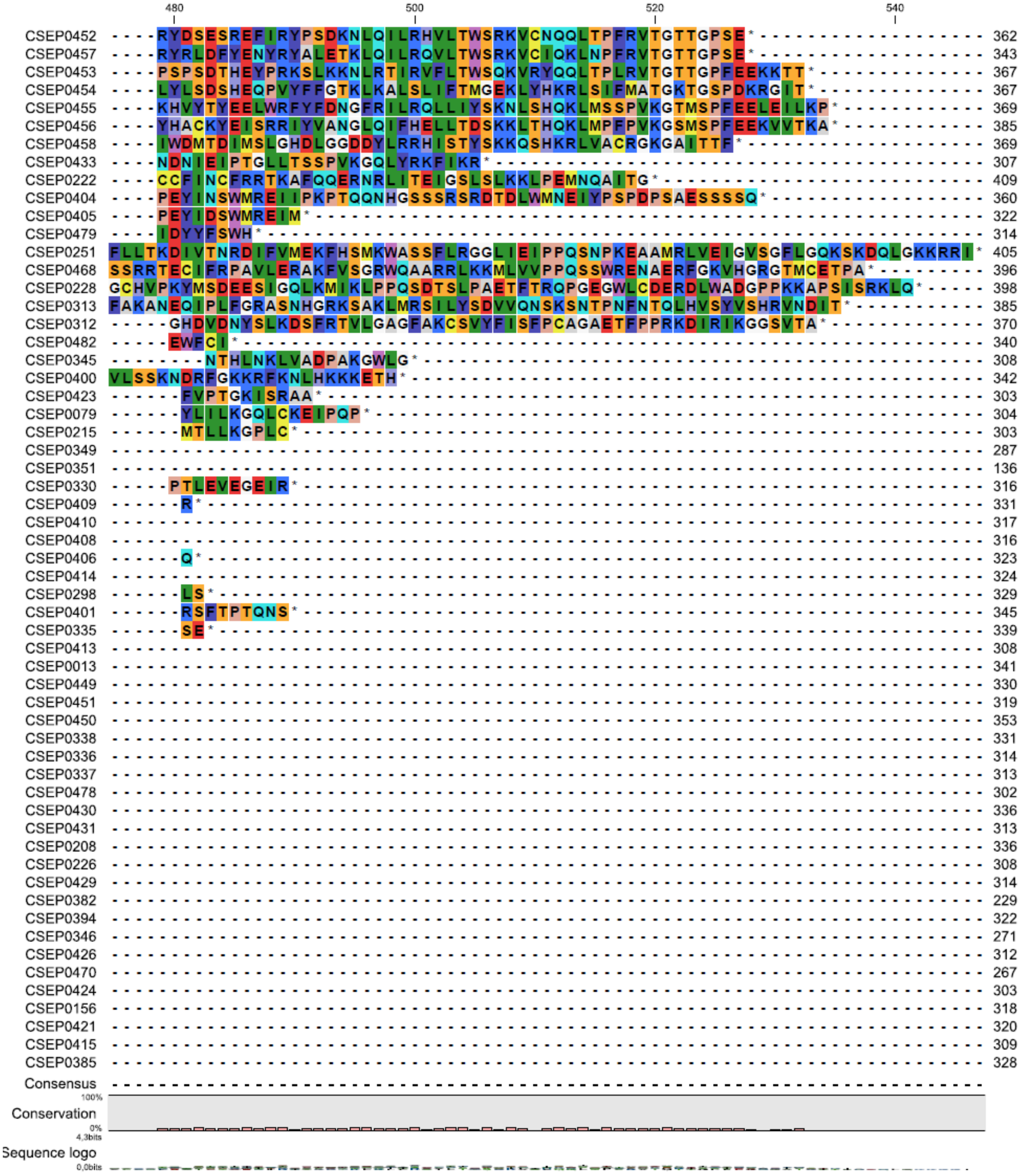

Additional file 14

CSEP family 2.

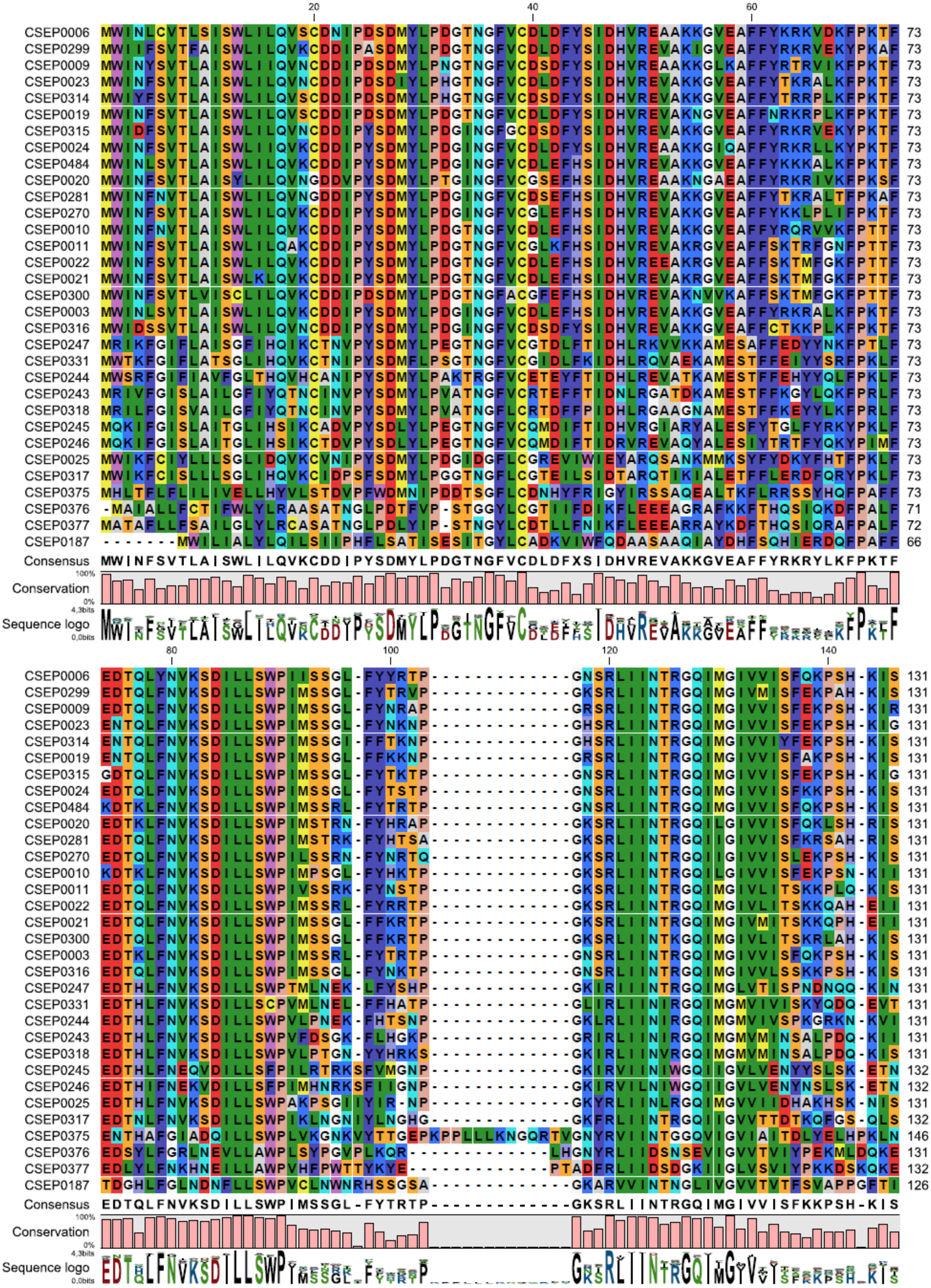

Additional file 14

CSEP family 2 (continued).

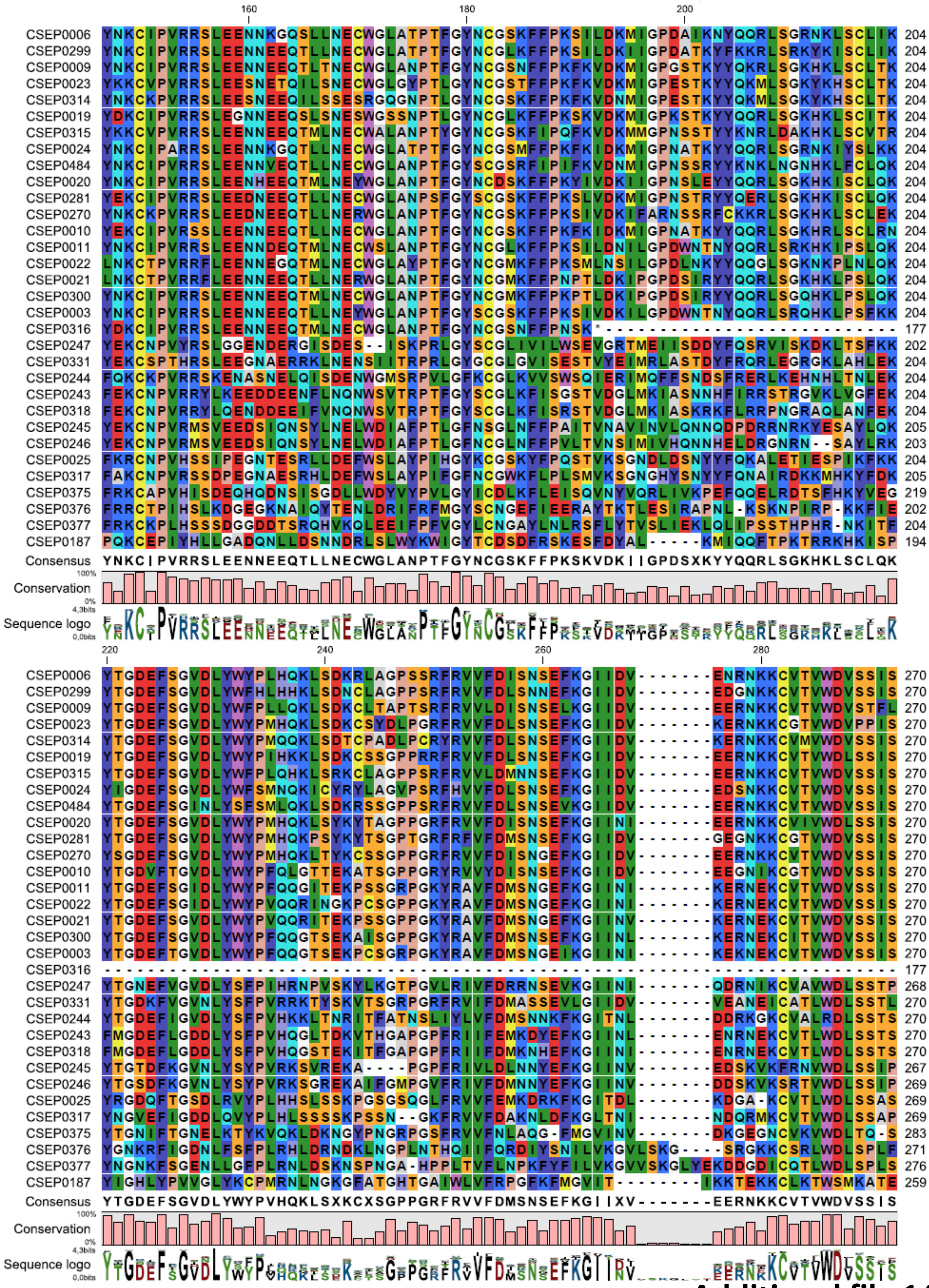

Additional file 14

CSEP family 2 (continued).

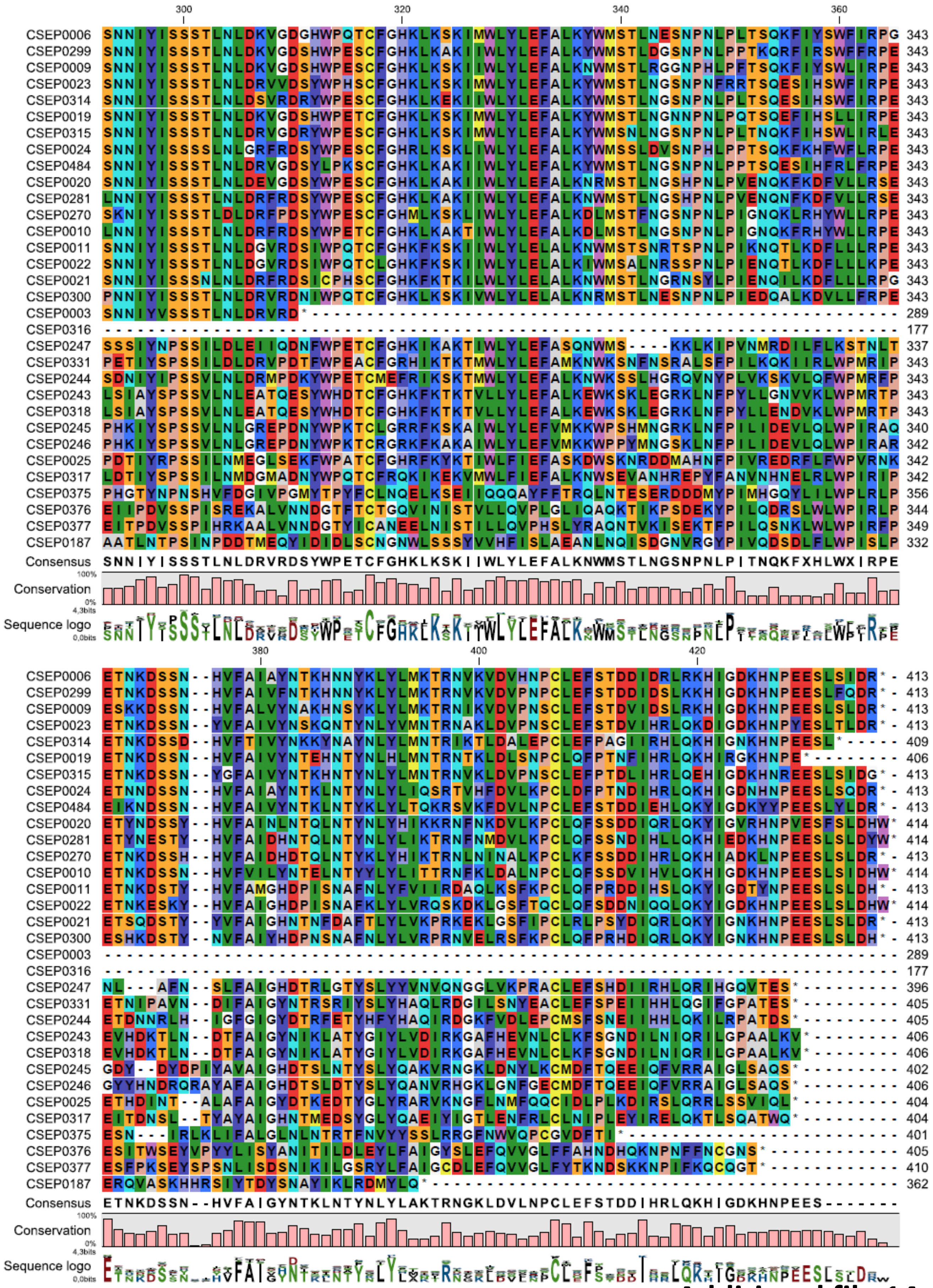

Additional file 14

CSEP family 3.

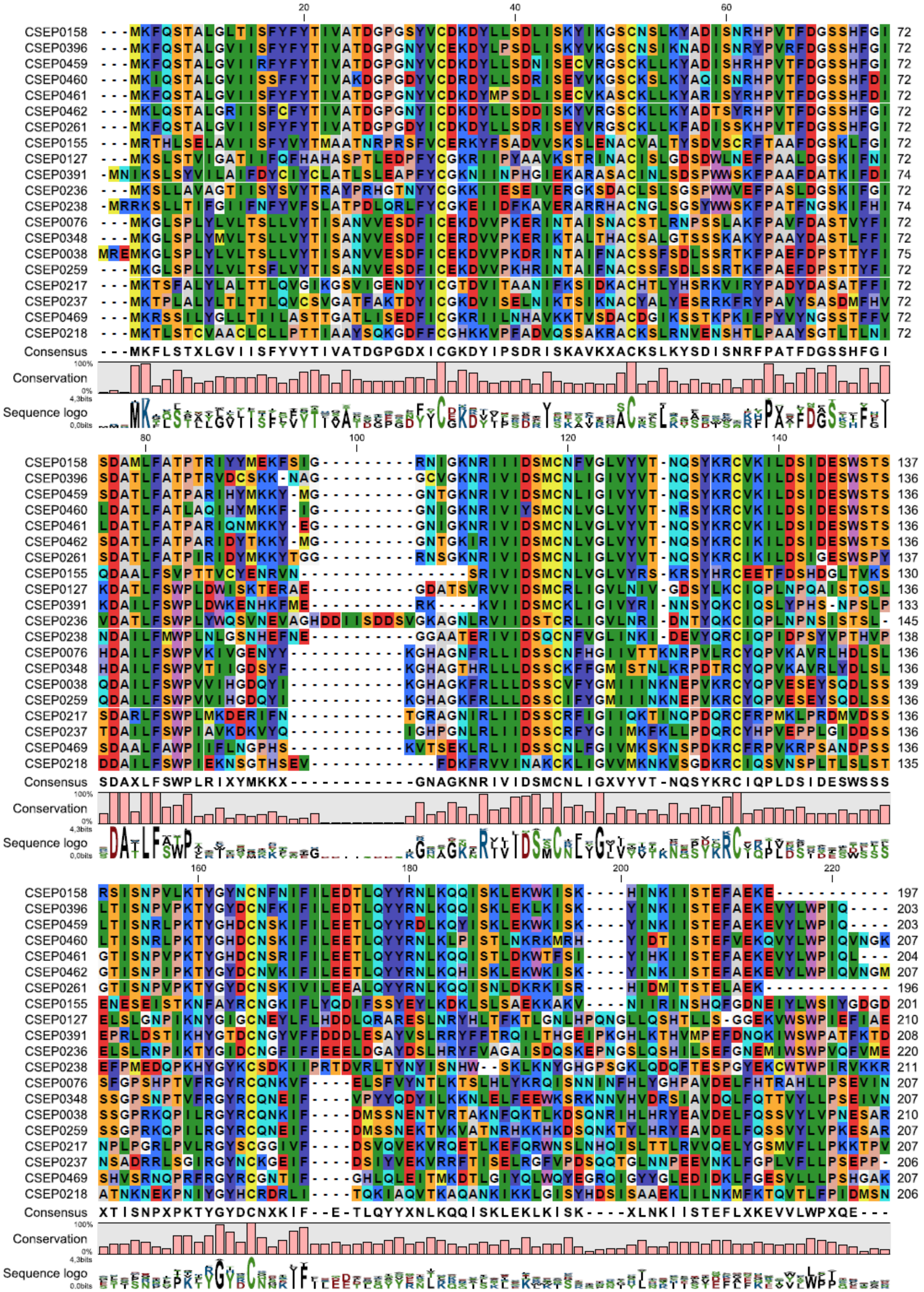

Additional file 14

CSEP family 3 (continued).

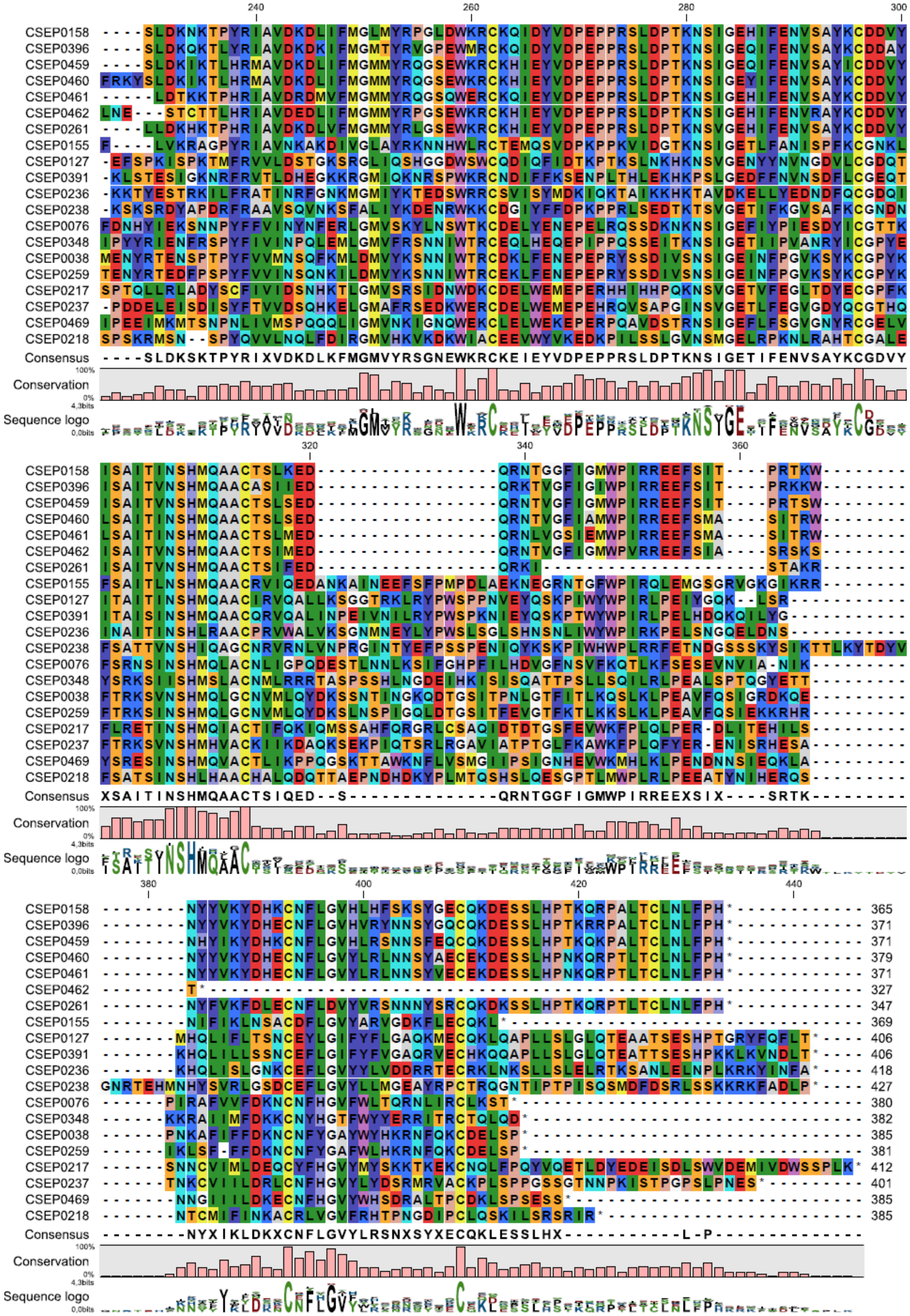

Additional file 14

CSEP family 4.

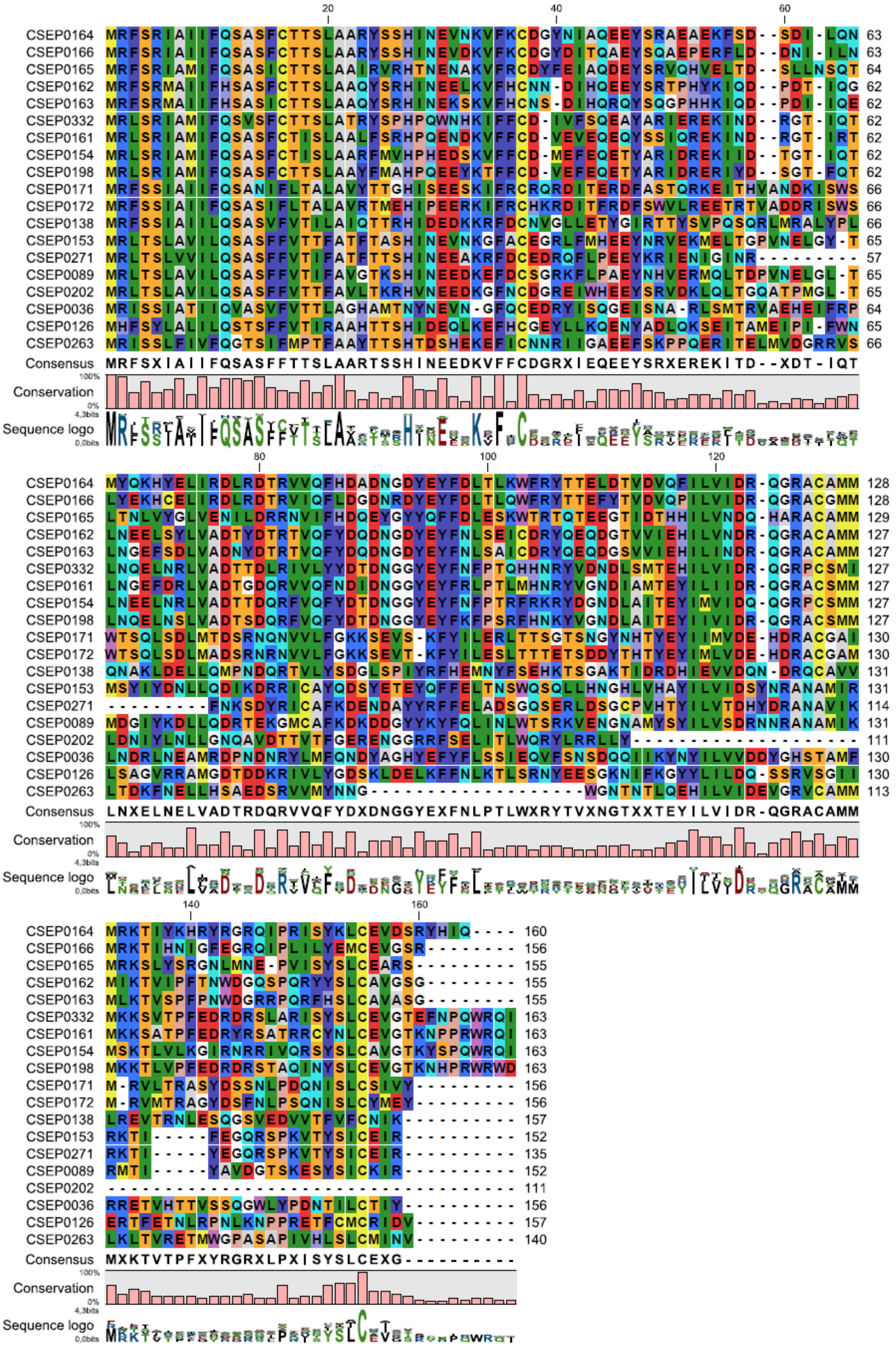

Additional file 14

CSEP family 5.

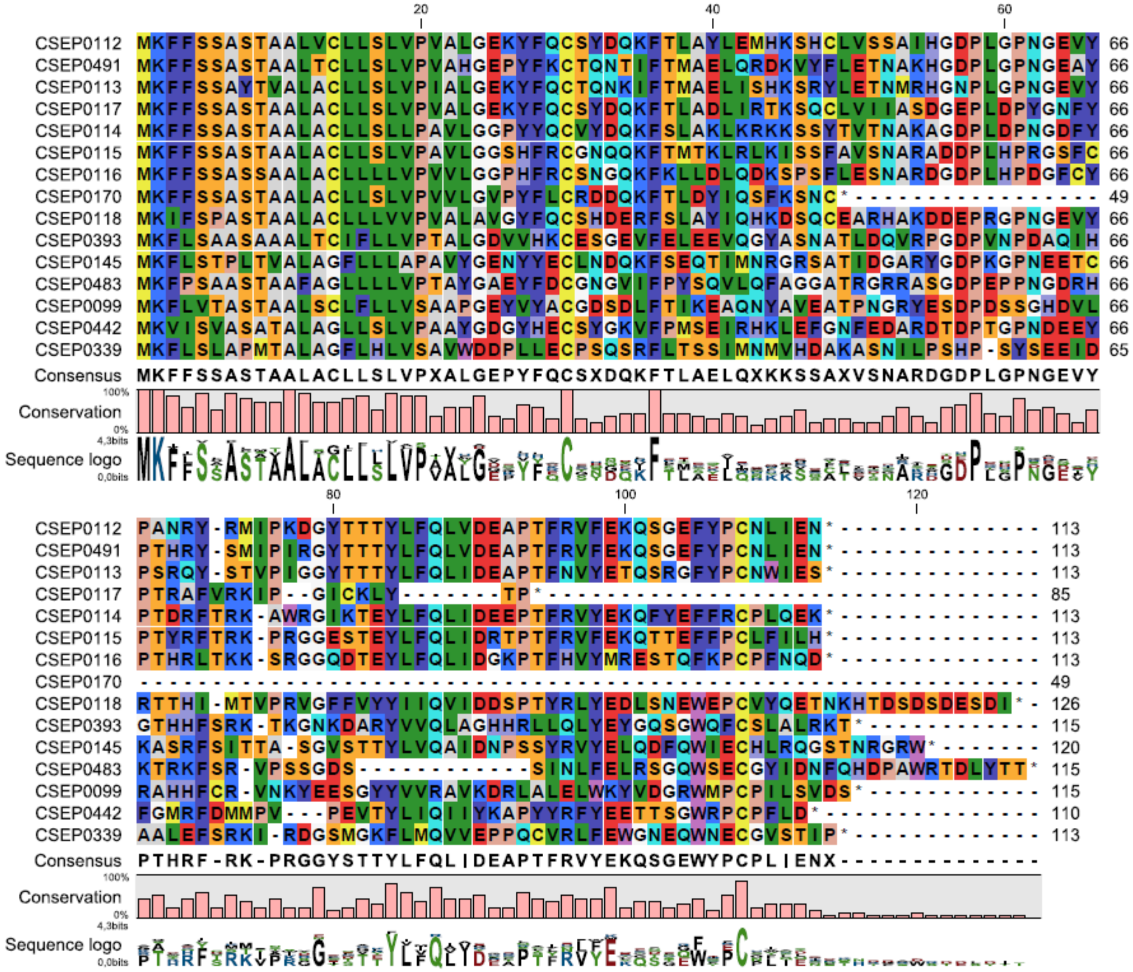

Additional file 14

CSEP family 6.

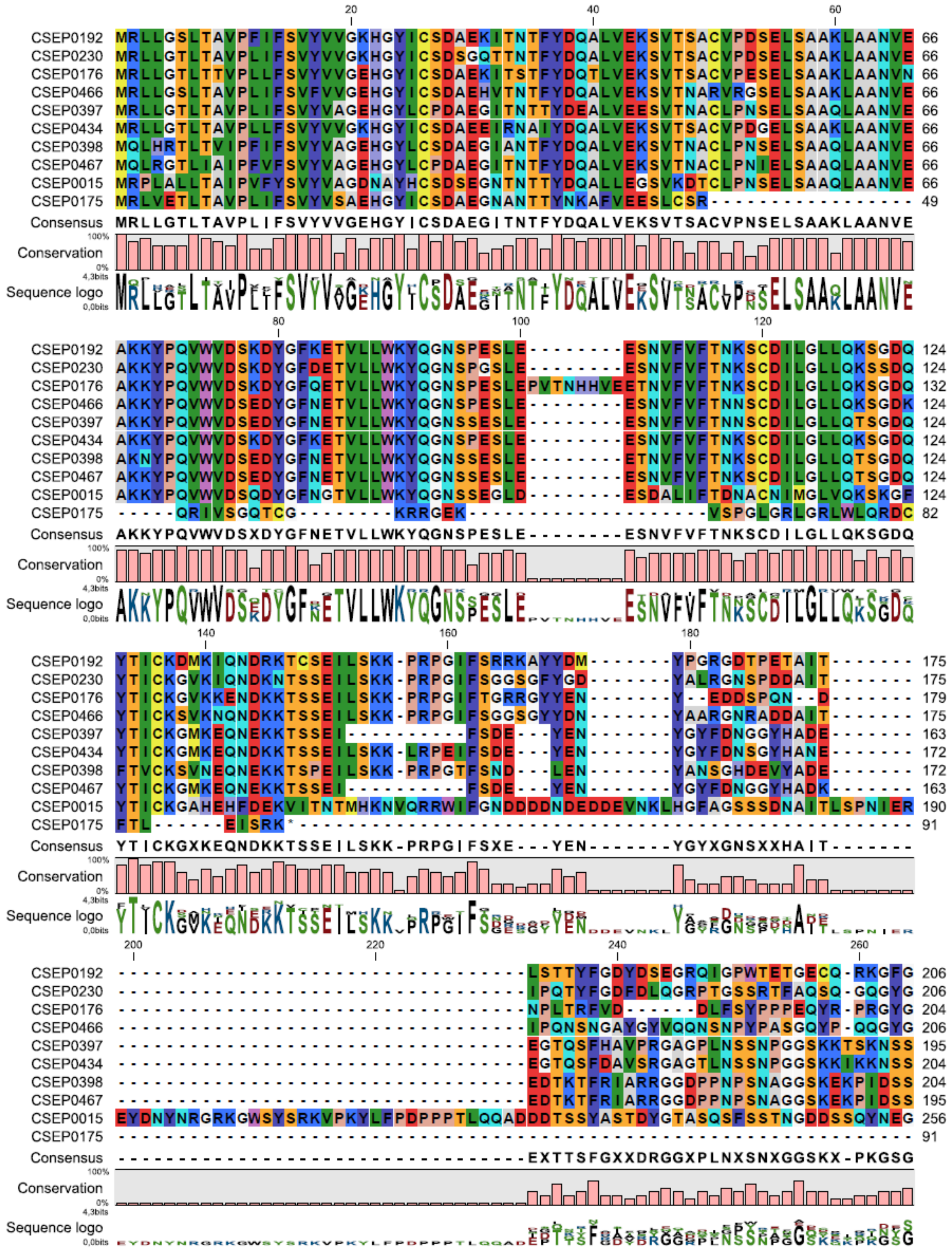

Additional file 14

CSEP family 6 (continued).

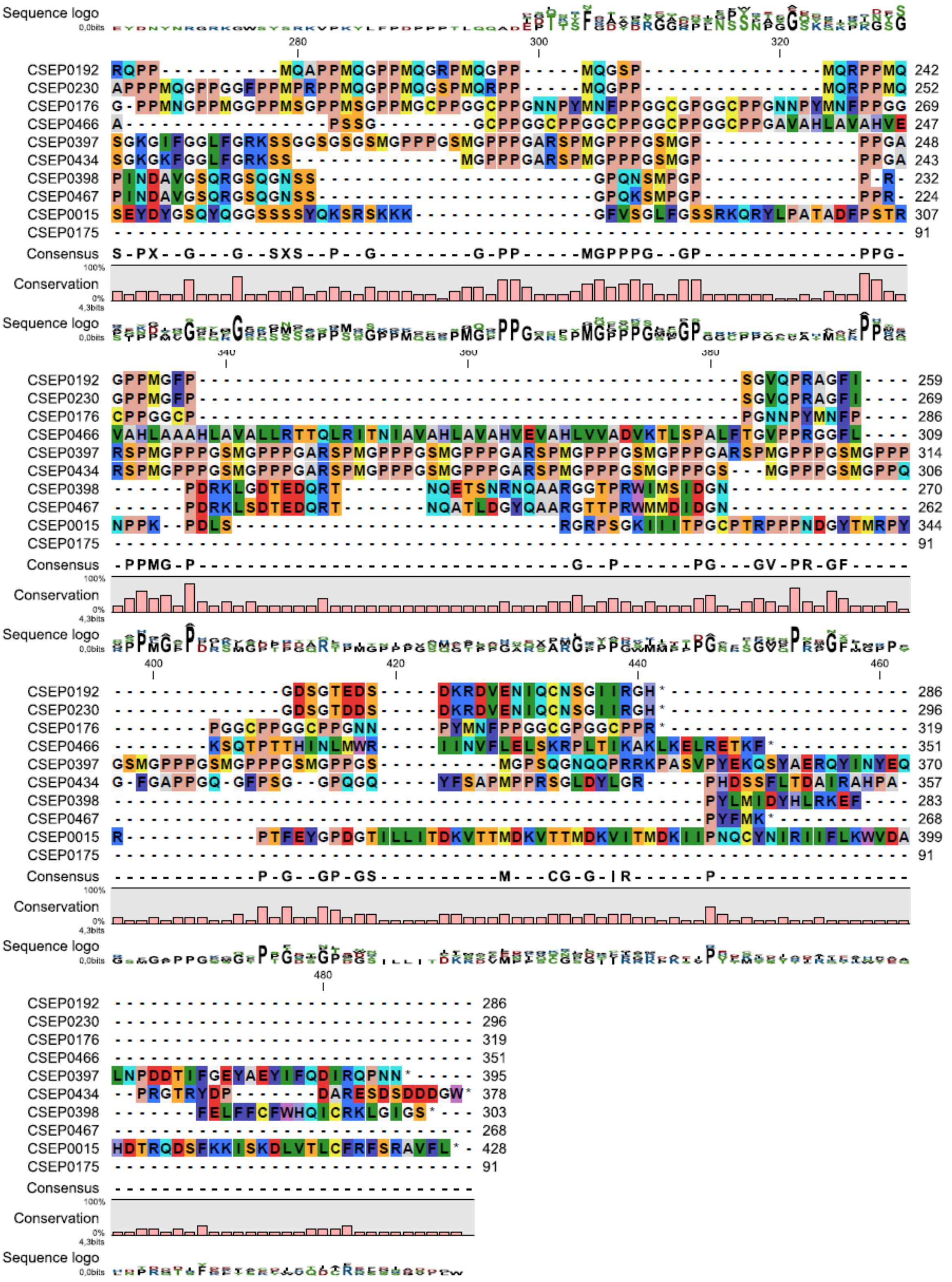

Additional file 14

CSEP family 7.

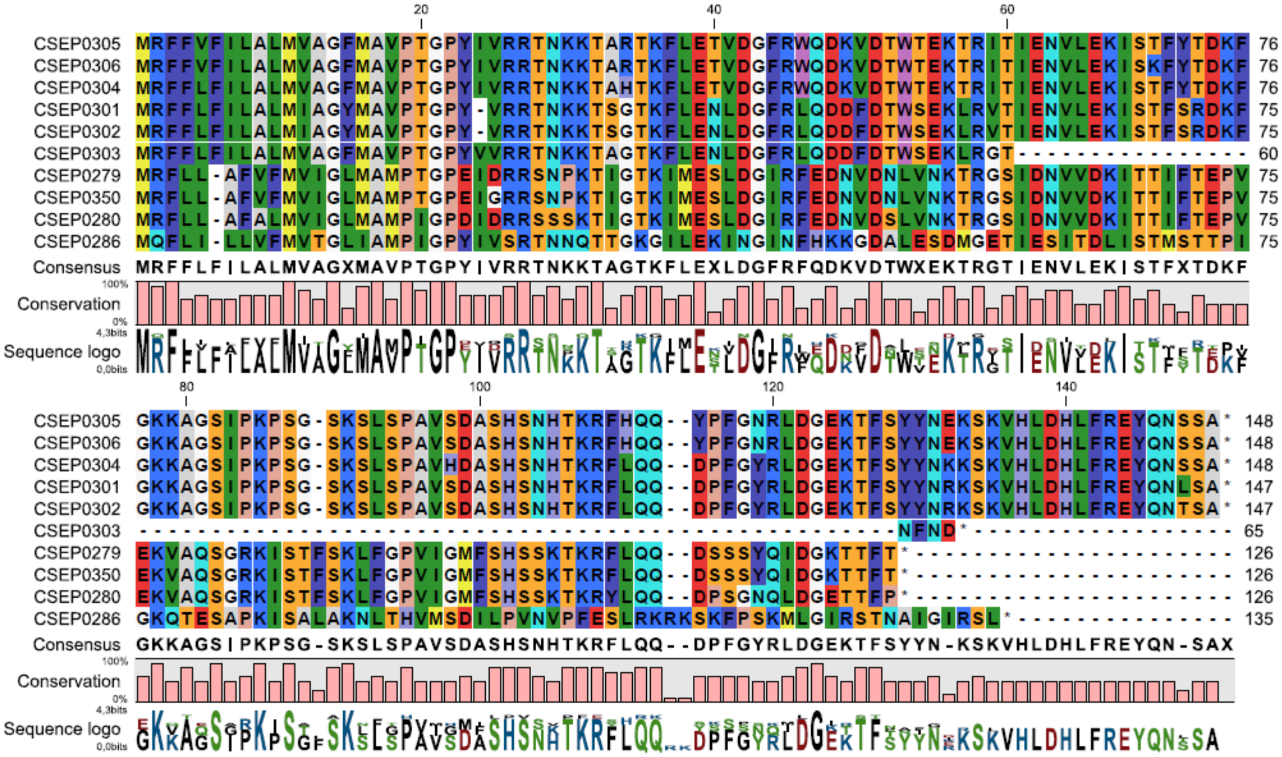

CSEP family 8.

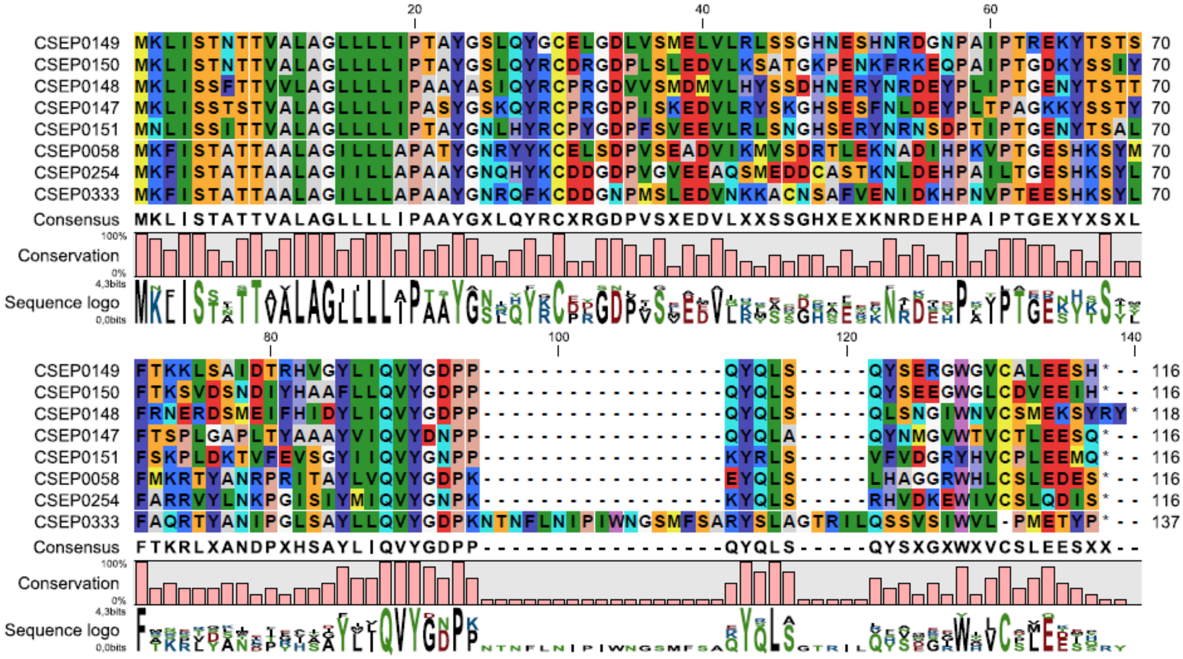

Additional file 14

CSEP family 9.

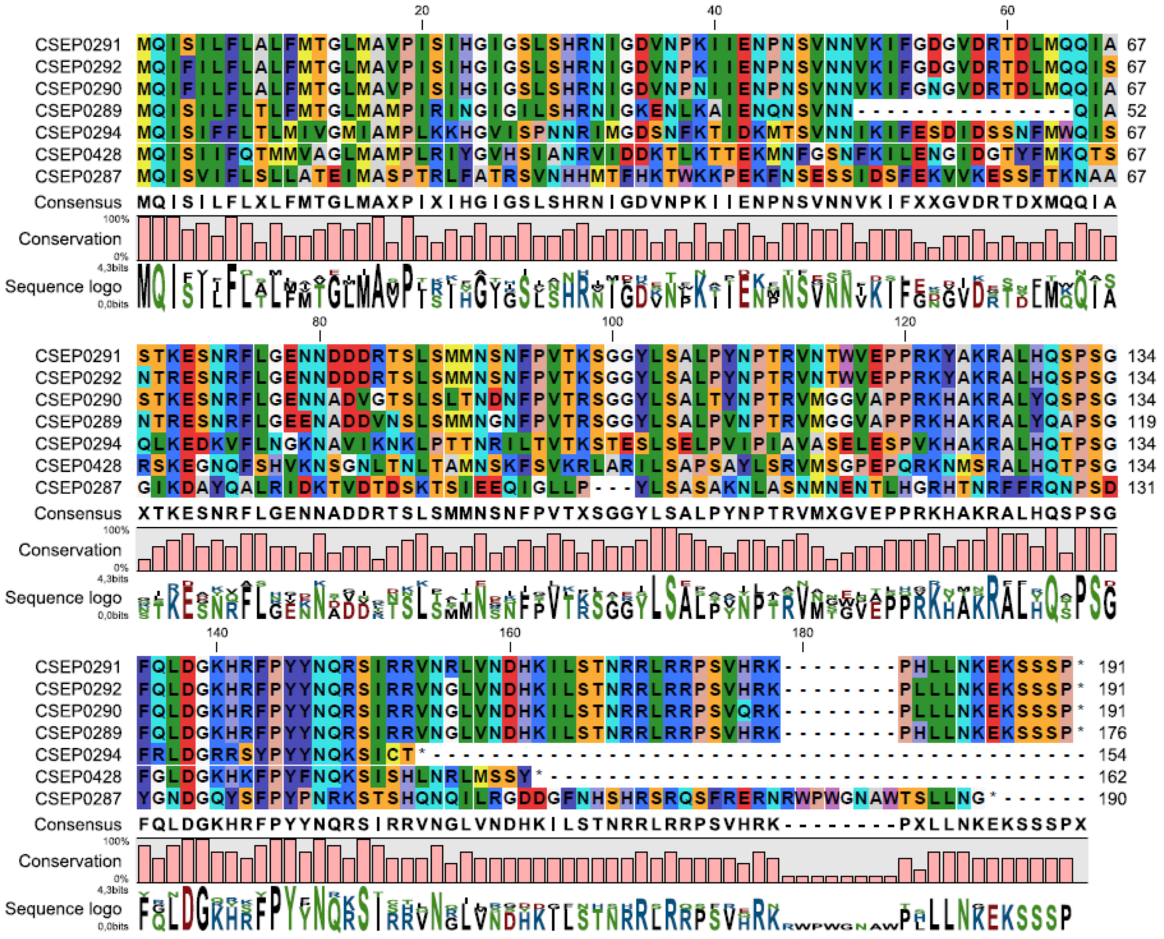

CSEP family 10.

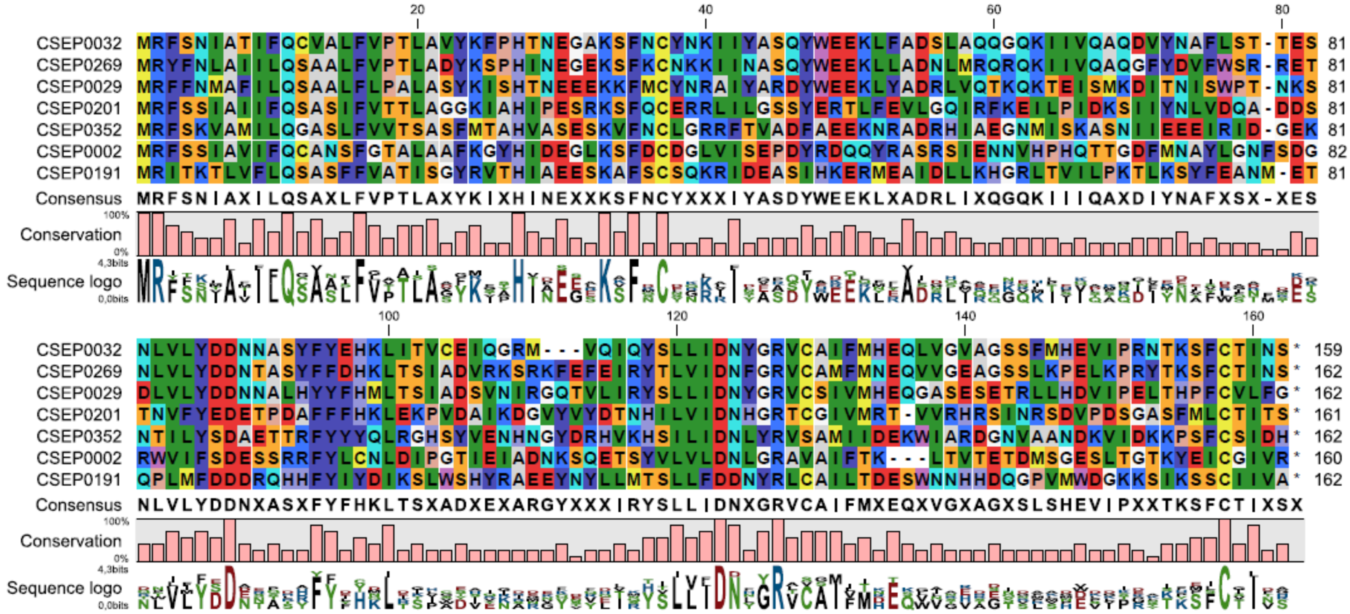

Additional file 14

CSEP family 11.

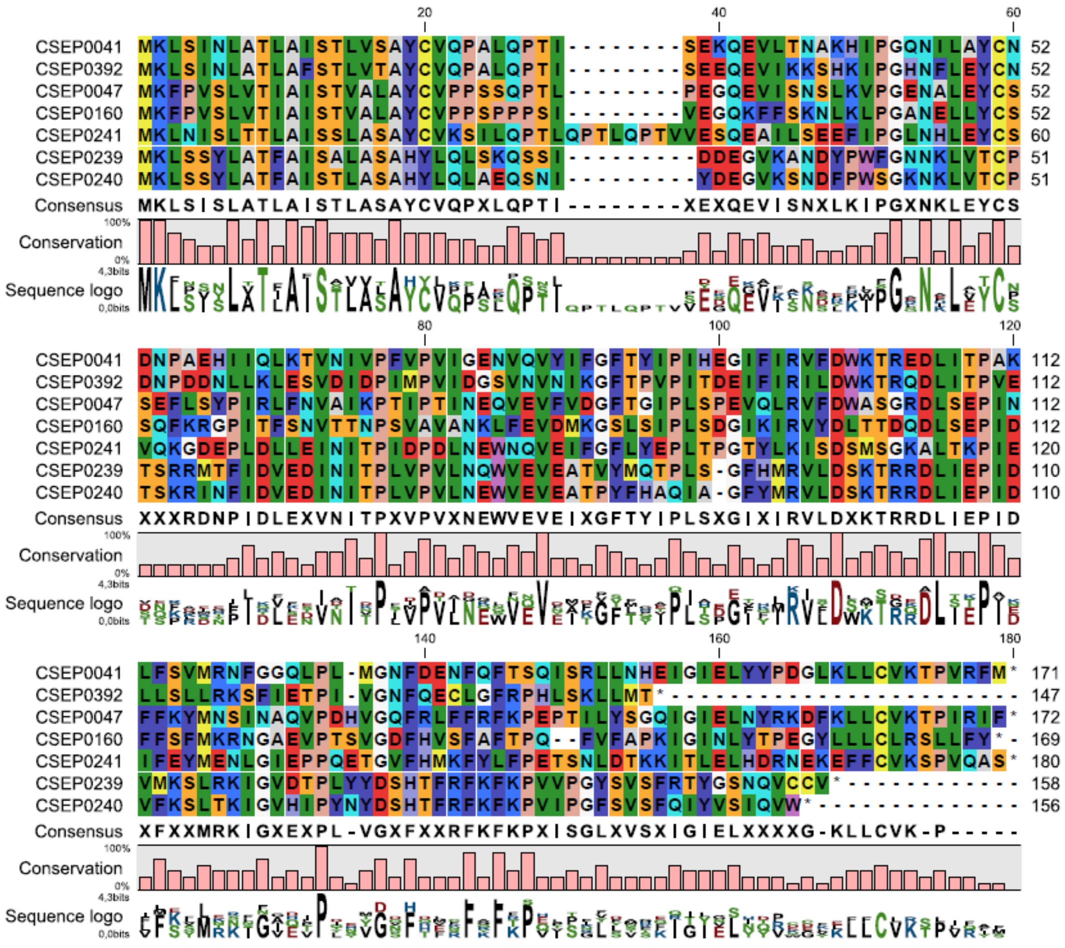

CSEP family 12.

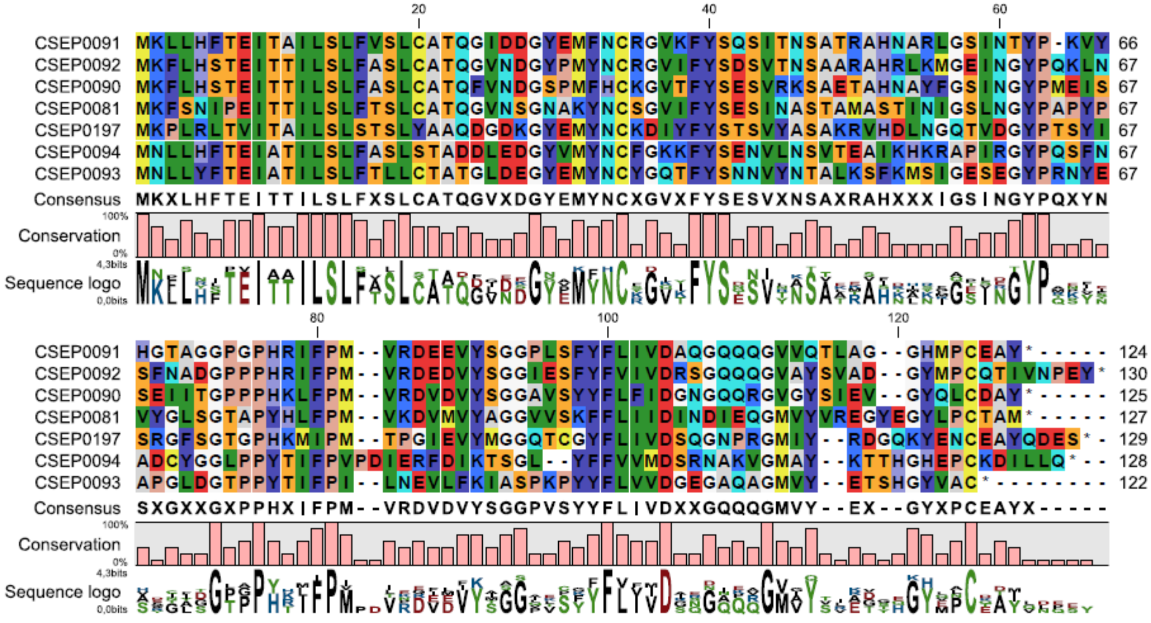

Additional file 14

CSEP family 13.

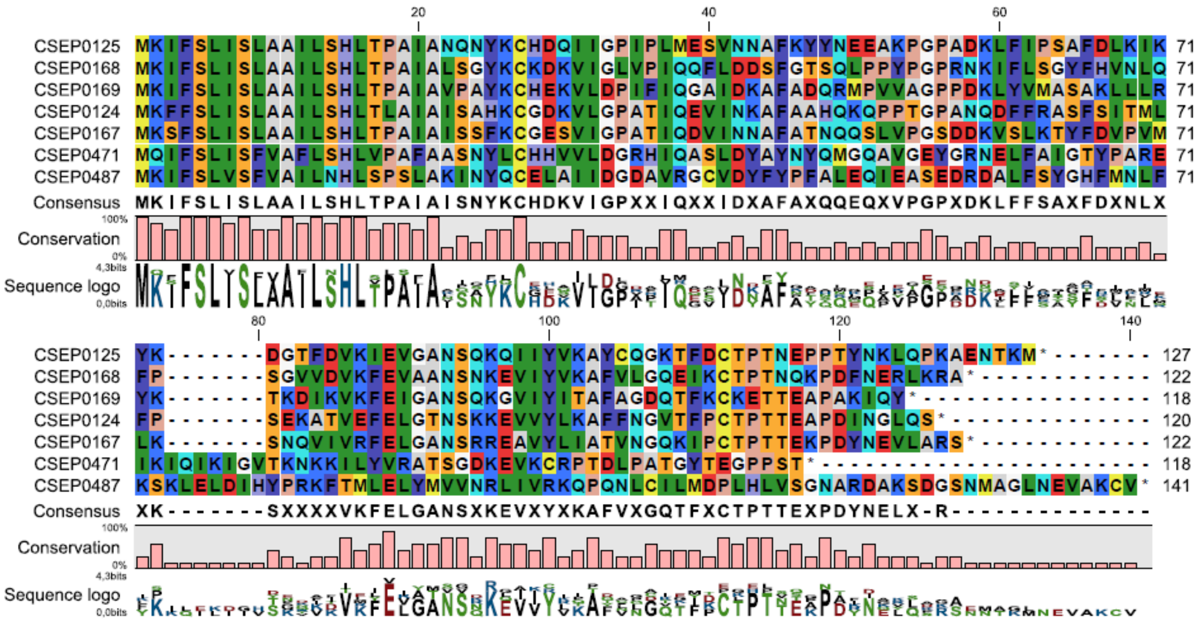

CSEP family 14.

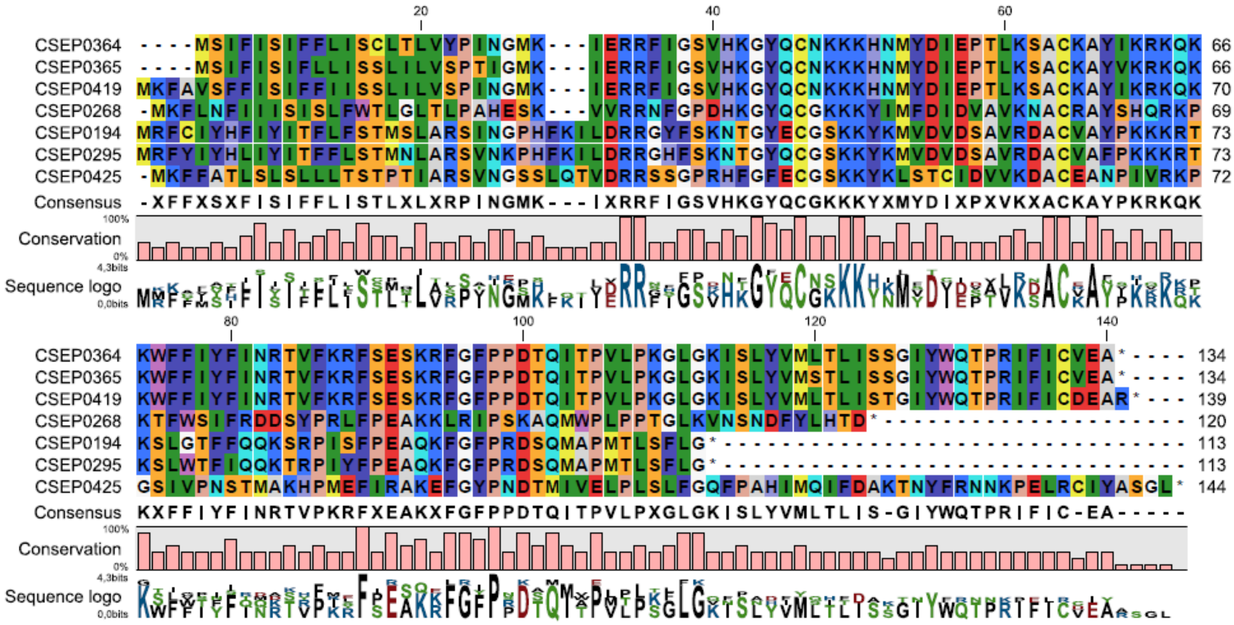

Additional file 14

CSEP family 15.

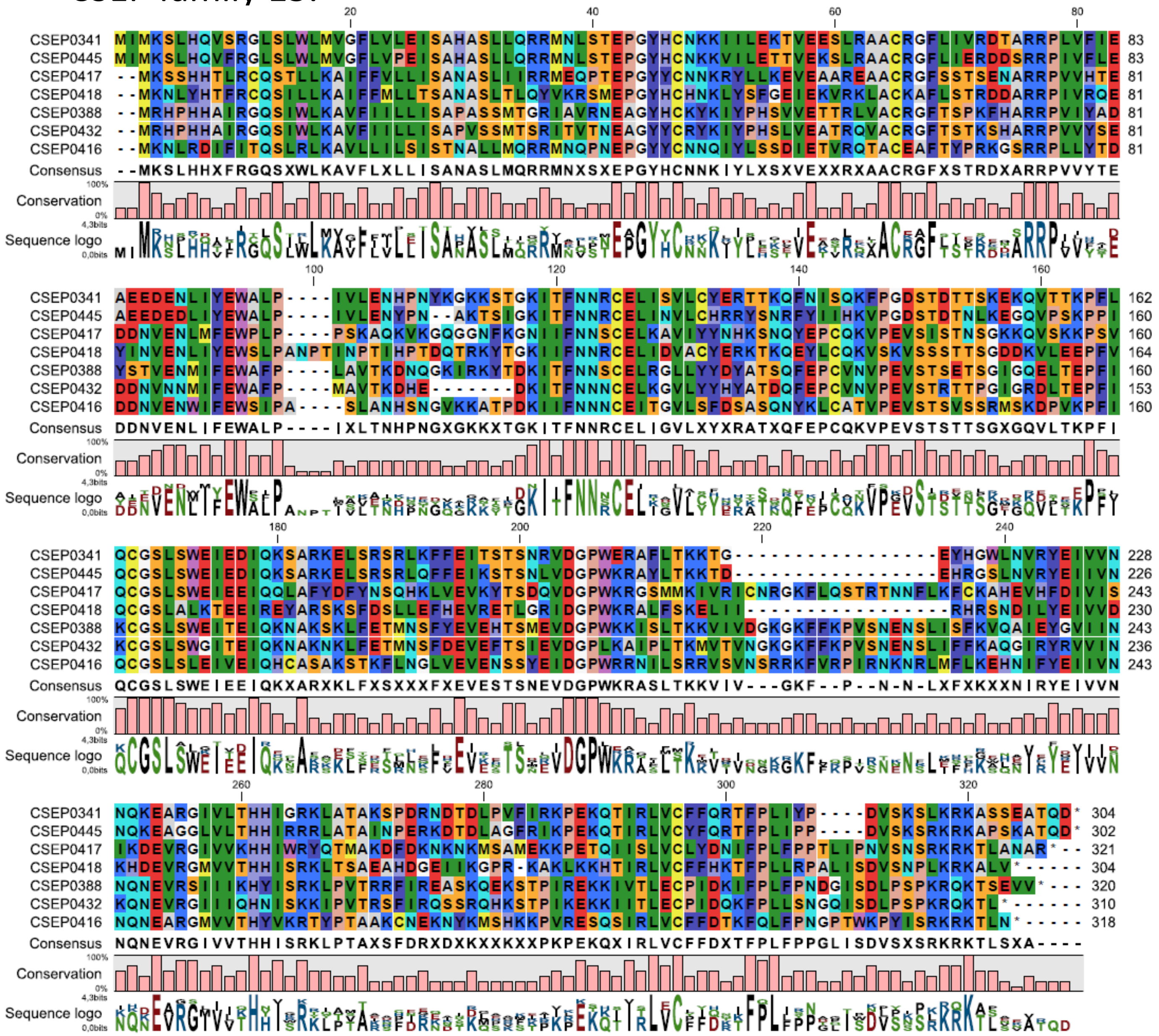

CSEP family 16.

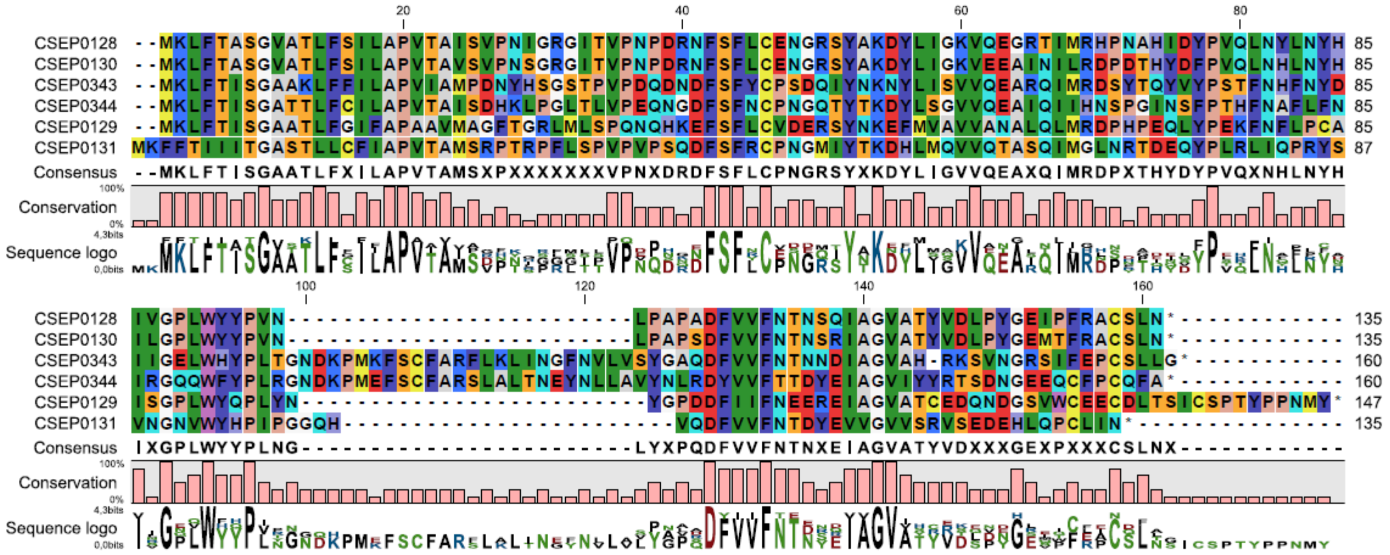

Additional file 14

CSEP family 17.

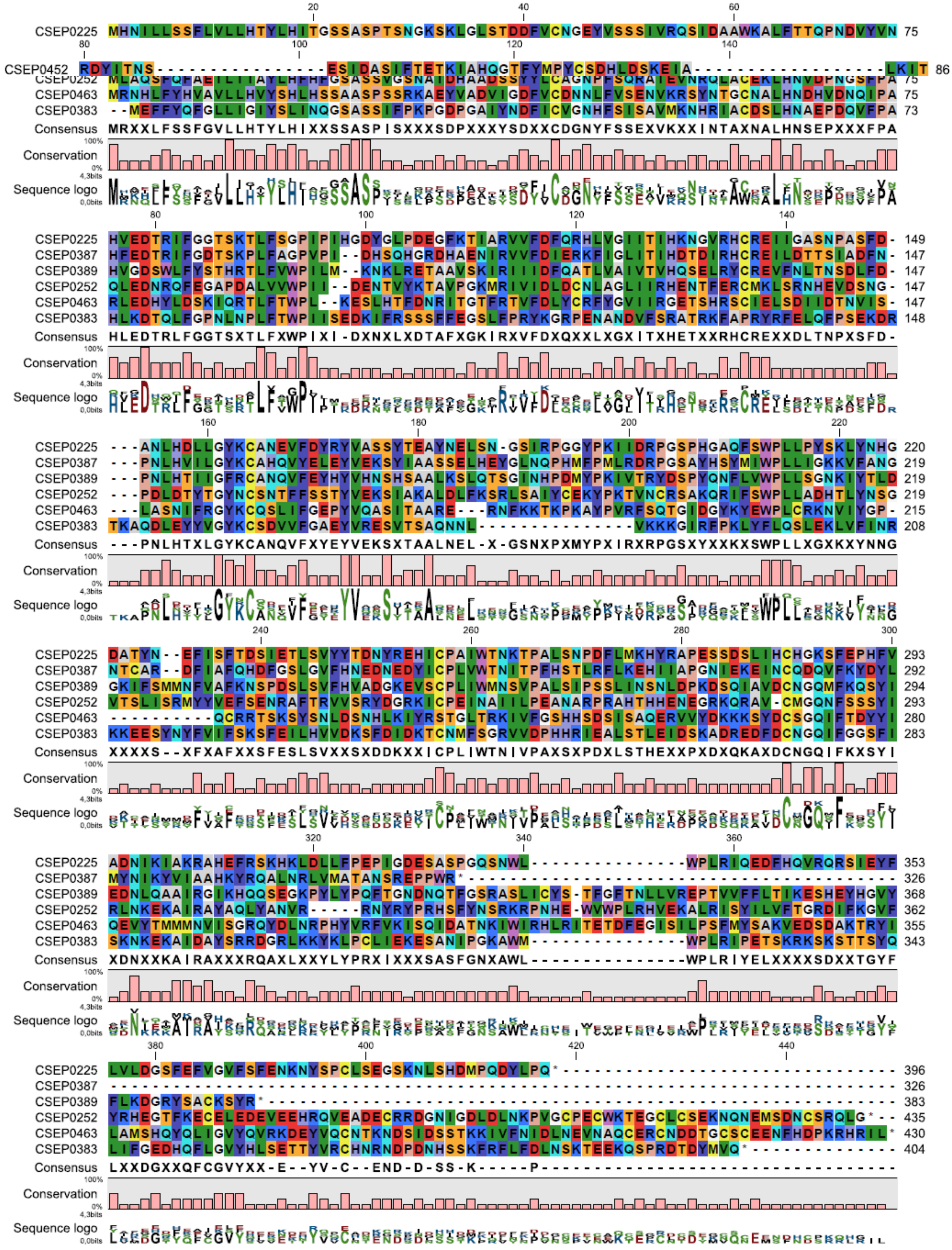

Additional file 14

CSEP family 18.

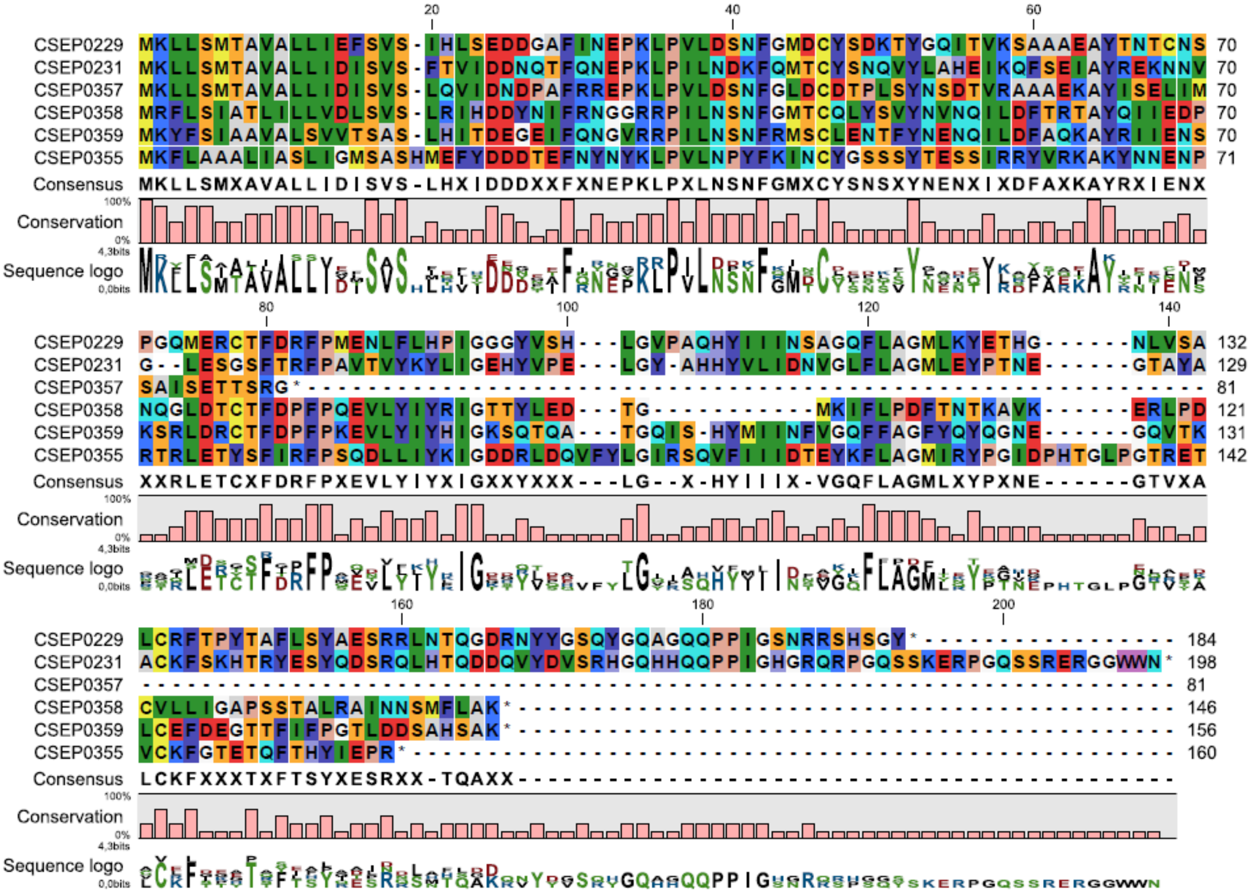

CSEP family 19.

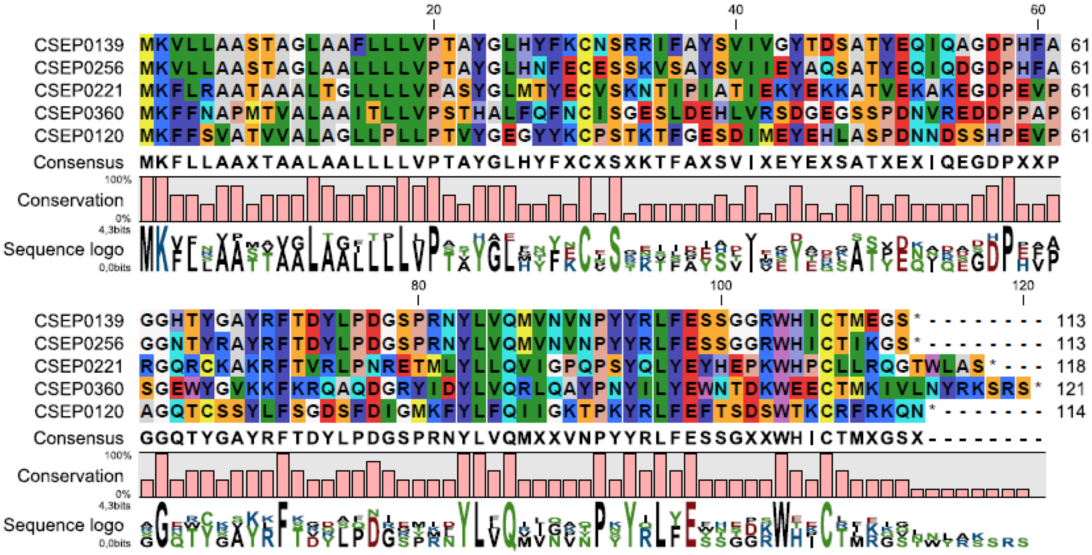

Additional file 14

CSEP family 20.

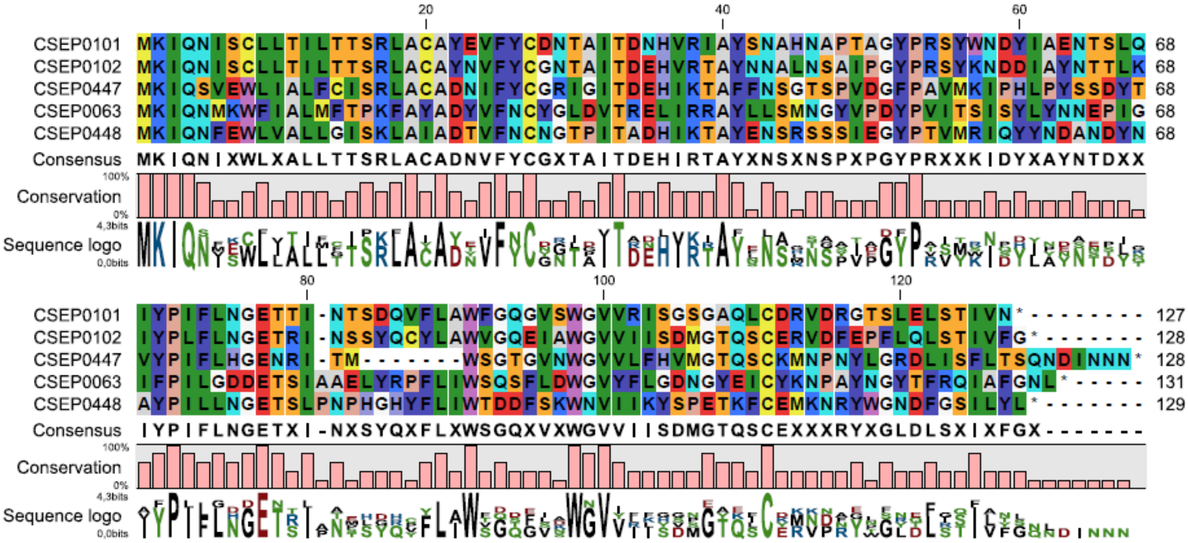

CSEP family 21.

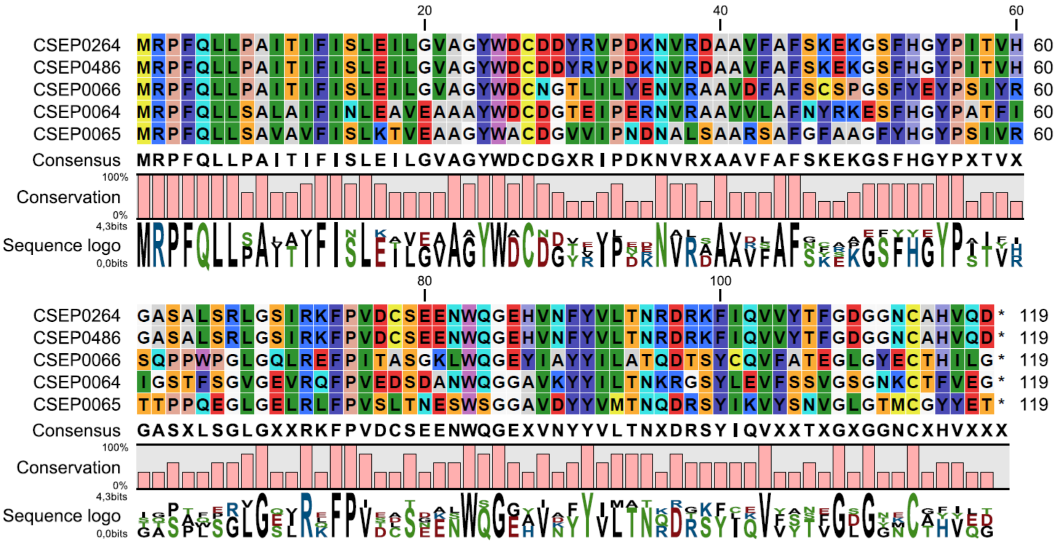

Additional file 14

CSEP family 22.

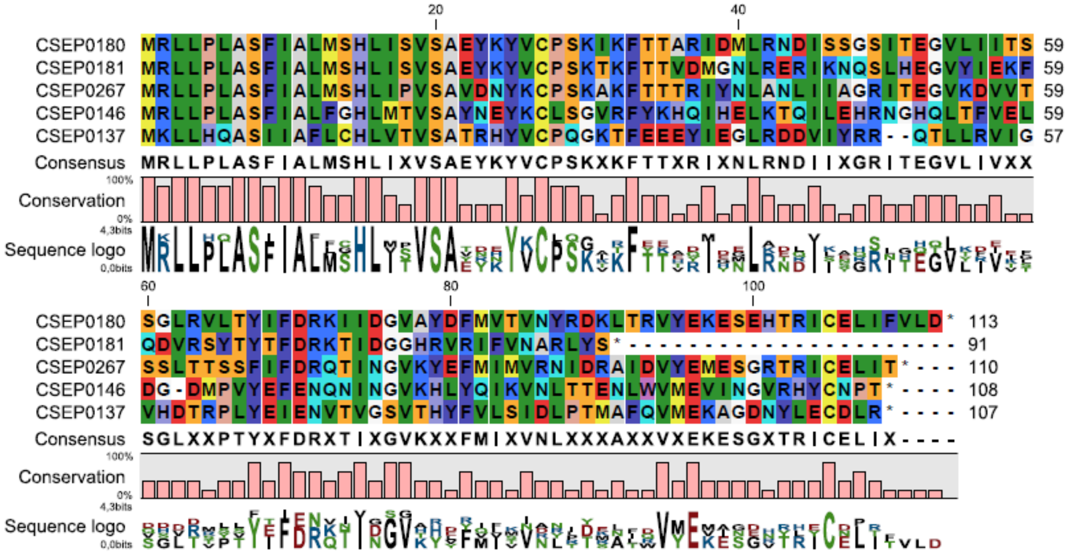

CSEP family 23.

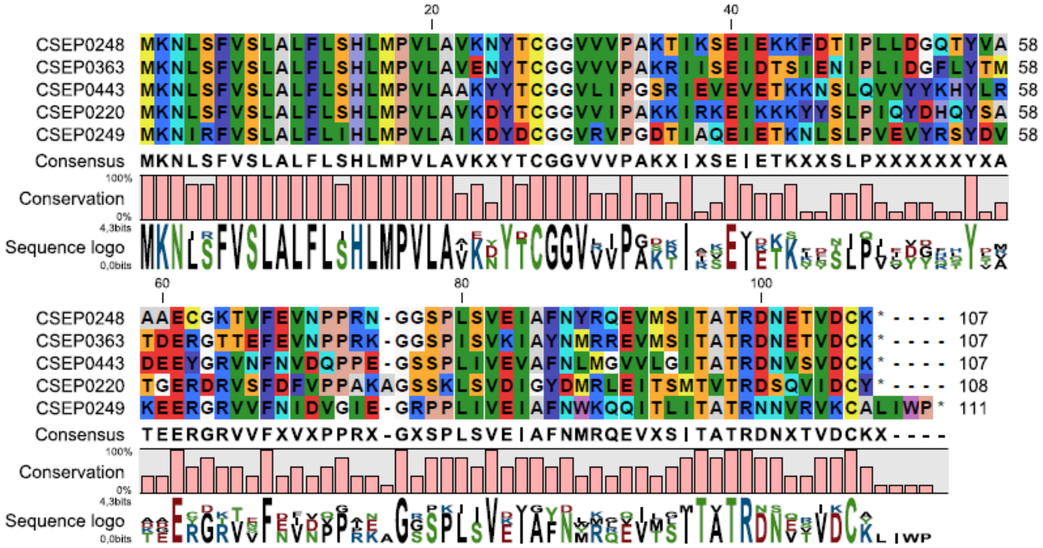

Additional file 14

CSEP family 24.

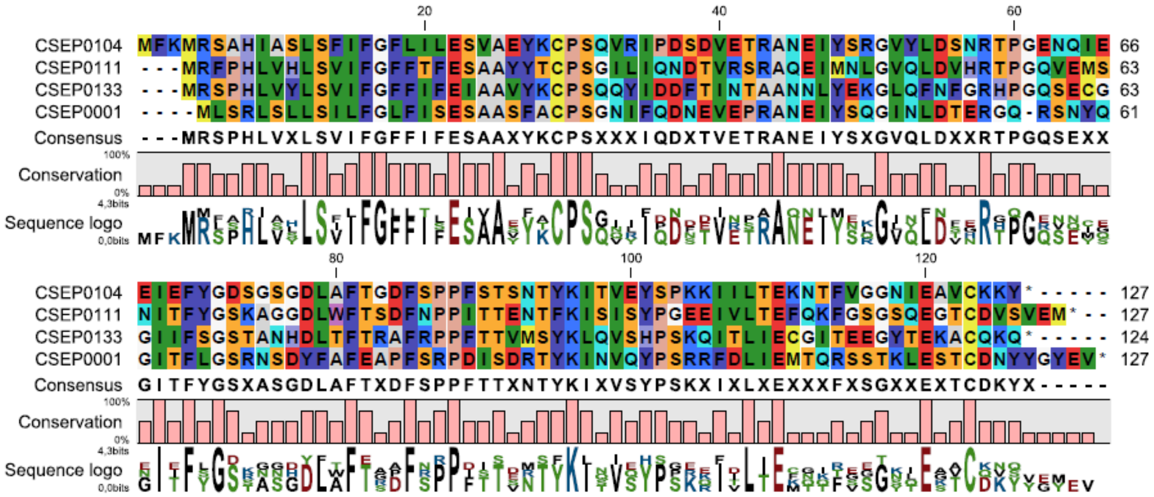

CSEP family 25.

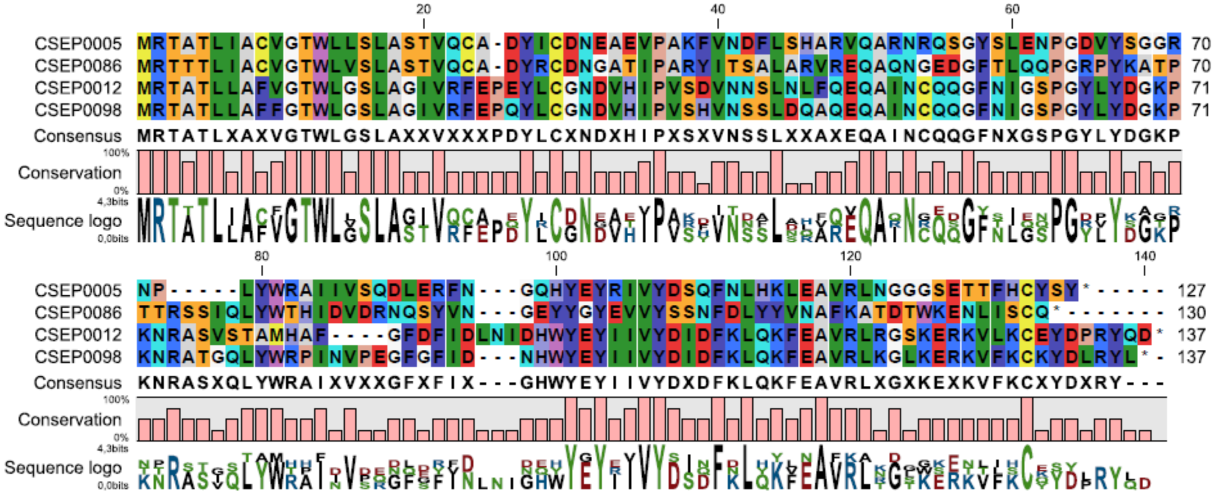

Additional file 14

CSEP family 26.

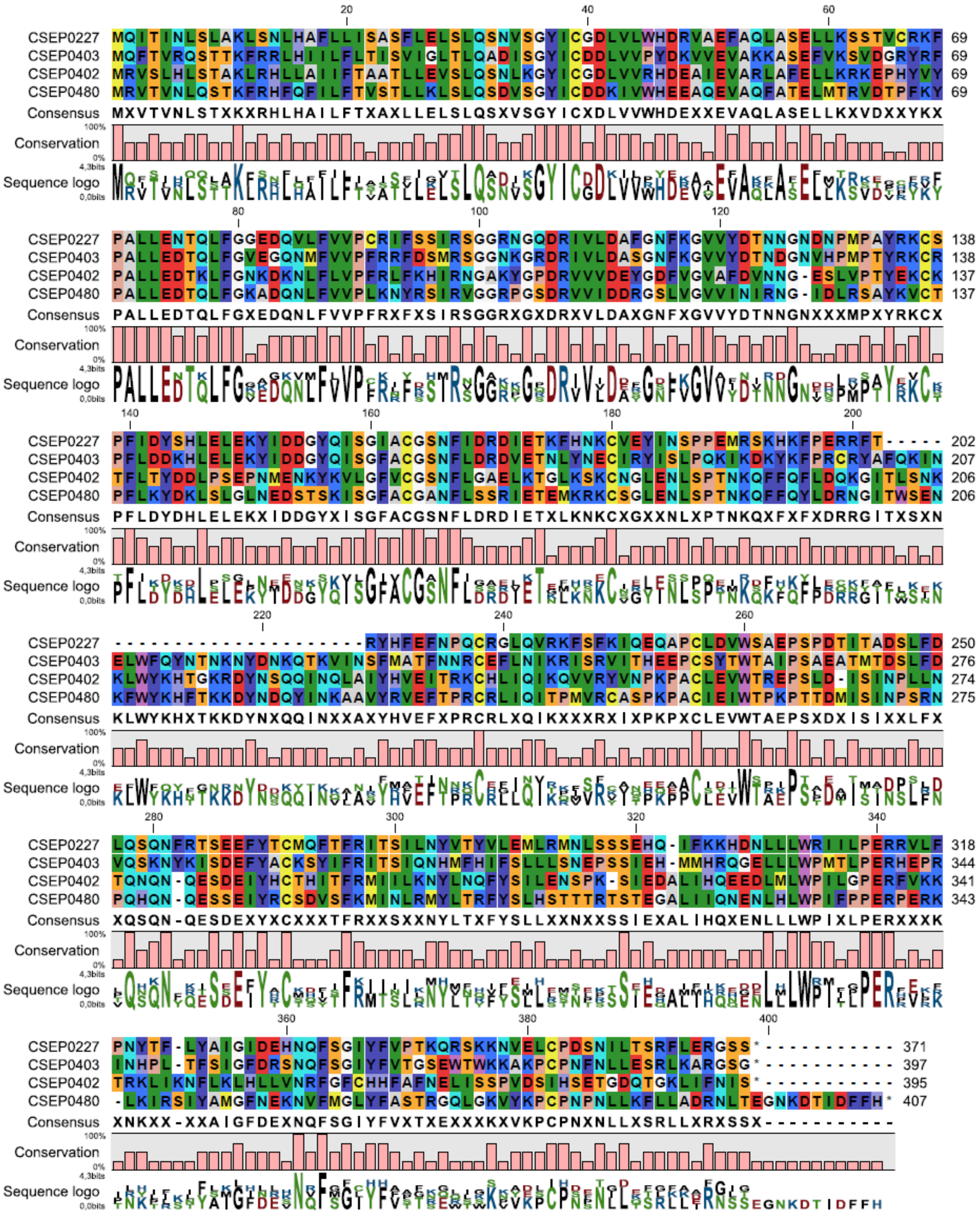

Additional file 14

CSEP family 27.

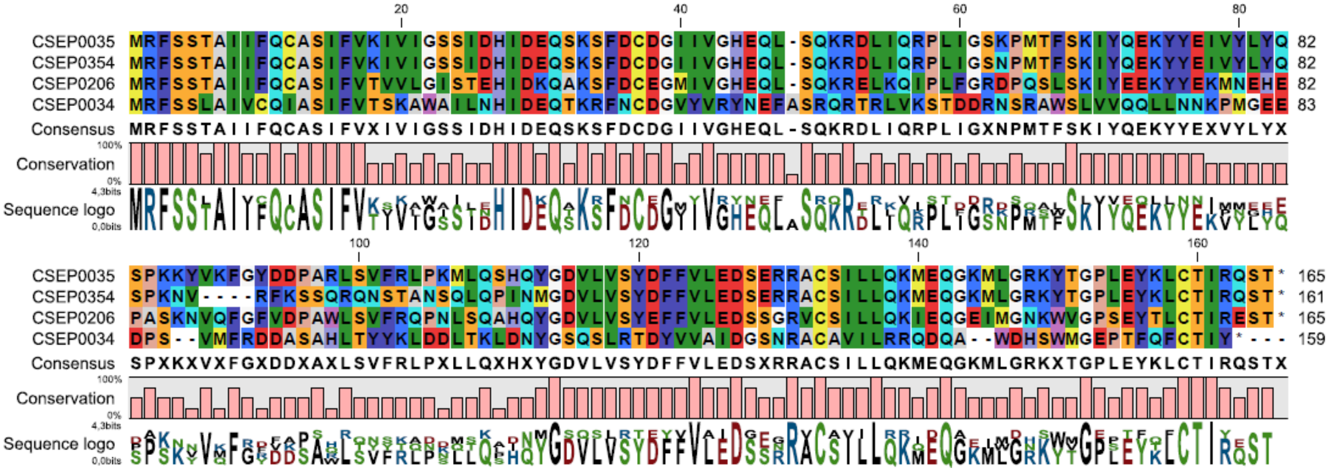

CSEP family 28.

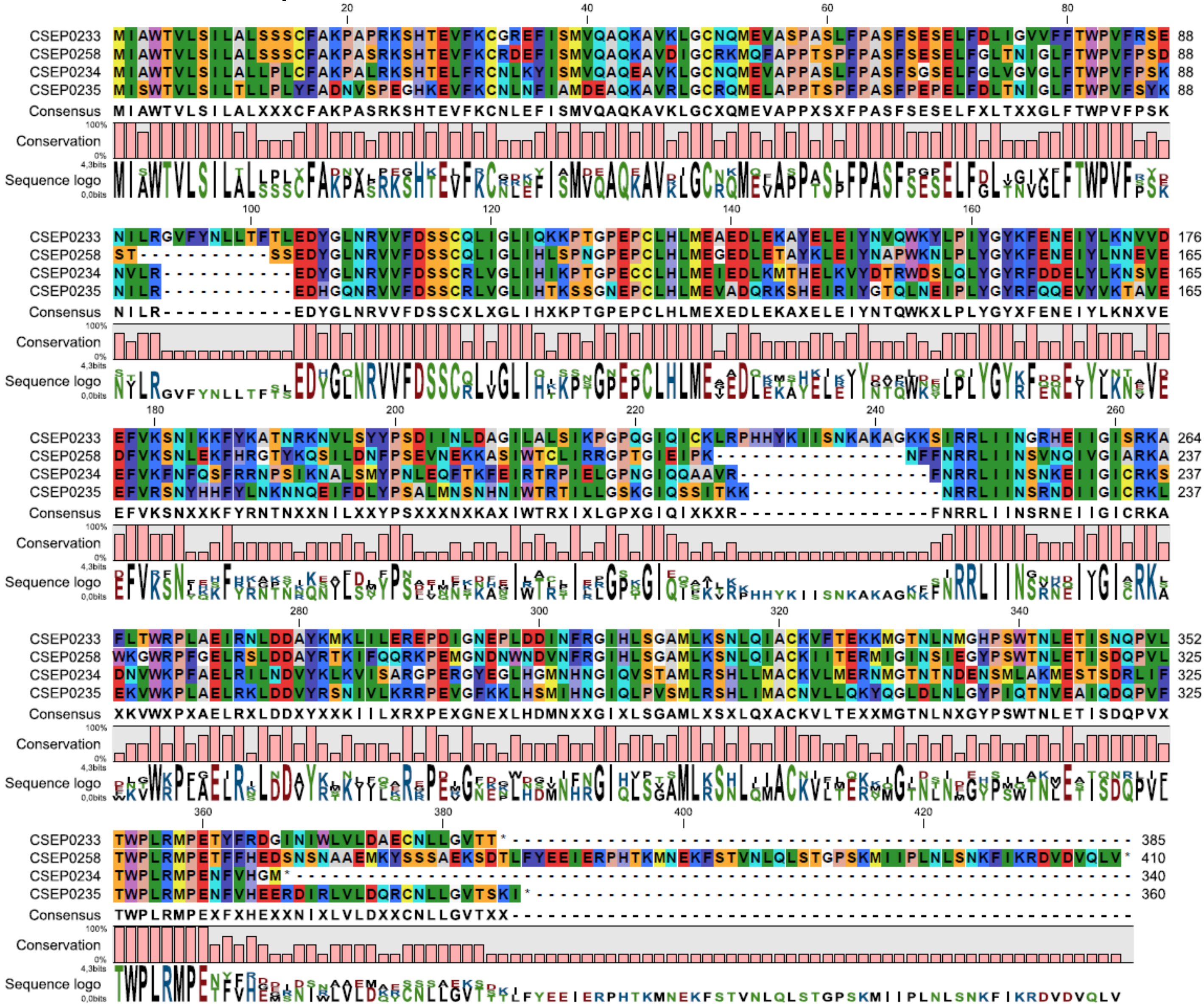

Additional file 14

CSEP family 29.

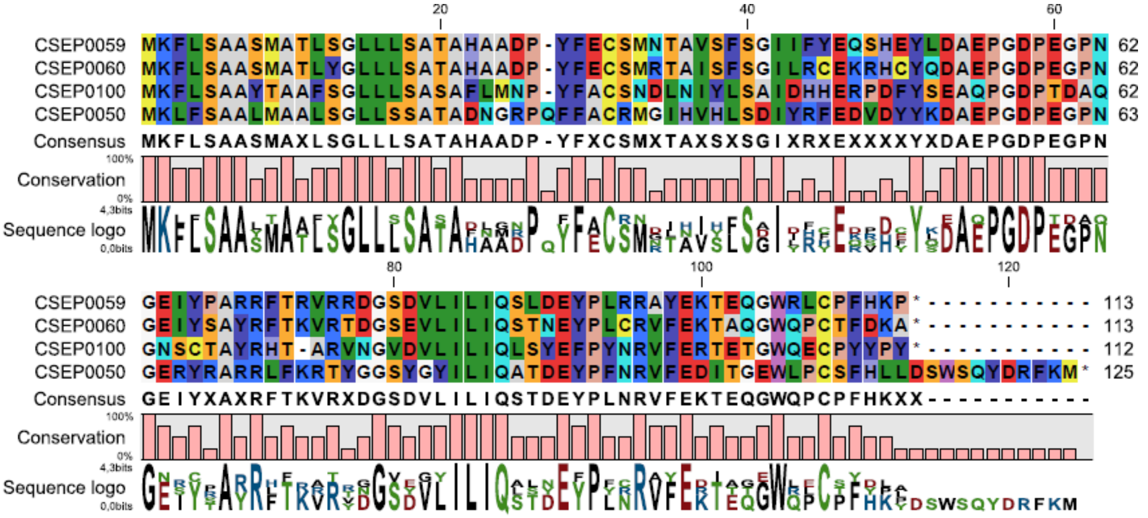

CSEP family 30.

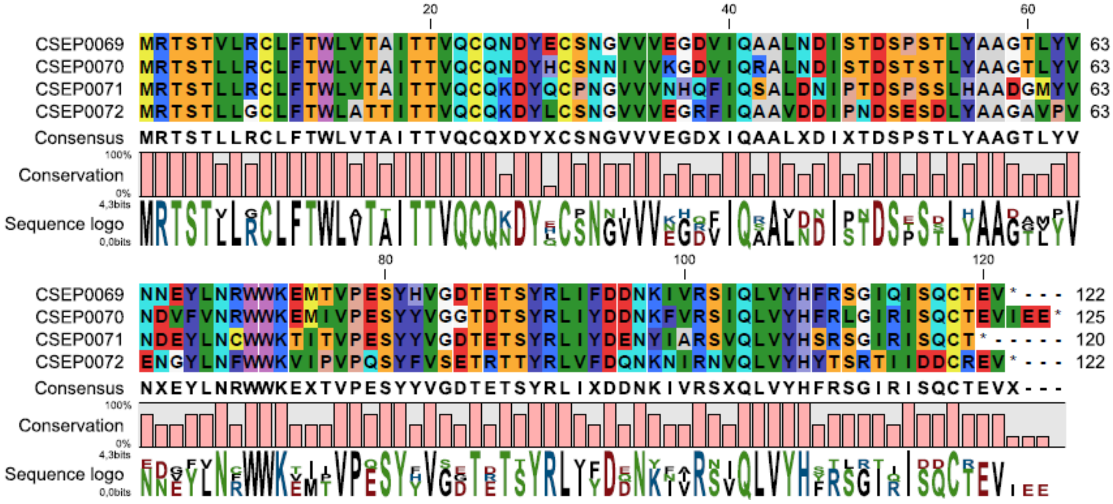

CSEP family 31.

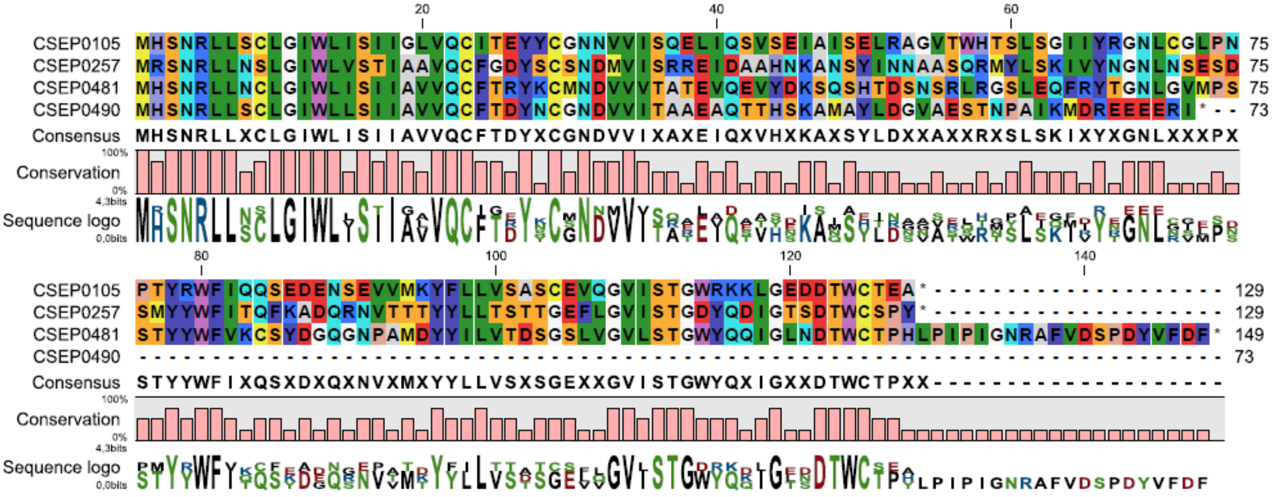

Additional file 14

CSEP family 32.

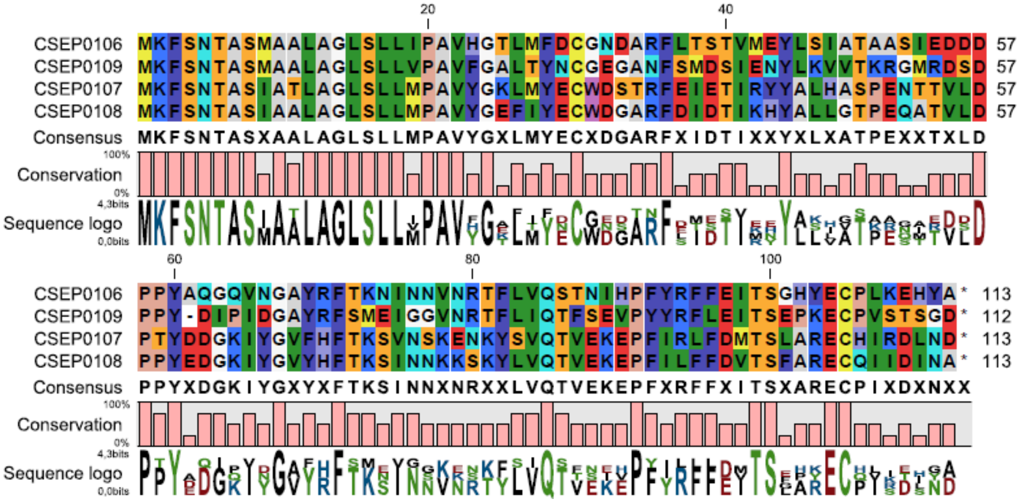

CSEP family 33.

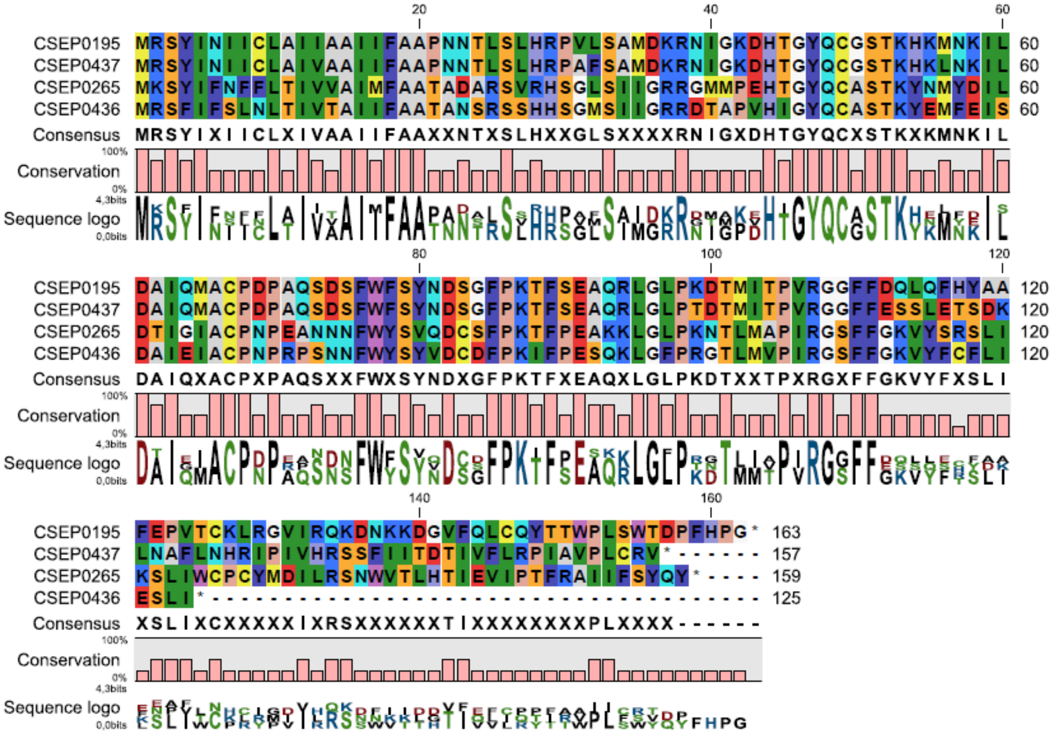

Additional file 14

CSEP family 34.

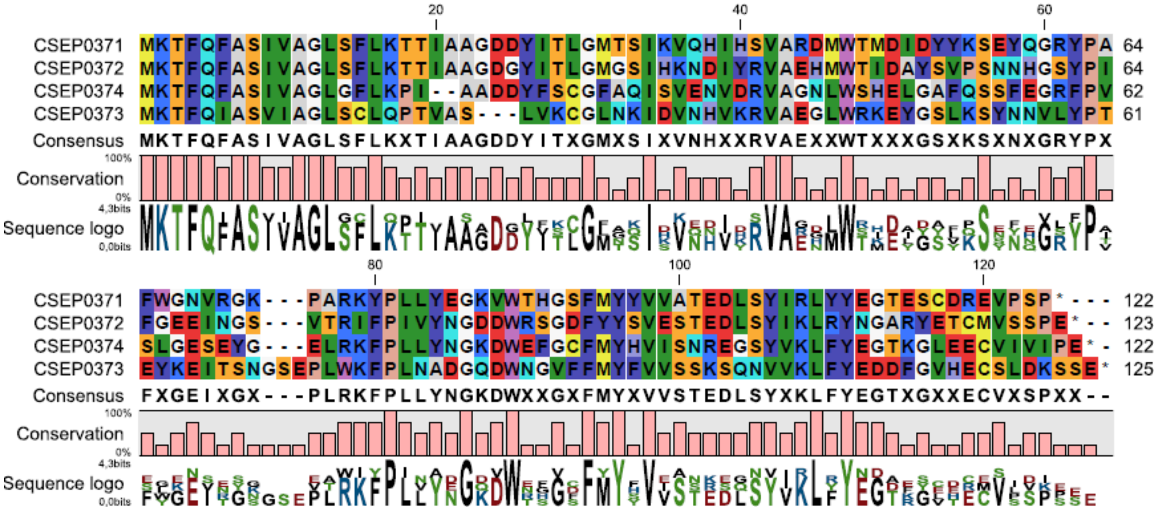

CSEP family 35.

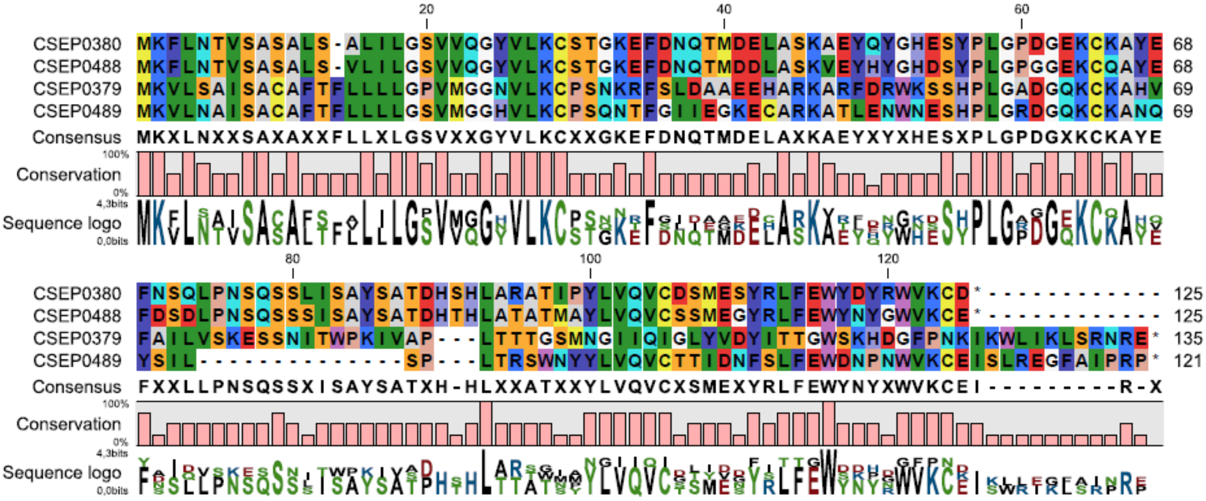

Additional file 14
