# Supplementary material for: Structure and evolution of barley powdery mildew effector candidates
Source: BMC Genomics. 2012 Dec 11;13:694. doi: 10.1186/1471-2164-13-694 (PMC3582587; doi:10.1186/1471-2164-13-694)
Supplement: Additional file 15 — Summary of data obtained for Blumeria and yeast data sets. This compilation is based on previously published data shown in grey [8] with the new sets and measures added. The CSEPs, Known_Fungal_Effectors and Haustoria_only sets have the lowest values in terms of: mean lengths, mean proportion disorder, mean maximum length of disorder, mean model quality and mean number of domains. In addition these sets have a higher proportion of top hits to ribonuclease and hydrolase structural templates. [file 1471-2164-13-694-S15.pdf]

Additional File 15. Summary of data obtained for Blumeria and yeast data sets.

The analysis is based on previously published data (shown in grey) [8] with the new sets and measures added. The highlighted cells indicate the values of interest. The CSEPs, Known_Fungal_Effectors and Haustoria_only sets have the lowest values in terms of: mean lengths, mean proportion disorder, mean maximum length of disorder, mean model quality and mean number of domains. In addition these sets have a higher proportion of top hits to ribonuclease and hydrolase structural templates.

Data type	Data set							
	CSEPs	Known_Fungal_Effectors	Haustoria_only	Hyphae_plus_Haustoria	Hyphae_only_random	Hyphae_only_length_dist	Yeast_random	Yeast_length
Mean length	208.762	**156.564**	236.507	422.397	407.169	237.676	614.578	236.521
Mean proportion of disorder	**0.149**	0.177	0.159	0.153	0.247	0.256	0.283	0.248
Mean maximum length of disordered region	18.324	**14.513**	36.127	31.397	54.014	35.056	86.239	35.211
Mean 3D model quality	**0.136**	0.152	0.177	0.354	0.314	0.314	0.190	0.274
Mean number of domains	1.546	**1.385**	1.521	2.588	2.451	1.563	3.465	1.901
Frequency of ribonucleases	0.114	0.000	**0.127**	0.021	0.028	0.014	0.056	0.028
Number of ribonucleases	56	0	9	4	2	1	4	2
Number of Non ribonucleases	435	39	62	190	69	70	67	69
Frequency of hydrolases	0.255	0.231	**0.324**	0.186	0.113	0.141	0.141	0.225
Number of hydrolases	125	9	23	36	8	10	10	16
Number of non-hydrolases	366	30	48	158	63	61	61	55
Total number of sequences	491	39	71	194	71	71	71	71

The lowest values in the first five rows are shown in bold. The highest frequencies of ribonucleases and hydrolases are also shown in bold. Grey cells indicate data reproduced from Bindschedler *et al*. [8].
