# Supplementary material for: Structure and evolution of barley powdery mildew effector candidates
Source: BMC Genomics. 2012 Dec 11;13:694. doi: 10.1186/1471-2164-13-694 (PMC3582587; doi:10.1186/1471-2164-13-694)
Supplement: Additional file 16 — Calculated p-values for unpaired Wilcoxon signed rank sum tests for the CSEP data set. The table shows the p-values for Wilcoxon signed rank sum significant tests for the CSEP set versus all other sets according to each data type (p<0.05 highlighted green). Footnote: The null hypothesis is that the data from each comparison set is equal to or lower in value than that from the CSEP set. The alternative hypothesis is that the data in the comparison set is greater in value. Significant p-values (p<0.05) are shown in bold. [file 1471-2164-13-694-S16.pdf]

Additional File 16. Calculated p-values for unpaired Wilcoxon signed rank sum tests for the CSEPs set data.

The table shows the p-values for Wilcoxon signed rank sum significant tests for the CSEPs set versus all other sets according to each data type ($p<0.05$). These data are very similar to those of Haustoria_only. For example, compared with the Hyphae_only set, all values are significantly lower. When we control for length however, all values are lower except for length and number of domains, as you might expect. There are few significant differences between CSEPs, Haustoria_only and Known_Fungal_Effectors sets - the p-values suggest that mean global model quality is significantly poorer still.

	Comparison set						
Data type	Known_Fungal_Effectors	Haustoria_only	Hyphae_plus_Haustoria	Hyphae_only_random	Hyphae_only_length_dist	Yeast_random	Yeast_length
Length	0.9941	0.7862	**0.00E+00**	**4.47E-09**	0.7094	**0.00E+00**	0.7859
Propotion of disorder	0.0537	0.5641	0.4194	**3.83E-05**	**4.41E-07**	**1.42E-08**	**4.31E-08**
Maximum length of disordered region	0.7309	0.3795	**1.23E-12**	**6.24E-11**	**6.77E-08**	**0.00E+00**	**7.79E-08**
3D model quality	**0.0225**	**0.0007**	**0.00E+00**	**9.30E-14**	**0.00E+00**	0.1624	**3.00E-15**
Number of domains	0.7791	0.8438	**0.00E+00**	**1.20E-06**	0.5899	**0.00E+00**	**0.0347**

The null hypothesis is that the data from each comparison set is equal to or lower in value than that from the CSEPs set. The alternative hypothesis is that the data in the comparison set is greater in value. Statistically significant p-values (<0.05) are shown in bold.
