# Supplementary material for: Structure and evolution of barley powdery mildew effector candidates
Source: BMC Genomics. 2012 Dec 11;13:694. doi: 10.1186/1471-2164-13-694 (PMC3582587; doi:10.1186/1471-2164-13-694)
Supplement: Additional file 17 — Calculated p-values for Fisher's exact tests for the CSEP data set compared against data from all other sets. Shown are the categorical data regarding the proportion of ribonucleases and hydrolases analysed using a Fisher’s exact test (again, p<0.05 highlighted green). Footnote: p-values (p<0.05) are shown in bold, indicating significant over representation of the data type in the CSEP set. [file 1471-2164-13-694-S17.pdf]

Additional File 17. Calculated p-values for Fisher's exact tests for the CSEPs set data compared against data from all other sets.

The categorical data regarding the proportion of ribonucleases and hydrolases analysed using a Fisher’s exact test (p<0.05). ribonucleases are significantly overrepresented in CSEPs compared with all other sets apart from Haustoria_only and Yeast_random where there is no measurable difference with this data. Hydrolases are significantly over represented in CSEPs compared with Hyphae_only_random, Hyphae_only_length_dist and Yeast_random, but there is no measurable difference between CSEPs and Haustoria_only, Known_Fungal_Effectors, Hyphae_plus_Haustoria and Yeast_length.

Key words	Comparison set						
	Known_Fungal_Effectors	Haustoria_only	Hyphae_plus_Haustoria	Hyphae_only_random	Hyphae_only_length_dist	Yeast_random	Yeast_length
Ribonucleases	**0.0253**	0.6949	**3.24E-05**	**0.0215**	**0.0053**	0.2143	**0.0215**
Hydrolases	0.8495	0.2484	0.0578	**0.0070**	**0.0375**	**0.0375**	0.6622

Statistically significant p-values (<0.05) are shown in bold
