# Supplementary material for: Structure and evolution of barley powdery mildew effector candidates
Source: BMC Genomics. 2012 Dec 11;13:694. doi: 10.1186/1471-2164-13-694 (PMC3582587; doi:10.1186/1471-2164-13-694)
Supplement: Additional file 18 — IntFOLD 3D models for selected CSEP families. A: IntFOLD 3D models for CSEPs from family 12. Positively selected residues are highlighted in red. Left, cartoon view showing secondary structure types. Right, surface view showing globular structure. Images were rendered using PyMol. B: IntFOLD 3D models for CSEPs from family 22. Positively selected residues are highlighted in red. Left, cartoon view showing secondary structure types. Right, surface view showing globular structure. Images were rendered using PyMol. C: IntFOLD 3D models for CSEPs from family 5. Positively selected residues are highlighted in red. Left, cartoon view showing secondary structure types. Right, surface view showing globular structure. Images were rendered using PyMol. D: IntFOLD 3D models for CSEPs from family 21. Positively selected residues are highlighted in red. Left, cartoon view showing secondary structure types. Right, surface view showing globular structure. Images were rendered using PyMol. E: IntFOLD 3D models for CSEPs from family 23. Positively selected residues are highlighted in red. Left, cartoon view showing secondary structure types. Right, surface view showing globular structure. Images were rendered using PyMol.A. [file 1471-2164-13-694-S18.pdf]

Additional File 18

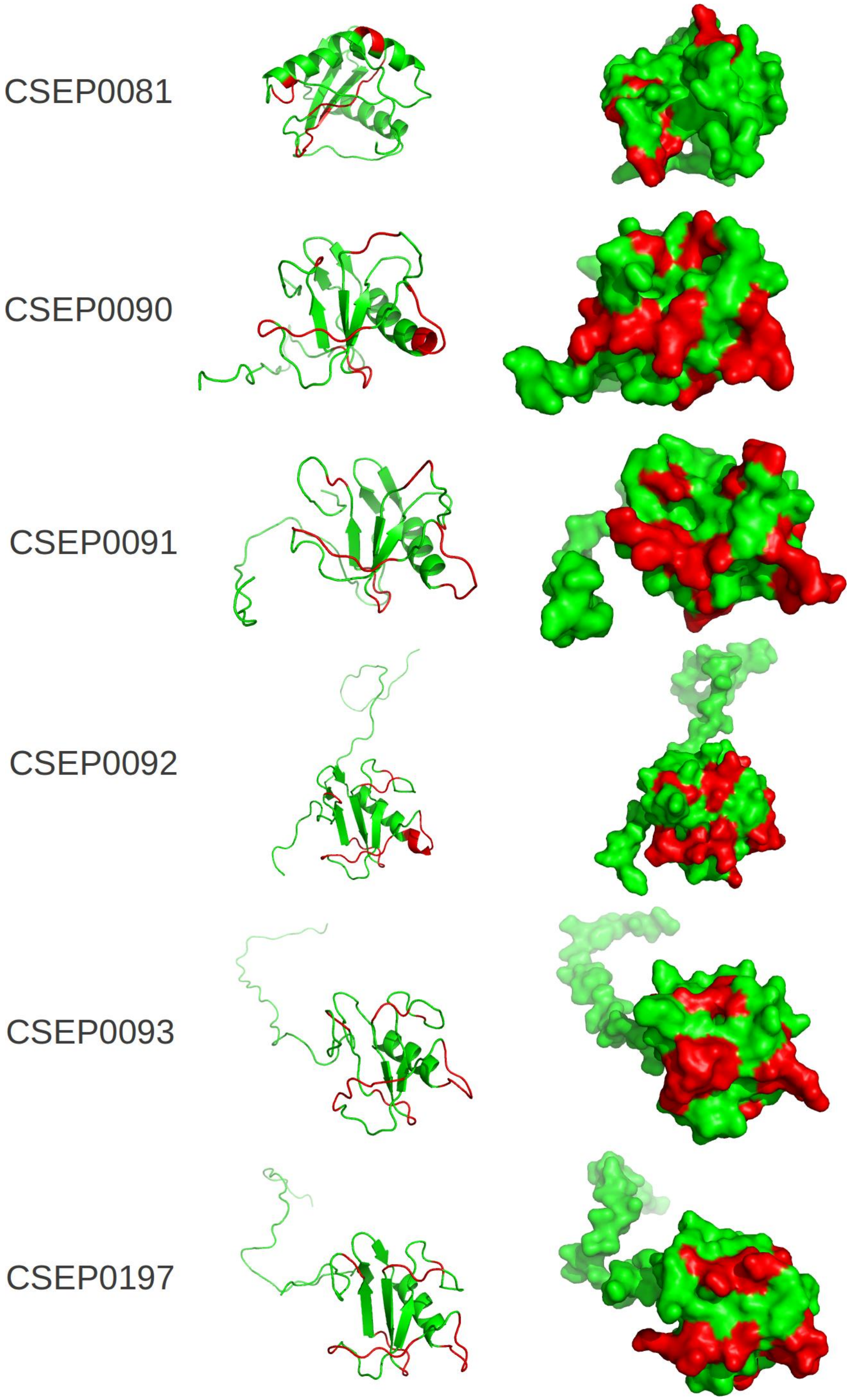

Additional File 18A: Family 12

CSEP0137

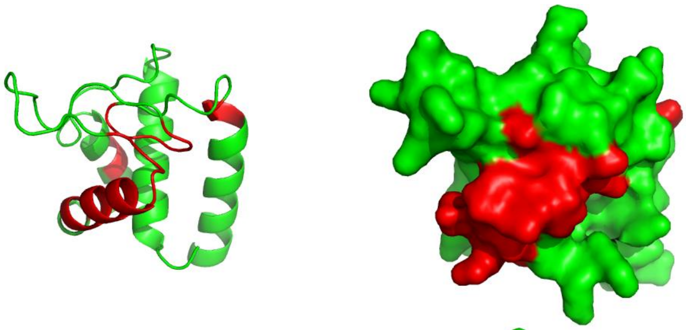

CSEP0146

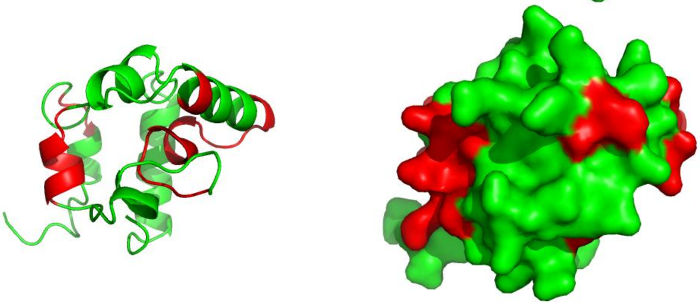

CSEP0180

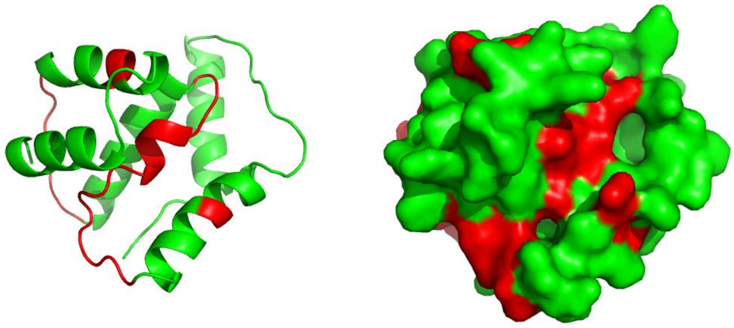

CSEP0181

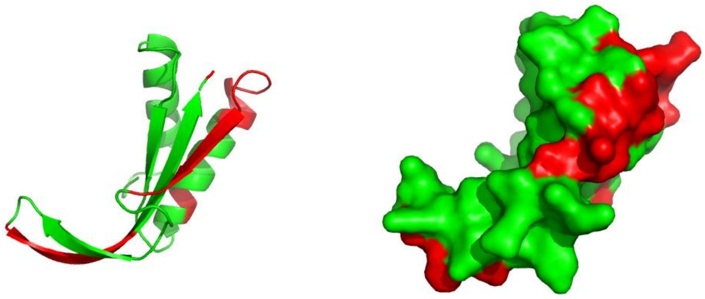

CSEP0267

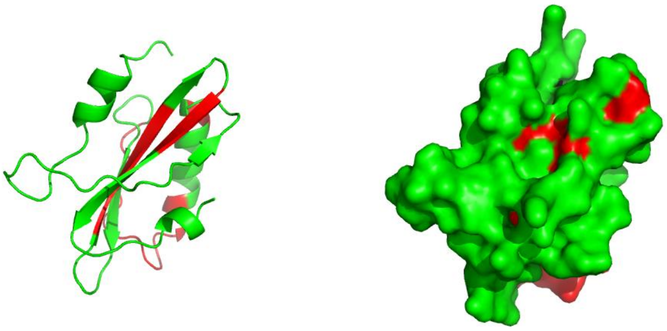

Additional File 18B: Family 21

CSEP0117

CSEP0114

CSEP0113

CSEP0099

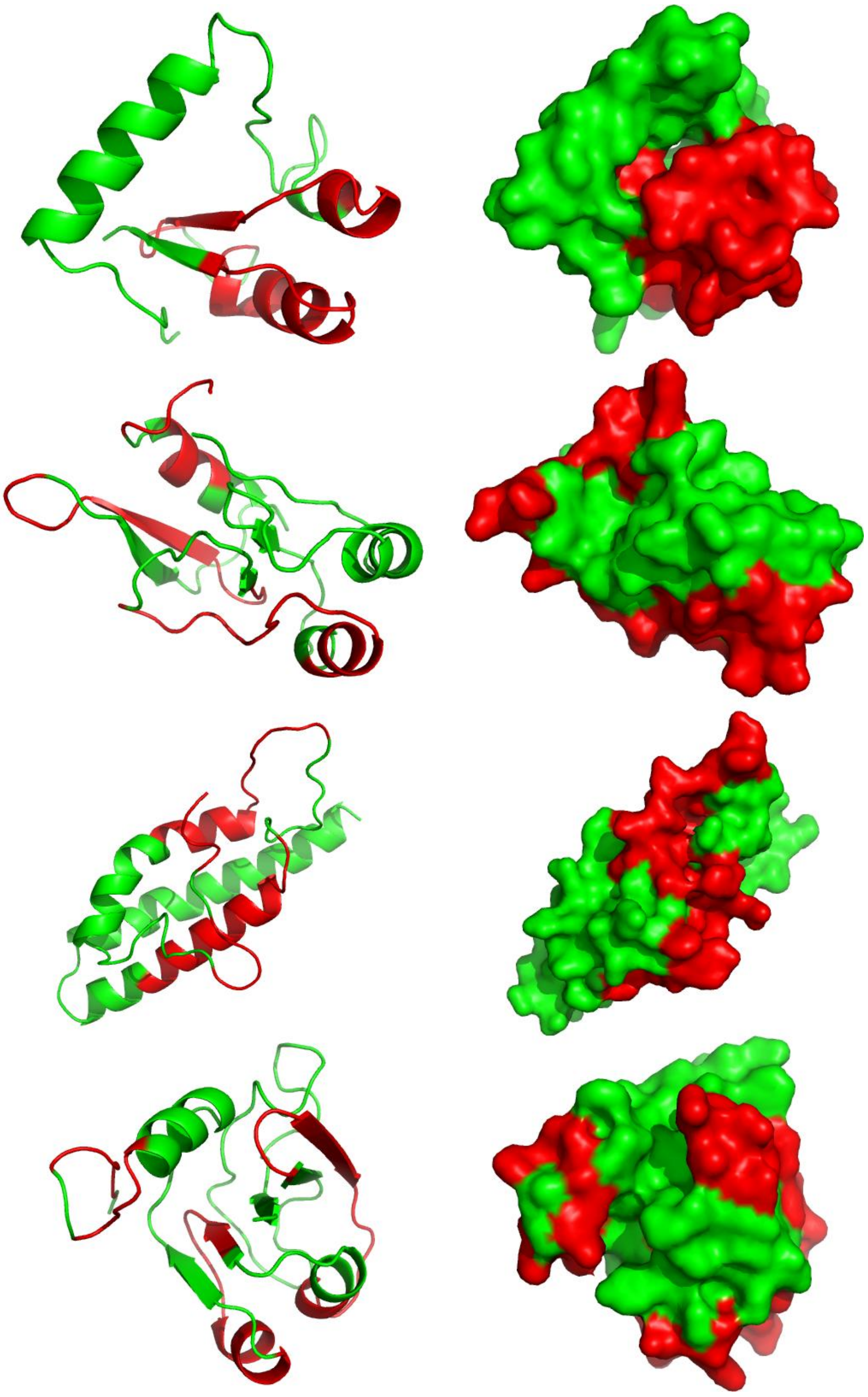

Additional File 18 C: Family 5

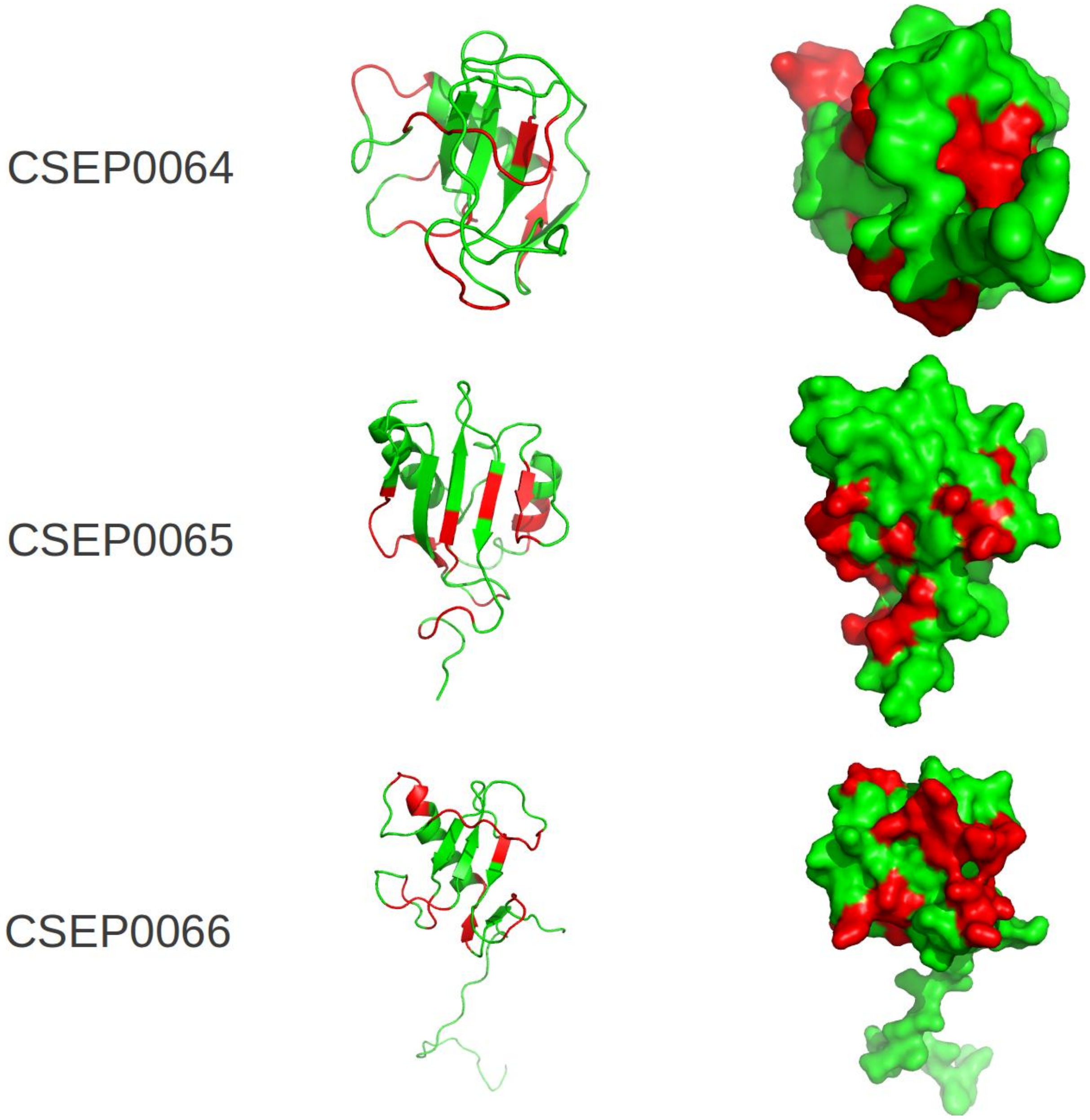

Additional File 18 D: Family 22

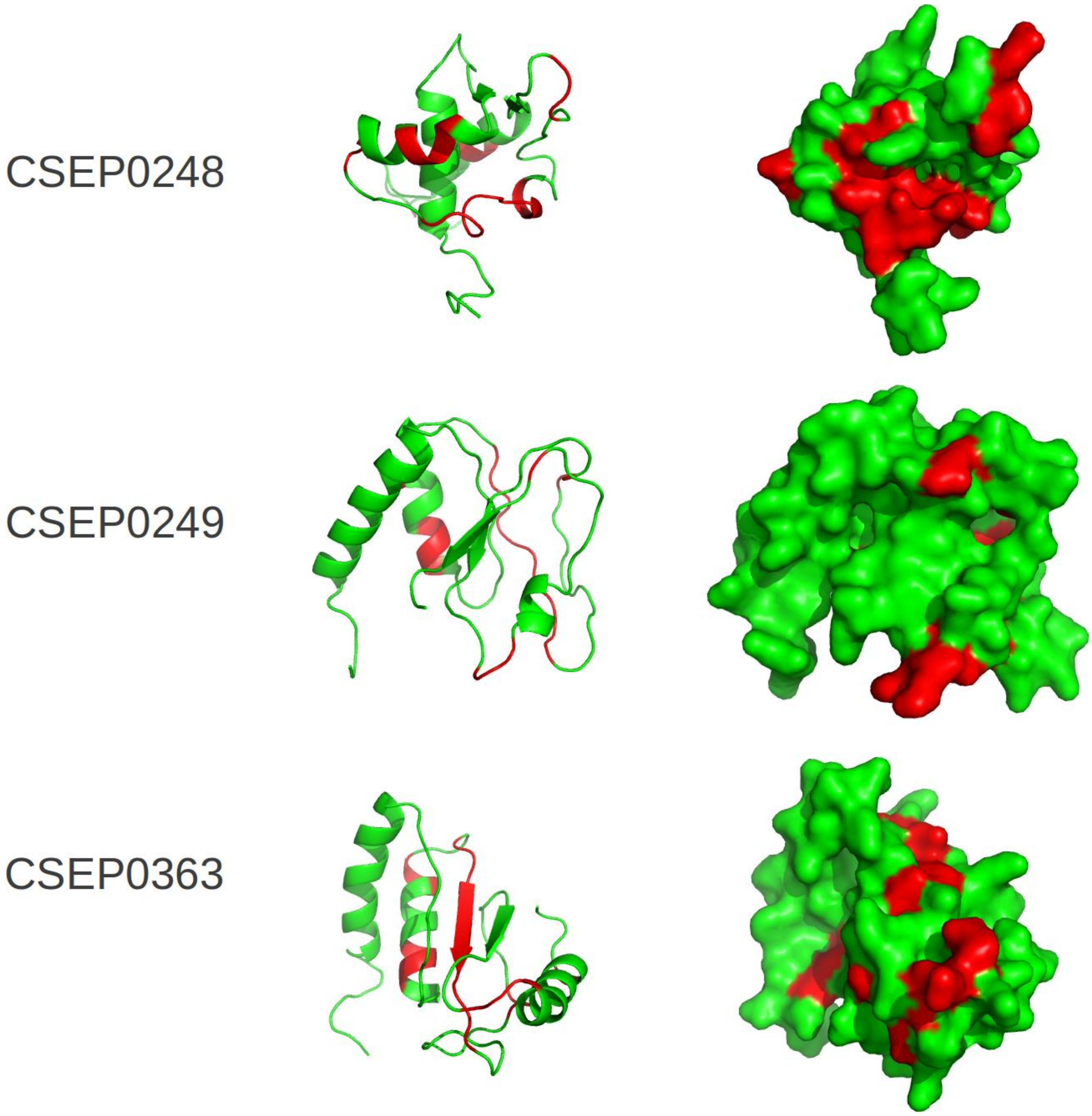

Additional File 18 E: Family 23
