# Supplementary material for: Structure and evolution of barley powdery mildew effector candidates
Source: BMC Genomics. 2012 Dec 11;13:694. doi: 10.1186/1471-2164-13-694 (PMC3582587; doi:10.1186/1471-2164-13-694)
Supplement: Additional file 19 — CSEPs show significant differences in amino acid frequencies and secondary structure (part 1). The CSEP set compared with other sets according to: length (as a control), amino acid frequency (A-Y), coiled-coil composition, TM helix composition (as a control), low complexity regions, frequency of helical residues, frequency of strand residues, frequency of loop residues. The Haustoria_only set is compared with other sets according to: length (as a control), amino acid frequency (A-Y), coiled-coil composition, TM helix composition, low complexity regions, frequency of helical residues, frequency of strand residues, frequency of loop residues. The null hypothesis is that the Haustoria_set has greater frequencies of that in each column than the set. [file 1471-2164-13-694-S19.pdf]

Additional File 19. CSEP proteins show significant differences in amino acid frequencies and secondary structure (part 1). The CSEPs set compared with other sets according to: length (as a control), amino acid frequency (A-Y), coiled-coil composition, TM helix composition (as a control), low complexity regions, frequency of helical residues, frequency of strand residues, frequency of loop residues. The Haustoria_only set is compared with other sets according to: length (as a control), amino acid frequency (A-Y), coiled-coil composition, TM helix composition, low complexity regions, frequency of helical residues, frequency of strand residues, frequency of loop residues. The null hypothesis is that the Haustoria_set has greater frequencies of that in each column than the set indicated in the row. (Statistically significant p-values (<0.05) are shown in bold, prominent data columns are highlighted grey.

Set_name	Length	A	C	D	E	F	G	H	I	K	L	M	N	P
yeast_length_dist	0.2126	0.8993	**4.80E-13**	0.9876	1.0000	**3.93E-06**	0.1480	0.0834	**0.0113**	1.0000	0.9755	0.1571	0.9845	**0.0135**
yeast_sample_dist	1.0000	0.2204	**1.50E-14**	0.9904	0.9999	**0.0021**	**0.0069**	0.2063	0.2582	0.9999	0.9996	**0.0292**	0.9994	0.4100
Hyphae_plus_Haustoria_IDs_length_dist	0.8020	1.0000	**2.29E-13**	0.9907	1.0000	**1.63E-09**	1.0000	**0.0004**	**0.0067**	0.9966	0.2916	**0.0451**	**7.30E-06**	**0.0002**
Hyphae_plus_Haustoria_sample_dist	1.0000	1.0000	**0.00E+00**	1.0000	1.0000	**4.03E-08**	1.0000	**0.0005**	**0.0345**	0.9999	0.2982	**0.0308**	**6.46E-06**	**0.0304**
Hyphae_only_length_dist	0.2887	1.0000	**9.00E-15**	0.9706	1.0000	**0.00E+00**	0.9941	**0.0206**	**0.0002**	1.0000	0.7203	0.3240	**9.38E-06**	0.4788
Hyphae_only_sample_dist	1.0000	1.0000	**2.00E-15**	0.9928	1.0000	**9.77E-13**	0.9999	**0.0023**	**0.0430**	0.9668	0.9296	0.3071	**0.0001**	0.8516
proteome_minus_CSEPs	1.0000	1.0000	**0.00E+00**	0.9946	1.0000	**0.00E+00**	0.9686	**0.0173**	**2.00E-15**	0.7822	1.0000	**3.51E-09**	**0.00E+00**	1.0000
Known_Fungal_Effectors	0.0059	1.0000	0.9977	0.8989	0.5093	**0.0001**	1.0000	0.8504	**0.0001**	**0.0344**	**0.0001**	**0.0004**	0.3445	0.9307
Haustoria_only	0.2126	0.9997	0.8557	0.9935	0.6228	**0.0386**	0.9903	**0.0107**	**0.0042**	**1.13E-06**	**0.0001**	**0.0011**	0.5128	**0.0390**

Additional File 20 continued

Set_name	Q	R	S	T	V	W	Y	Coiled coils	TM	Low Complexity	Helix	Strand	Loops
yeast_length_dist	0.7498	**0.0103**	**1.21E-05**	0.3056	0.8964	0.0525	**1.19E-08**	0.1014	1.0000	0.9977	0.7387	1.0000	**6.76E-06**
yeast_sample_dist	0.9803	**0.0331**	**0.0527**	0.7463	0.1979	0.2988	**4.47E-07**	0.9801	1.0000	1.0000	1.0000	0.9791	**8.51E-07**
Hyphae_plus_Haustoria_IDs_length_dist	0.7899	0.4826	**0.0454**	0.0877	0.9995	**0.0122**	**2.32E-08**	**0.0124**	0.9993	0.8447	0.3309	**0.00E+00**	**0.00E+00**
Hyphae_plus_Haustoria_sample_dist	0.1539	**0.0045**	**0.0001**	0.3244	0.9999	0.2614	**2.21E-13**	**0.0376**	0.9880	0.6133	0.9826	**0.00E+00**	**0.00E+00**
Hyphae_only_length_dist	0.6718	0.9295	**0.0019**	0.6692	0.9797	0.2095	**0.00E+00**	**0.0423**	1.0000	0.8440	0.7483	0.9978	**0.0081**
Hyphae_only_sample_dist	0.9950	0.9830	**0.0113**	0.3201	0.7909	**0.0186**	**0.00E+00**	0.1044	1.0000	0.3216	0.7320	1.0000	**7.79E-07**
proteome_minus_CSEPs	1.0000	1.0000	0.9402	0.9924	**0.0024**	0.9999	**0.00E+00**	0.9935	1.0000	0.9872	-	-	-
Known_Fungal_Effectors	0.1348	0.2735	**0.0009**	0.8450	0.1918	0.9930	**0.0138**	0.9996	1.0000	1.0000	1.0000	0.2268	0.5447
Haustoria_only	0.9983	**0.0410**	0.3943	0.9998	0.9975	0.9875	0.5616	0.1009	0.2967	0.6253	0.8842	0.6896	0.8827
