# Supplementary material for: Structure and evolution of barley powdery mildew effector candidates
Source: BMC Genomics. 2012 Dec 11;13:694. doi: 10.1186/1471-2164-13-694 (PMC3582587; doi:10.1186/1471-2164-13-694)
Supplement: Additional file 20 — CSEPs show significant differences in amino acid frequencies and secondary structure (part 2). The CSEP set compared with other sets according to: length (as a control), amino acid frequency (A-Y), coiled-coil composition, TM helix composition, low complexity regions, frequency of helical residues, frequency of strand residues, frequency of loop residues. The table contains the same information as Additional file 19 but with the reverse null hypothesis (or 1-p). [file 1471-2164-13-694-S20.pdf]

Additional File 20. CSEP proteins show significant differences in amino acid frequencies and secondary structure (part 2). The CSEPs set compared with other sets according to: length (as a control), amino acid frequency (A-Y), coiled-coil composition, TM helix composition, low complexity regions, frequency of helical residues, frequency of strand residues, frequency of loop residues. The table contains the same information as Additional File 20 but with the reverse null hypothesis (or 1-p).

Set_name	Length	A	C	D	E	F	G	H	I	K	L	M	N	P
yeast_length_dist	0.7874	0.1007	1.0000	**0.0124**	**2.41E-05**	1.0000	0.8520	0.9166	0.9887	**1.00E-09**	**0.0245**	0.8429	0.0155	0.9865
yeast_sample_dist	**0.00E+00**	0.7796	1.0000	**0.0096**	**0.0001**	0.9979	0.9931	0.7937	0.7418	**0.0001**	**0.0004**	0.9708	0.0006	0.5900
Hyphae_plus_Haustoria_IDs_length_dist	0.1980	**0.00E+00**	1.0000	**0.0093**	**1.00E-10**	1.0000	**2.60E-05**	0.9996	0.9933	**0.0034**	0.7084	0.9549	1.0000	0.9998
Hyphae_plus_Haustoria_sample_dist	**0.00E+00**	**0.00E+00**	1.0000	**1.83E-05**	**0.00E+00**	1.0000	**4.00E-10**	0.9995	0.9655	**0.0001**	0.7018	0.9692	1.0000	0.9696
Hyphae_only_length_dist	0.7113	**6.70E-09**	1.0000	**0.0294**	**1.23E-06**	1.0000	**0.0059**	0.9794	0.9998	**5.33E-06**	0.2797	0.6760	1.0000	0.5212
Hyphae_only_sample_dist	**4.30E-09**	**1.48E-08**	1.0000	**0.0072**	**0.00E+00**	1.0000	**0.0001**	0.9977	0.9570	**0.0332**	0.0704	0.6929	0.9999	0.1484
proteome_minus_CSEPs	**0.00E+00**	**0.00E+00**	1.0000	**0.0054**	**0.00E+00**	1.0000	**0.0314**	0.9827	1.0000	0.2178	**9.10E-08**	1.0000	1.0000	**0.00E+00**
Known_Fungal_Effectors	0.9941	**2.70E-06**	**0.0023**	0.1011	0.4907	0.9999	**2.75E-05**	0.1496	0.9999	0.9656	0.9999	0.9996	0.6555	0.0693
Haustoria_only	0.7874	**0.0003**	0.1443	**0.0065**	0.3772	0.9614	**0.0097**	0.9893	0.9958	1.0000	0.9999	0.9989	0.4872	0.9610

Additional File 22. continued

Set_name	Q	R	S	T	V	W	Y	Coiled coils	TM	Low Complexity	Helix	Strand	Loops
yeast_length_dist	0.2502	0.9897	1.0000	0.6944	0.1036	0.9475	1.0000	0.8986	**1.16E-06**	**0.0023**	0.2613	**5.61E-07**	1.0000
yeast_sample_dist	**0.0197**	0.9669	0.9473	0.2537	0.8021	0.7012	1.0000	**0.0199**	**0.00E+00**	**6.52E-08**	**1.55E-07**	**0.0209**	1.0000
Hyphae_plus_Haustoria_IDs_length_dist	0.2101	0.5174	0.9546	0.9123	**0.0005**	0.9878	1.0000	0.9876	**0.0007**	0.1553	0.6691	1.0000	1.0000
Hyphae_plus_Haustoria_sample_dist	0.8461	0.9955	0.9999	0.6756	**0.0001**	0.7386	1.0000	0.9624	**0.0120**	0.3867	**0.0174**	1.0000	1.0000
Hyphae_only_length_dist	0.3282	0.0705	0.9981	0.3308	**0.0203**	0.7905	1.0000	0.9577	**1.40E-09**	0.1560	0.2517	**0.0022**	0.9919
Hyphae_only_sample_dist	**0.0050**	**0.0170**	0.9887	0.6799	0.2091	0.9814	1.0000	0.8956	**3.06E-05**	0.6784	0.2680	**5.89E-06**	1.0000
proteome_minus_CSEPs	**3.49E-07**	**0.00E+00**	0.0598	**0.0076**	0.9976	**0.0001**	1.0000	**0.0065**	**9.00E-10**	**0.0128**	-	-	-
Known_Fungal_Effectors	0.8652	0.7265	0.9991	0.1550	0.8082	**0.0070**	0.9862	**0.0004**	**0.00E+00**	**3.90E-09**	**1.83E-08**	0.7732	0.4553
Haustoria_only	**0.0017**	0.9590	0.6057	**0.0002**	**0.0025**	**0.0125**	0.4384	0.8991	0.7033	0.3747	0.1158	0.3104	0.1173
