# Supplementary material for: Structure and evolution of barley powdery mildew effector candidates
Source: BMC Genomics. 2012 Dec 11;13:694. doi: 10.1186/1471-2164-13-694 (PMC3582587; doi:10.1186/1471-2164-13-694)
Supplement: Additional file 23 — Clustering of CSEPs on sequence scaffolds. The Table shows for each of the studied families how many members are clustered and the length of the scaffold region containing the members. The scaffold length includes both the sum of the sequence contigs and the calculated distances between the contigs. The average distance is the distance between two CSEPs on the scaffold if they were distributed evenly. [file 1471-2164-13-694-S23.pdf]

Additional File 23. Clustering of *CSEPs* on sequence scaffolds. The table shows for each of the studied families how many members there are clustered and the length of the scaffold-region containing the members. The scaffold length includes both the sum of sequences contigs and the calculated distances between the contigs. The average distance is the distance between two *CSEPs* on the scaffold if they were distributed evenly.

Family	Members	Clustered	Clustered within (kb)	Average distance (kb)	Closest pair (kb)
1	59	3	1882	941	*CSEP0312* and *CSEP0313*: ~ 44 kb
1	59	2	326	326	*CSEP0330* and *CSEP0222*: ~ 326 kb
1	59	5	3954	989	*CSEP0336* and *CSEP0337*: ~ 90 kb
1	59	2	86	86	*CSEP0345* and *CSEP0346*: ~ 86 kb
1	59	2	80	80	*CSEP0349* and *CSEP0351*: ~ 80 kb
1	59	4	1288	429	*CSEP0404* and *CSEP0405*: ~ 5 kb
1	59	7	655	109	*CSEP0413* and *CSEP0414*: ~ 13 kb
1	59	7	1028	171	*CSEP0423* and *CSEP0424*: ~ 4 kb
1	59	2	8	8	*CSEP0079* and *CSEP0215*: ~ 8 kb
1	59	5	213	53	*CSEP0450* and *CSEP0013*: ~ 19 kb
1	59	7	209	35	*CSEP0456* and *CSEP0457*: ~ 11 kb
1	59	2	10	10	*CSEP0478* and *CSEP0479*: ~ 10 kb
2	32	18	1429	84	*CSEP0011* and *CSEP0021*: ~16 kb
2	32	3	217	109	*CSEP0375* and *CSEP0376*: ~22 kb
2	32	2	40	40	*CSEP0245* and *CSEP0246*: ~40 kb
3	20	7	365	61	*CSEP0217* and *CSEP0218*: ~10 kb
3	20	6	324	65	*CSEP0158* and *CSEP0461*: ~22 kb
3	20	4	133	44	*CSEP0236* and *CSEP0391*: ~12 kb
4	19	8	420	60	*CSEP0089* and *CSEP0153*: ~5 kb
4	19	5	217	54	*CSEP0164* and *CSEP0166*: ~33 kb
4	19	4	143	48	*CSEP0036* and *CSEP0171*: ~44 kb
5	15	4	327	109	*CSEP0113* and *CSEP0442*: ~3 kb
5	15	3	40	20	*CSEP0114* and *CSEP0115*: ~6 kb
5	15	2	42	42	*CSEP0339* and *CSEP0112*: ~42 kb
5	15	2	4	4	*CSEP0117* and *CSEP0170*: ~4 kb
5	15	2	83	83	*CSEP0099* and *CSEP0393*: ~83 kb
6	10	10	534	59	*CSEP0015* and *CSEP0467*: ~3 kb
7	10	2	107	107	*CSEP0279* and *CSEP0280*: ~107 kb
7	10	6	344	69	*CSEP0305* and *CSEP0306*: ~25 kb
8	8	5	744	186	*CSEP0149* and *CSEP0150*: ~82 kb
8	8	3	65	33	*CSEP0058* and *CSEP0333*: ~16 kb
9	7	6	1233	247	*CSEP0291* and *CSEP0292*: ~4 kb

10	7	6	943	189	*CSEP0032* and *CSEP0269*: ~25 kb
11	7	6	660	132	*CSEP0240* and *CSEP0160*: ~45 kb
12	7	4	244	81	*CSEP0093* and *CSEP0094*: ~6 kb
12	7	3	180	90	*CSEP0090* and *CSEP0091*: ~116 kb
13	7	5	225	56	*CSEP0025* and *CSEP0069*: ~3 kb
14	7	2	210	210	*CSEP0364* and *CSEP0365*: ~210 kb
15	7	3	9	5	*CSEP0416* and *CSEP0417*: ~2,5 kb
16	6	5	170	43	*CSEP0128* and *CSEP0344*: 2,5 kb
23	5	3	169	85	*CSEP0248* and *CSEP0363*: ~29 kb
25	4	4	127	42	*CSEP0005* and *CSEP0012*: ~6 kb
30	4	4	446	149	*CSEP0069* and *CSEP0070*: ~98 kb
32	4	2	31	31	*CSEP0107* and *CSEP0108*: ~31 kb
32	4	2	54	54	*CSEP0106* and *CSEP0109*: ~54 kb
33	4	4	268	89	*CSEP0265* and *CSEP0436*: ~36 kb
52	3	3	120	60	*CSEP0255* and *CSEP0296*: ~41 kb
Average:			434	129	
